# Continual reproduction of self-assembling oligotriazole peptide nanomaterials

**DOI:** 10.1038/s41467-017-00849-1

**Published:** 2017-09-28

**Authors:** Roberto J. Brea, Neal K. Devaraj

**Affiliations:** Department of Chemistry and Biochemistry, University of California, 9500 Gilman Drive, San Diego, CA 92093 USA

## Abstract

Autocatalytic chemical reactions, whereby a molecule is able to catalyze its own formation from a set of precursors, mimic nature’s ability to generate identical copies of relevant biomolecules, and are thought to have been crucial for the origin of life. While several molecular autocatalysts have been previously reported, coupling autocatalytic behavior to macromolecular self-assembly has been challenging. Here, we report a non-enzymatic and chemoselective methodology capable of autocatalytically producing triskelion peptides that self-associate into spherical bioinspired nanostructures. Serial transfer experiments demonstrate that oligotriazole autocatalysis successfully leads to continual self-assembly of three-dimensional nanospheres. Triskelion-based spherical architectures offer an opportunity to organize biomolecules and chemical reactions in unique, nanoscale compartments. The use of peptide-based autocatalysts that are capable of self-assembly represents a promising method for the development of self-synthesizing biomaterials, and may shed light on understanding life’s chemical origins.

## Abstract (web summary)

Molecules that act as both autocatalysts and material precursors offer exciting prospects for self-synthesizing materials. Here, the authors design a triazole peptide that self-replicates and then self-assembles into nanostructures, coupling autocatalytic and assembly pathways to realize a reproducing supramolecular system.

## Introduction

One of the defining features of life is self-reproduction, which occurs both on the molecular scale through the replication of genetic polymers such as DNA and RNA, as well as larger scales through the reproduction of cells and multicellular life forms^1, 2^. While modern living organisms are extremely complex molecular assemblies that are the product of billions of years of evolution, there has been significant interest in developing simpler self-replicating systems^3–8^. Such minimal self-reproducing molecules and assemblies could have relevance to questions regarding the origin of life, shedding light on the underlying principles that led to the first living systems on Earth. Given the central role of self-replication in life^1, 2^, there has been a long-standing interest in autocatalytic systems, where the product of a reaction acts as the catalyst for its formation^9^. In seminal studies, investigators mimicked biological replication by developing and studying peptide-based structures that can self-reproduce in a template dependent manner^10–12^. Peptide self-reproduction is extremely selective, can be used for chiral selection, and multiple peptide replicators can form organized networks with characteristics similar to networks found in biology^13, 14^. While there have been several examples of molecular self-replicators or autocatalytic ligands^10–12^, it has been challenging to link autocatalytic behavior to self-assembly^10, 11^. A conceptually simple route would be the use of an autocatalytic coupling catalyst that also serves as a material building block.

Here we show a straightforward methodology to generate functional oligotriazole peptides capable of self-assembling into bionanomaterials. The use of a copper catalyzed azide-alkyne cycloaddition (CuAAC)^15^ successfully drives the autocatalytic production of triskelion-based peptides that self-associate into spherical bioinspired nanostructures. Triskelion-based spherical architectures offer a unique opportunity to organize biomolecules and reactions in nanoscale compartments. Additionally, serial transfer experiments demonstrate that CuAAC-based autocatalysis and consequent self-assembly of oligotriazole peptides leads to continual self-propagation of the corresponding three-dimensional peptide nanospheres in the presence of appropriate precursors. We also observe differential reactivity of precursors during oligotriazole synthesis, which facilitate the selective formation of self-assembling oligotriazole peptides. Our non-enzymatic and chemoselective methodology brings the opportunity to couple autocatalysis and self-assembly, which represents a promising strategy for the development of novel self-reproducing biomaterials based on peptide architectures.

## Results

### Synthesis and characterization of oligotriazole peptides

**Fig. 1:**
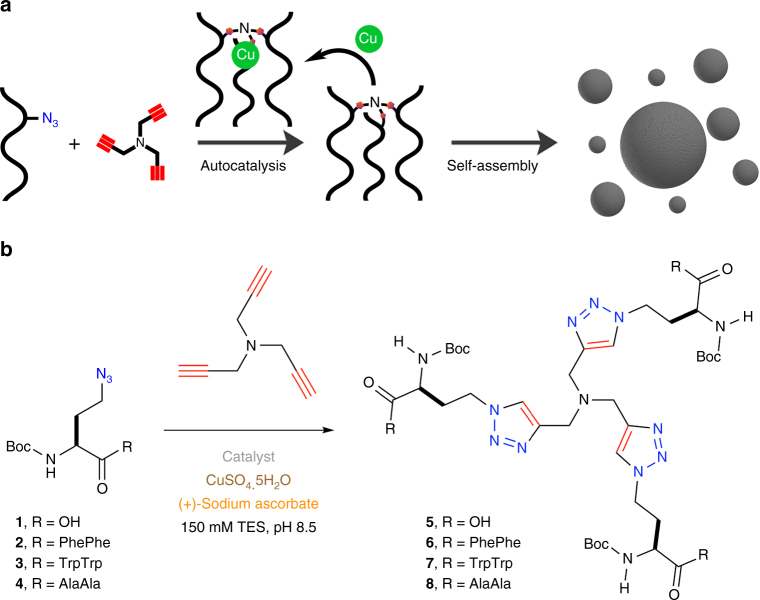
Self-assembling autocatalysts. **a** Copper-dependent autocatalytic oligotriazole formation leads to continuous generation of triskelion peptides, which spontaneously assemble into nanospheres. **b** Synthesis of tris(triazole) tripodal peptides by conjugation of a tripropargylamine scaffold with azide-modified peptides

Inspired by the importance of the triskelion architecture in facilitating the formation of well-ordered cagelike nanostructures^16, 17^, we decided to explore whether tris(triazole) peptides could self-assemble into spherical nanoarchitectures (Fig. 1a). Oligotriazole Cu^1+^ complexes can catalyze triazole formation^18^, and therefore autocatalytic reproduction of tris(triazole) peptides may be feasible.

Consequently, we first synthesized a series of oligotriazole peptides by conjugation of a tripropargylamine scaffold with azidopeptide units (Fig. 1b). Azide-containing building blocks were prepared from N-Boc protected azidohomoalanine using standard solution-phase peptide synthesis protocols^19, 20^. Modification of tripropargylamine with azide-modified peptides by (CuAAC)^20^ resulted in the formation of tripodal tris(triazole) ligands possessing three peptide arms. For our initial studies, we focused on tripodal conjugates between tripropargylamine and unmodified azidohomoalanine (^H^Ala **5**), as well as azidohomoalanine elaborated with an aromatic dipeptide (^H^AlaPhePhe **6** or ^H^AlaTrpTrp **7**) or hydrophobic dipeptide (^H^AlaAlaAla **8**).

**Fig. 2:**
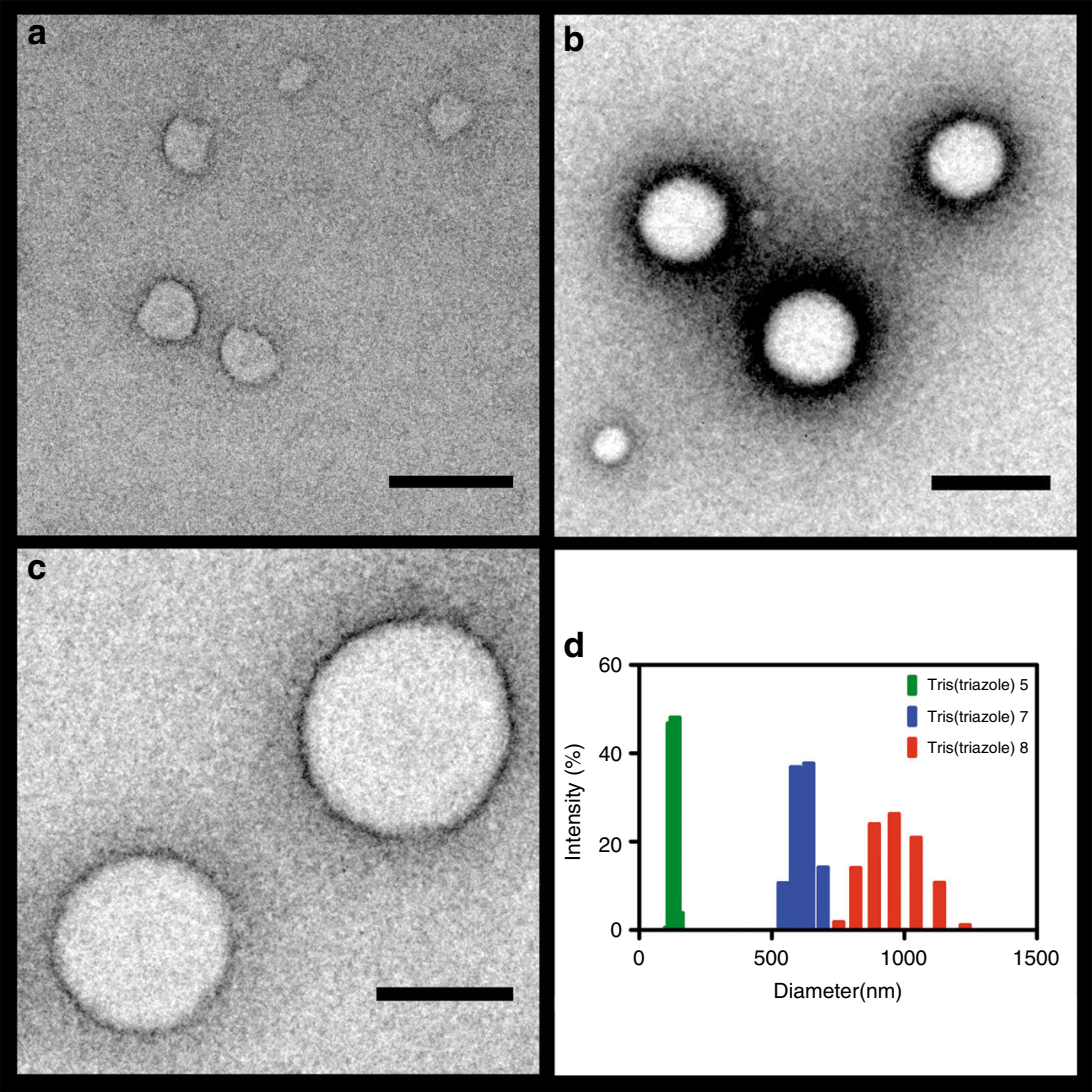
Characterization of peptide nanospherical structures. TEM images of negatively stained self-assembled peptide nanospheres formed from oligotriazole peptide **5** (**a**), **7** (**b**), and **8** (**c**). *Scale bar* denotes 500 nm. **d** DLS corresponding to 10 mM aqueous solution of tris(triazole) peptide **5** (*green*), **7** (*blue*), and **8** (*red*)

Transmission electron microscopy (TEM) revealed the presence of several populations of spherical compartments (150, 600 and 900 nm average diameter for **5**, **7**, and **8**, respectively), consistent with nanosphere architectures (Fig. 2a–c). The size of the particles was narrowly distributed and dependent on the peptide sequence. High-resolution cryo-TEM studies were also performed and verified the presence of spherical structures (Supplementary Fig. 1).

Alternatively, nanospherical architectures of the azido-modified peptide precursors **1**, **3**, and **4** were not observable by TEM under the same hydration conditions (Supplementary Fig. 2), demonstrating that peptide tris(triazole) formation was necessary to trigger sphere assembly. Dynamic light-scattering (DLS) experiments of the hydrated oligotriazole samples corroborated the nanostructure sizes (Fig. 2d, Supplementary Fig. 3). The structure of the peptide arm on the tripodal ligand appears to have a strong influence on the size of the corresponding self-assembled nanospheres. Interestingly, nanosphere formation also took place in organic-aqueous solutions of methanol and ethanol (50 wt. %) (Supplementary Fig. 4), suggesting that nanomaterials could be functional in organic solvents and non-biological conditions. Although the exact mechanism of particle size control is unclear, previous studies have shown evidence that Oswald ripening growth mechanisms are likely involved^21^.

The conformation of the peptide backbones within the spherical nanostructures was characterized using Fourier transform infrared (FT-IR) spectroscopy. FT-IR spectra displayed bands at 1600, 1500, and 3300 cm^−1^ which, like analogous bands observed for other noncovalent nanomaterials^22^, resemble the amide I, amide II_II_, and amide A bands typical of protein β sheets (Supplementary Fig. 5)^23^. These results suggest that triskelion motifs self-assemble into three-dimensional nanospheres via β-sheet-formation with a trilateral honeycomb-like symmetry^24^.

### Encapsulation in oligotriazole peptide nanospheres

Clathrin triskelion proteins coat lipid vesicles, entrapping relevant molecules for receptor-mediated uptake and cellular trafficking^16^. We investigated whether guest entrapment was feasible in our triskelion peptide spherical nanostructures. The efficient encapsulation ability of the corresponding nanospheres was determined by inclusion of polar fluorophores, such as rhodamine B, in the hydration media.

Fluorescence microscopy indicated efficient entrapment as revealed by the appearance of small fluorescent spherical structures, consistent with dye-loaded nanospheres (Supplementary Fig. 6)^16^. Interestingly, we were able to facilitate the release of the fluorophore by simple acidification, which led to empty nanocages (Supplementary Fig. 7). Our observation may be attributed to the protonation of the tertiary nitrogen center of the tripropargylamine scaffold followed by electrostatic repulsion. Thus, the self-reproducing nanospheres can serve as potential entrapment agents with a simple proton gated release mechanism.

### Autocatalytic formation of oligotriazole peptide

Living cells are catalytic supramolecular assemblies capable of nearly indefinite growth in the presence of appropriate small molecule feedstocks^25^. One of the major challenges in creating abiotic peptide materials that are capable of indefinite growth is the need for mechanisms by which the catalysts responsible for material synthesis can reproduce. Since oligotriazole Cu^1+^ complexes catalyze triazole formation^18^, and have previously shown autocatalytic behavior^18^, we characterized the ability of the self-assembling oligotriazole peptide ligands to self-reproduce via copper-dependent autocatalysis.

**Fig. 3:**
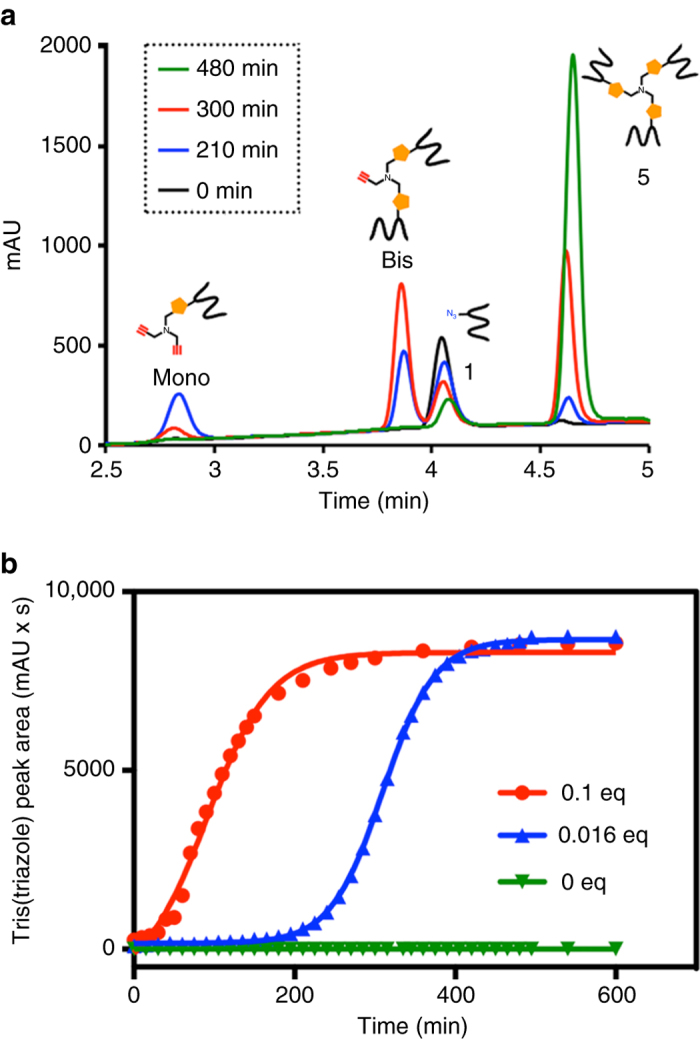
Autocatalytic formation of oligotriazole peptide nanospheres. **a** HPLC traces (210 nm) monitoring the autocatalytic formation of tris(triazole) Boc-*L*-^H^Ala-OH (**5**) by cycloaddition reaction of tripropargylamine with the azidoderivative Boc-*L*-^H^Ala(N_3_)-OH.DCHA (**1**) in the presence of 0.016 equiv. of tris(triazole) **5**. The retention times for all the species were verified by mass spectrometry and the use of known standards. **b** Autocatalytic formation of tris(triazole) peptide **5** in the presence of 0, 0.016, or 0.1 equivalents of tris(triazole) peptide **5**. Sigmoidal growth curves are observed and can be fit to a four-parameter logistic equation (*solid lines*), which are typically used to describe autocatalytic processes. HPLC spectra (210 nm) were used to monitor the progress of the CuAAC reaction. Retention times were verified by mass spectrometry

A difficulty in demonstrating triazole autocatalysis is to differentiate catalysis by hydrated Cu^1+^ ions versus Cu^1+^ bound to oligotriazole ligands. However, previous studies have demonstrated that hydrated Cu^1+^ ions are poor catalysts for forming oligotriazoles in weakly coordinating buffers such as *N*-[tris(hydroxylmethyl)methyl]-2-aminoethanesulfonic acid (TES)^26^. In contrast, addition of oligotriazole peptide ligands allows synthesis of further identical oligotriazole peptides and additional nanospheres. By binding to soluble free Cu^1+^ ions, oligotriazole ligands are capable of catalyzing further triazole formation. Employing high-performance liquid chromatography (HPLC) to monitor the kinetics of oligotriazole peptide synthesis using very low initial oligotriazole **5** starting concentrations (0 equiv., 0.016 equiv., or 0.1 equiv.), we have observed sigmoidal growth curves with an induction period, followed by a rapid exponential rise in ligand formation, inflection, and finally, a plateau as limiting reactant is completely consumed (Fig. 3, Supplementary Figs. 8 and 9). The plateau is reached sooner when higher concentrations of oligotriazole peptide are included to initiate the reaction. Sigmoidal growth curves are hallmarks of autocatalytic processes^27^, which require time for a build up of sufficient catalyst, at which point product conversion sharply accelerates. The pattern of oligotriazole product formation is in sharp contrast to the standard growth curves observed for alternative triazole coupling products^26^. Additional measurements using a wider range of initial oligotriazole concentrations corroborated the autocatalytic effect (Supplementary Fig. 10). We observed that seeding with different equivalents of autocatalyst leads to different kinetic profiles. The greater the initial autocatalyst concentration, the faster a plateau is reached and the limiting reactant is consumed. As the autocatayst amount decreases, the lag phase increases and it takes longer to build up the additional autocatalyst required to eventually consume the reactants. Additionally, the use of a very low concentration of seed catalyst (<0.016 equiv.) leads to a long lag phase before product formation accelerates. Whereas in an ideal autocatalytic system the curves for different concentrations of catalyst should have identical slopes at the same conversion, in our system higher slope values are observed when starting with higher concentrations of catalyst. Such slight deviation from ideal behavior can be a consequence of the complexity of our autocatalytic system, involving multiple reaction steps and the possibility of different active species that are in monomeric or higher order forms. Further studies will be necessary to describe a detailed mechanism for the autocatalytic process, which could shed light on understanding the observed phenomenon. For instance, further data and analysis of the autocatalytic process could be used to calculate the replication order^8, 28^, and suggest some mechanistic features such as the catalyst-substrate stoichometry and the level of product inhibition (if any)^29^. As expected, formation of oligotriazole **5** was not detected over the same observed period when a control in the absence of copper was carried out (Supplementary Fig. 11). Analogous autocatalytic experiments with azidopeptide derivatives (**2**, **3**, or **4**) also show the autocatalytic formation of the corresponding tris(triazole) peptides (**6**, **7**, or **8**) (Supplementary Figs. 12–14), which exhibit rapid self-organization into spherical nanostructures (Supplementary Fig. 15). Additionally, experiments demonstrated that the mono(triazole) or bis(triazole) intermediates do not have significant catalytic ability (Supplementary Fig. 16). Therefore, we strongly believe that the tris(triazoles) are the only species responsible for the autocatalytic behavior. To our knowledge, there is no observed accelerating effect with mono(triazoles) (otherwise all CuAAC “click” reactions would be autocatalytic), and regarding bis(triazoles), there is previously reported evidence that they are unlikely to be as active as tris(triazoles) due to their expected weaker ability to ligate to copper and the potential formation of inhibitory complexes^30, 31^.

### Self-propagation of catalytic oligotriazole peptides

**Fig. 4:**
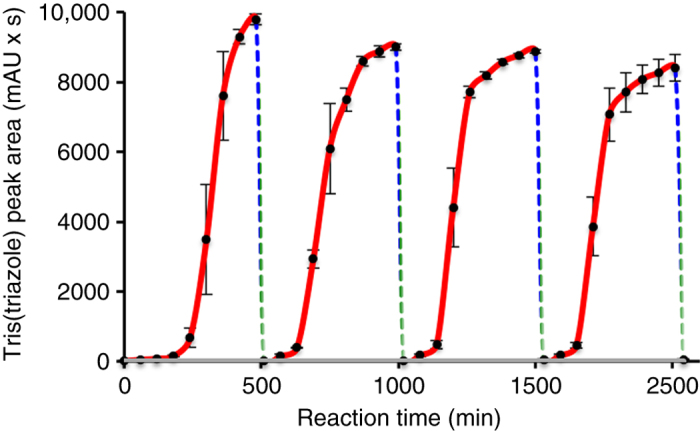
Continual propagation of catalytic peptide nanospheres through serial transfers. Tris(triazole) peptide **5** formation (*red line*) with sequential serial transfers of nanospheres into fresh precursor solutions (*blue*/*green gradated line*) shows repeated and long-term oligotriazole peptide formation relative to controls lacking catalytic peptides (*gray line*). Tris(triazole) formation was monitored over time using combined liquid chromatography (LC), mass spectrometry (MS), and evaporative light-scattering detection (ELSD) measurements (raw LC data at 210 nm shown). Error bars represent standard deviations (SD) (*n* = 3)

To demonstrate continual self-propagation of peptide nanospheres in the presence of appropriate precursors, we conducted serial transfer experiments (Fig. 4, Supplementary Fig. 17). Such experiments show that small species in equilibria and/or the supramolecular nanospheres can continually catalyze tris(triazole) peptide synthesis (Fig. 4). Autocatalytic peptides were combined with a solution containing peptide precursors, as well as a source of Cu^1+^. Over time, the precursors are converted to additional catalytic peptides. After the precursors are depleted, a fraction (1.6%) of the peptide nanosphere population is isolated and combined with a new precursor solution, enabling further peptide formation. Indeed, tris(triazole) peptide formation (*red* line) with sequential serial transfers of nanospheres into fresh precursor solutions (*blue*/*green degraded line*) showed repeated and long-term oligotriazole peptide formation (Fig. 4) relative to controls lacking catalytic peptides (*gray line*). These experiments highlight the importance of the “coupling” between autocatalysis and self-assembly. Such “coupling” is provided by the oligotriazole peptides, which can both self-reproduce and self-assemble. Therefore, the use of autocatalytic molecules that also have the capacity for self-assembly brings the opportunity to continually reproduce self-assembled materials, regardless of the precise mechanism of catalysis (i.e., whether the monomers, self-assembled materials, or both are catalytically active). In an effort to better understand the nature of the catalytically active species, experiments were developed to determine if monomers or small oligomeric species are present after nanosphere formation. Filtrate obtained after spin filtration of nanospheres, using 30 kDa molecular weight cut-off filters, was shown to be catalytically active, indicating that the tris(triazole) building blocks are likely in dynamic equilibrium between the particles and solution (Supplementary Figs. 18 and 19). This rapid dynamic equilibrium makes it exceedingly difficult to pinpoint the catalytic species or the relative catalytic activity of monomers compared to higher ordered structures. Additionally, it is also unclear how the starting materials partition between the spheres and the monomers, and whether this affects the reaction. Therefore, it is likely that free ligands act as catalysts, though it is also feasible that there are catalytically active sites on the nanospheres themselves. We also analyzed the effect of the continued production of tris(triazole) on the nanospheres. DLS measurements over time showed that the self-assembled peptide nanospheres formed from oligotriazole peptide **5** increased their sizes under standard CuAAC autocatalytic conditions (Supplementary Fig. 20). The corresponding growth is probably due to some additional self-assembling interactions. Overall, these results provide strong evidence that the oligotriazole peptide materials, by serving as sources of Cu^1+^ ligands, can autocatalyze the formation of additional ligands, which self-assemble into nanoparticles in the presence of Cu^1+^ and appropriate azide and alkyne precursors.

### Selective formation of oligotriazole peptides

In principle, oligotriazoles can accelerate the synthesis of new triazoles, regardless of the nature of the azide and alkyne precursors. However, we were interested if biased oligotriazole peptide formation takes place in the presence of multiple azide-peptide precursors and a limiting concentration of tripropargylamine scaffold. To test if preferential oligotriazole peptide formation was possible, we incubated peptide nanoparticles consisting of aromatic peptides [tris(triazole) peptide ^H^AlaTrpTrp **7**; final concentration: 10.0 µM] along with tripropargylamine (final concentration: 1 mM) and an equimolar mixture of aromatic azide-modified peptides (^H^Ala(N_3_)TrpTrp **3**; final concentration: 3.3 mM and non-aromatic azide-modified peptides (^H^Ala(N_3_)AlaAla **4**; final concentration: 3.3 mM). If no bias exists during reaction, we would expect a 1:3:3:1 ratio of new tripodal oligotriazoles, with the majority of newly formed ligands having a mixture of aromatic and non-aromatic peptide arms. Instead we observed a strong preference for the formation of oligotriazole **7** from the aromatic peptide precursors (Supplementary Fig. 21). Some autocatalytic reactions can be preferred over others for a wide variety of reasons. In our particular scenario, we believe that partitioning and tryptophan pi-stacking interactions are the main reasons for the observed selectivity, especially if we consider that the inherent reactivities of the azide-modified peptides **3** and **4** to form the corresponding oligotriazole peptides **7** and **8** are both similar (Supplementary Figs. 13 and 14).

## Discussion

The CuAAC-based methodology represents a powerful tool for the autocatalytic formation of self-assembling synthetic tris(triazole) peptide conjugates, which exhibit rapid self-organization into spherical nanosized structures. There is tremendous interest in coupling autocatalytic molecules to supramolecular material self-assembly. Building on initial studies of self-replicating peptides in solution, there have been limited examples of β-sheet forming peptide-based structures that can self-reproduce in a template dependent manner^10, 11^. Despite these previous pioneering studies, there are relatively few demonstrations of self-replicating peptide-based nanomaterials and, to date, most studies have been limited to the reproduction of two-dimensional tubes or fibers. Furthermore, template based methods of reproduction are selective, but can be prone to inhibition and are limited in the kinds of peptides and structures that can be effectively reproduced.

We have demonstrated one possible solution to extend the diversity of peptide nanomaterials that can undergo self-reproduction by coupling the ability of peptides to form diverse nanostructures with a highly efficient autocatalytic process. We have recently discovered that tripodal oligotriazole ligands can self-reproduce by binding to Cu^1+^ ions and activating the catalysis of azide and alkyne precursors to form additional oligotriazole ligands^26^. Oligotriazole synthesis therefore displays the features of an autocatalytic reaction. Inspired by the vast precedent of oligopeptides that self assemble into different materials (spheres, tubes, gels)^3–8^ and the ability to use peptide sequence to control material properties, we chose to examine if oligotriazole peptides could assemble into well-defined materials that can self-reproduce.

Our results provide strong evidence that the oligotriazole peptides, by serving as Cu^1+^ ligands, can autocatalyze the formation of additional oligotriazoles in the presence of Cu^1+^ and appropriate azide and alkyne precursors. These oligotriazoles can self-assemble spontaneously into peptide-based nanospheres. We also observed differential reactivity of azide precursors during oligotriazole synthesis. The origin of the observed reaction preference could be due to aromatic pi-stacking effects, which help associate the peptides into nanospheres. Materials that preceded life may have also behaved similarly, and previous work using computational models have hypothesized that catalytic assemblies can exhibit a fair degree of homeostasis and can be thought of as primitive “compositional genomes”^32^.

Our robust catalytic system is capable of generating functional oligotriazole peptides capable of self-assembling into materials. Combining self-assembling oligotriazoles with triazole autocatalysis may be a promising general strategy for achieving nanomaterial self-reproduction. We believe the diversity of materials linked to self-reproduction can be greatly expanded by altering the structure of the peptide-triazole precursors. Our described approach could lead to the development of novel self-propagating materials based on peptide architectures.

## Methods

### Transmission electron microscopy measurements

Copper grids (formvar/carbon-coated, 400 mesh copper) were prepared by glow discharging the surface at 20 mA for 1.5 min. Once the surface for vesicle adhesion is ready, 3.5 μL of a 5 mM solution of tris(triazole) peptide (**5**, **6**, **7**, or **8**) in H_2_O or organic-aqueous solution (previously tumbled at rt for 24 h) were deposited on the grid surface. This solution was allowed to sit for 10 s before being washed away with 10 drops of glass distilled H_2_O and subsequent staining with 3 drops of 1% w/w uranyl acetate. The stain was allowed to sit for 10 s before wicking away with filter paper. All grid treatments and simple depositions were on the dark/shiny/glossy formvar-coated face of the grid (this side face up during glow discharge). Samples were then imaged via TEM, revealing the presence of several populations of spherical compartments (50–950 nm in diameter), consistent with the vesicle architecture.

### Dynamic light-scattering measurements

10.0 μL of a 10 mM solution of tris(triazole) peptide (**5**, **7**, or **8**) in MeOH were added to a 1 mL vial, placed under N_2_, and dried for 15 min to prepare a peptide film. Then, 100 µL of H_2_O were added, and the solution was tumbled at 25 °C for 24 h. The peptide-containing solutions were finally analyzed by DLS, which corroborated the nanostructure sizes.

### Autocatalytic formation of oligotriazole peptide nanospheres

Representative procedure *for the autocatalytic formation of tris(triazole) Boc-L-* ^*H*^ *Ala-OH* (**5**) [0.016 equiv. of tris(triazole) peptide **5**]: 187 µL of tripropargylamine (20 mM solution in 150 mM TES buffer pH 8.5 in H_2_O; final concentration: 5 mM), 187 µL of Boc-L-^H^Ala(N_3_)-OH.DCHA (**1**, 66 mM solution in 150 mM TES buffer pH 8.5 in H_2_O; final concentration: 16.5 mM), and 2 µL of tris(triazole) Boc-L-^H^Ala-OH (**5**, 30 mM solution in 150 mM TES buffer pH 8.5 in H_2_O; final concentration: 80 µM) were added to a 2 mL vial and stirred for 5 min at rt. Then, we added 187 µL of (+)-sodium L-ascorbate (20 mM solution in 150 mM TES buffer pH 8.5 in H_2_O; final concentration: 5 mM). Finally, 187 µL of CuSO_4_.5H_2_O (10 mM solution in 150 mM TES buffer pH 8.5 in H_2_O; final concentration: 2.5 mM) were added. The heterogeneous mixture was stirred vigorously at rt under N_2_. Periodically, 5 µL aliquots were removed and used directly to monitor the progress of the CuAAC reaction by LC–ELSD–MS.

### Continual synthesis of catalytic oligotriazole nanospheres

Representative procedure *for the continual synthesis of tris(triazole) Boc-L-* ^*H*^ *Ala-OH* (**5**) [1.6% transfer; 0.016 equiv. of tris(triazole) peptide **5**]: 184.5 µL of tripropargylamine (20.32 mM solution in 150 mM TES buffer pH 8.5 in H_2_O; final concentration: 5 mM), 184.5 µL of Boc-*L*-^H^Ala(N_3_)-OH.DCHA (**1**, 67.07 mM solution in 150 mM TES buffer pH 8.5 in H_2_O; final concentration: 16.5 mM), and 12 µL of tris(triazole) Boc-*L*-^H^Ala-OH (**5**, 5 mM solution in 150 mM TES buffer pH 8.5 in H_2_O; final concentration: 80 µM) were added to a 2 mL vial and stirred for 5 min at rt. Then, we added 184.5 µL of (+)-sodium *L*-ascorbate (20.32 mM solution in 150 mM TES buffer pH 8.5 in H_2_O; final concentration: 5 mM). Finally, 184.5 µL of CuSO_4_.5H_2_O (10.16 mM solution in 150 mM TES buffer pH 8.5 in H_2_O; final concentration: 2.5 mM) were added. The heterogeneous mixture was stirred vigorously at rt under N_2_. Periodically, 5 µL aliquots were removed and used directly to monitor the progress of the CuAAC reaction by LC-ELSD-MS. Once the precursors were depleted (≈480 min), a fraction (1.6%; 12 µL) of the nanosphere population wass isolated and combined (≈510 min) with a fresh precursor solution [184.5 µL of tripropargylamine (20.32 mM solution in 150 mM TES buffer pH 8.5 in H_2_O; final concentration: 5 mM), 184.5 µL of Boc-*L*-^H^Ala(N_3_)-OH.DCHA (**1**, 67.07 mM solution in 150 mM TES buffer pH 8.5 in H_2_O; final concentration: 16.5 mM), 184.5 µL of (+)-sodium *L*-ascorbate (20.32 mM solution in 150 mM TES buffer pH 8.5 in H_2_O; final concentration: 5 mM) and 184.5 µL of CuSO_4_.5H_2_O (10.16 mM solution in 150 mM TES buffer pH 8.5 in H_2_O; final concentration: 2.5 mM)]. Subsequent transfers (×3) and additions (×3) were made. Periodically, 5 µL aliquots were removed and used directly to monitor the progress of the CuAAC reaction by LC–ELSD–MS.

### Data availability

The authors declare that the data supporting the findings of this study are available within the paper and its Supplementary Information files, and also are available from the corresponding author upon reasonable request.

## Electronic supplementary material

Supplementary Information
