# Supplementary material for: Continual reproduction of self-assembling oligotriazole peptide nanomaterials
Source: Nat Commun. 2017 Sep 28;8:730. doi: 10.1038/s41467-017-00849-1 (PMC5620040; doi:10.1038/s41467-017-00849-1)
Supplement: Supplementary file 1 — Supplementary Information [file 41467_2017_849_MOESM1_ESM.pdf]

Supplementary Methods

General Considerations

Commercially available N-Boc amino acids, N-Boc-4-azido-*L*-homoalanine (dicyclohexylammonium salt) [Boc-*L*-^{H}Ala(N_3)-OH.DCHA, **1**], *N*-(3-dimethylamino-propyl)-*N'*-ethylcarbodiimide hydrochloride (EDC.HCl), 9-fluorenemethanol (FmOH), 4-dimethylaminopyridine (DMAP), trifluoroacetic acid (TFA), *N*,*N*-diisopropylethylamine (DIEA), O-(7-azabenzotriazol-1-yl)-1,1,3,3-tetramethyl-uronium hexafluorophosphate (HATU), 8-hydroxypyrene-1,3,6-trisulfonic acid (HPTS), rhodamine B, tripropargylamine, copper(II) sulfate pentahydrate ($CuSO_4.5H_2O$), *(+)*-sodium *L*-ascorbate, and *N*-[tris(hydroxylmethyl)methyl]-2-aminoethanesulfonic acid (TES), sodium salt were obtained from Sigma-Aldrich. Deuterated chloroform ($CDCl_3$), methanol (CD_3OD) and water (D_2O) were obtained from Cambridge Isotope Laboratories. All reagents obtained from commercial suppliers were used without further purification unless otherwise noted. Analytical thin-layer chromatography was performed on E. Merck silica gel 60 F_{254} plates. Compounds, which were not UV active, were visualized by dipping the plates in a nynhidrin solution and heating. Silica gel flash chromatography was performed using E. Merck silica gel (type 60SDS, 230-400 mesh). Solvent mixtures for chromatography are reported as v/v ratios. HPLC analysis was carried out on an Eclipse Plus C8 analytical column with *Phase A*/*Phase B* gradients [*Phase A*: H_2O with 0.1% formic acid; *Phase B*: MeOH with 0.1% formic acid]. HPLC purification was carried out on Zorbax SB-C18 semipreparative column with *Phase A*/*Phase B* gradients [*Phase A*: H_2O with 0.1% formic acid; *Phase B*: MeOH with 0.1% formic acid]. Proton nuclear magnetic resonance (^{1}H NMR) spectra were recorded on a Varian VX-500 MHz or Jeol Delta ECA-500 MHz spectrometers, and were referenced relative to residual proton resonances in $CDCl_3$ (at 7.24 ppm) or CD_3OD (at 4.87 or 3.31 ppm). Chemical shifts were reported in parts per million (ppm, δ) relative to tetramethylsilane (δ 0.00). ^{1}H NMR splitting patterns are assigned as singlet (s), doublet (d), triplet (t), quartet (q) or pentuplet (p). All first-order splitting patterns were designated on the basis of the appearance of the multiplet. Splitting patterns that could not be readily interpreted are designated as multiplet (m) or broad (br). Carbon nuclear magnetic resonance (^{13}C NMR) spectra were recorded on a Varian VX-500 MHz or Jeol Delta ECA-500 MHz spectrometers, and were referenced relative to residual proton resonances in $CDCl_3$ (at 77.23 ppm), or CD_3OD (at 49.15 ppm). Electrospray Ionization-Time of Flight (ESI-TOF) spectra were obtained on an Agilent 6230 Accurate-Mass TOFMS mass spectrometer. Transmission electron microscopy images were recorded on a FEI Tecnai™ Sphera 200 kV microscope equipped with a LaB_6 electron gun, using the standard cryotransfer holders developed by Gatan, Inc. Fourier transform infrared (FTIR) spectra were adquired using a Bruker Tensor 27 spectrometer.

Peptide building-blocks were prepared from N-Boc protected amino acids using standard solution-phase peptide synthesis protocols.[1],[2]

Synthesis of Amino Acids

Boc-*L*-Phe-OFm. A solution of Boc-*L*-Phe-OH (500.0 mg, 1.88 mmol) in dry CH_2Cl_2 (10 mL) was treated with EDC.HCl (432.5 mg, 2.26 mmol), 9-fluorenemethanol (388.8 mg, 1.97 mmol) and DMAP (276.1 mg, 2.26 mmol). After 2 h stirring at rt, the

mixture was washed with HCl (5 %) (3 × 5 mL), NH_4Cl (sat.) (1 × 5 mL) and $NaHCO_3$ (sat.) (2 × 5 mL). The organic layer was dried over Na_2SO_4, concentrated under reduced pressure and the resulting crude material was purified by flash chromatography (5-15% EtOAc in hexanes) to give 679.0 mg of Boc-*L*-Phe-OFm [81%, R_f = 0.42 (25% EtOAc in hexanes), white solid]. ^{1}H NMR ($CDCl_3$, 500.13 MHz): δ 7.75 (dd, J_1 = 3.3 Hz, J_2 = 7.5 Hz, 2H), 7.56-7.47 (dd, J_1 = 7.5 Hz, J_2 = 13.1 Hz, 2H), 7.44-7.35 (m, 2H), 7.33-7.17 (m, 5H), 7.13-7.04 (d, *J* = 6.6 Hz, 2H), 4.96 (d, *J* = 8.2 Hz, 1H), 4.74-4.57 (m, 1H), 4.40 (d, *J* = 6.9 Hz, 2H), 4.13 (t, *J* = 6.9 Hz, 1H), 3.17-2.87 (m, 2H), 1.41 (s, 9H) [Supplementary Fig. 22, *top*]. ^{13}C NMR ($CDCl_3$, 125.77 MHz): δ 172.1, 155.3, 143.7, 143.6, 141.5, 141.5, 136.1, 129.5, 128.1, 127.4, 127.4, 127.3, 125.3, 125.2, 120.3, 120.2, 80.2, 67.3, 54.7, 46.9, 38.5, 28.5. MS (ESI-TOF) [m/z (%)]: 467 ([M +Na]$^+$, 100) [Supplementary Fig. 22, *bottom*]. HRMS (ESI-TOF) calculated for $C_{28}H_{29}NO_4Na$ ([M +Na]$^+$) 466.1989, found 466.1991.

Boc-*L*-Trp-OFm. A solution of Boc-*L*-Trp-OH (250.0 mg, 0.82 mmol) in dry CH_2Cl_2 (5 mL) was treated with EDC.HCl (189.0 mg, 0.99 mmol), 9-fluorenemethanol (170.0 mg, 0.86 mmol) and DMAP (120.4 mg, 0.99 mmol). After 2 h stirring at rt, the mixture was washed with HCl (5 %) (3 × 5 mL), NH_4Cl (sat.) (1 × 5 mL) and $NaHCO_3$ (sat.) (2 × 5 mL). The organic layer was dried over Na_2SO_4, concentrated under reduced pressure and the resulting crude material was purified by flash chromatography (5-25% EtOAc in hexanes) to give 325.0 mg of Boc-*L*-Trp-OFm [82%, R_f = 0.43 (25% EtOAc in hexanes), white foam]. ^{1}H NMR ($CDCl_3$, 500.13 MHz): δ 8.11 (s, 1H), 7.88-7.66 (m, 2H), 7.64-7.45 (m, 2H), 7.44-7.35 (m, 3H), 7.34-7.22 (m, 3H), 7.21-7.07 (m, 2H), 6.88 (s, 1H), 5.13 (d, *J* = 9.2 Hz, 1H), 4.82-4.60 (m, 1H), 4.32 (d, *J* = 6.6 Hz, 2H), 4.00 (t, *J* = 7.1 Hz, 1H), 3.25 (d, *J* = 5.9 Hz, 2H), 1.44 (s, 9H) [Supplementary Fig. 23, *top*]. ^{13}C NMR ($CDCl_3$, 125.77 MHz): δ 172.5, 155.4, 144.5, 143.7, 141.7, 141.4, 136.2, 128.0, 127.8, 127.3, 127.3, 127.3, 125.2, 124.9, 122.9, 122.4, 120.2, 120.2, 119.8, 118.9, 111.4, 110.3, 80.1, 67.3, 54.5, 46.7, 28.5, 28.3 [Supplementary Fig. 23, *bottom*]. MS (ESI-TOF) [m/z (%)]: 505 ([M +Na]$^+$, 100). HRMS (ESI-TOF) calculated for $C_{30}H_{30}N_2O_4Na$ ([M +Na]$^+$) 505.2098, found 505.2098.

Boc-*L*-Ala-OFm. A solution of Boc-*L*-Ala-OH (500.0 mg, 2.64 mmol) in dry CH_2Cl_2 (10 mL) was treated with EDC.HCl (607.9 mg, 3.17 mmol), 9-fluorenemethanol (546.4 mg, 2.77 mmol) and DMAP (387.4 mg, 3.17 mmol). After 2 h stirring at rt, the mixture was washed with HCl (5 %) (3 × 5 mL), NH_4Cl (sat.) (1 × 5 mL) and $NaHCO_3$ (sat.) (2 × 5 mL). The organic layer was dried over Na_2SO_4, concentrated under reduced pressure and the resulting crude material was purified by flash chromatography (5-15% EtOAc in hexanes) to give 747.3 mg of Boc-*L*-Ala-OFm [77%, R_f = 0.64 (40% EtOAc in hexanes), white solid]. ^{1}H NMR ($CDCl_3$, 500.13 MHz): δ 7.75 (dd, J_1 = 2.6 Hz, J_2 = 7.7 Hz, 2H), 7.58 (t, *J* = 7.8 Hz, 2H), 7.43-7.35 (m, 2H), 7.34-7.26 (m, 2H), 5.00 (d, *J* = 5.7 Hz, 1H), 4.53-4.41 (m, 2H), 4.40-4.30 (m, 1H), 4.21 (t, *J* = 6.9 Hz, 1H), 1.43 (s, 9H), 1.35 (d, *J* = 6.9 Hz, 3H) [Supplementary Fig. 24, *top*]. ^{13}C NMR ($CDCl_3$, 125.77 MHz): δ 173.5, 155.3, 152.1, 143.8, 143.6, 141.6, 141.5, 128.1, 127.4, 125.2, 125.2, 120.3, 120.2, 80.1, 67.2, 49.5, 47.1, 28.6, 18.8 [Supplementary Fig. 24, *bottom*]. MS (ESI-TOF) [m/z (%)]: 390 ([M +Na]$^+$, 100), 368 ([M +Na]$^+$, 40). HRMS (ESI-TOF) calculated for $C_{22}H_{25}NO_4Na$ ([M +Na]$^+$) 390.1676, found 390.1675.

Synthesis of Dipeptides

Boc-[*L*-Phe]$_2$-OFm. A solution of Boc-*L*-Phe-OFm (400.0 mg, 0.90 mmol) in 2 mL of TFA/CH_2Cl_2 (1:1) was stirred at rt for 15 min. The solvent was removed and the residue was dried under high vacuum for 3 h. The resulting TFA salt was disolved in dry CH_2Cl_2 (4 mL), after which Boc-*L*-Phe-OH (262.6 mg, 0.99 mmol), HATU (376.4 mg, 0.99 mmol) and DIEA (627.0 μL, 3.60 mmol) were successively added. The mixture was stirred for 1 h at rt and the solution was poured into a separating funnel and washed with HCl (5%) (3 × 2 mL) and $NaHCO_3$ (sat.) (3 × 2 mL). The organic layers were dried over Na_2SO_4 and concentrated under reduced pressure to give a yellow oil, which was purified by flash chromatography (10-25% EtOAc in hexanes) to give 483.7 mg of Boc-[*L*-Phe]$_2$-OFm [91%, R_f = 0.26 (20% EtOAc in hexanes), white foam]. ^{1}H NMR ($CDCl_3$, 500.13 MHz): δ 7.75 (dd, J_1 = 4.3 Hz, J_2 = 7.5 Hz, 2H), 7.54-7.43 (m, 2H), 7.42-7.35 (m, 2H), 7.32-7.24 (m, 4H), 7.23-7.11 (m, 6H), 6.97-6.79 (m, 2H), 6.35-6.19 (m, 1H), 5.03-4.67 (m, 2H), 4.49-4.24 (m, 3H), 4.20-4.00 (m, 1H), 3.17-2.73 (m, 4H), 1.40-1.33 (m, 9H) [Supplementary Fig. 25, *top*]. ^{13}C NMR ($CDCl_3$, 125.77 MHz): δ 171.3 and 171.2, 171.0, 155.5, 143.6, 143.6, 143.5, 141.5, 141.5, 135.7, 135.6, 129.6, 129.5, 129.4, 129.4, 128.9, 128.8, 128.2, 128.2, 128.1, 128.1, 127.4, 127.4, 127.2, 125.2, 125.2, 120.3, 120.3, 80.4, 67.4, 53.6, 53.2, 46.8, 38.2, 38.1, 28.4 [Supplementary Fig. 25, *bottom*]. MS (ESI-TOF) [m/z (%)]: 613 ([M +Na]$^+$, 20), 591 ([MH]$^+$, 100). HRMS (ESI-TOF) calculated for $C_{37}H_{38}N_2O_5Na$ ([M +Na]$^+$) 613.2673, found 613.2675.

Boc-[*L*-Trp]$_2$-OFm. A solution of Boc-*L*-Trp-OFm (200.0 mg, 0.42 mmol) in 1 mL of TFA/CH_2Cl_2 (1:1) was stirred at rt for 15 min. The solvent was removed and the residue was dried under high vacuum for 3 h. The resulting TFA salt was disolved in dry CH_2Cl_2 (2 mL), after which Boc-*L*-Trp-OH (138.9 mg, 0.46 mmol), HATU (173.6 mg, 0.46 mmol) and DIEA (289.1 μL, 1.66 mmol) were successively added. The mixture was stirred for 1 h at rt and the solution was poured into a separating funnel and washed with HCl (5%) (3 × 1 mL) and $NaHCO_3$ (sat.) (2 × 1 mL). The organic layers were dried over Na_2SO_4 and concentrated under reduced pressure to give a yellow oil, which was purified by flash chromatography (10-50% EtOAc in hexanes) to give 207.7 mg of Boc-[*L*-Trp]$_2$-OFm [75%, R_f = 0.60 (50% EtOAc in hexanes), white foam]. ^{1}H NMR ($CDCl_3$, 500.13 MHz): δ 7.89 (br s, 2H), 7.73 (dd, J_1 = 3.9 Hz, J_2 = 7.6 Hz, 2H), 7.62 (d, J_2 = 7.3 Hz, 1H), 7.46-7.33 (m, 4H), 7.29-7.24 (m, 4H), 7.20-7.04 (m, 4H), 6.93-6.81 (m, 2H), 6.51 (br s, 1H), 6.25 (d, J = 6.2 Hz, 1H), 5.10 (d, J = 7.7 Hz, 1H), 4.92-4.79 (m, 1H), 4.54-4.35 (m, 1H), 4.34-4.22 (m, 1H), 4.21-4.13 (m, 1H), 4.04-3.93 (m, 1H), 3.40-3.23 (m, 1H), 3.17-2.99 (m, 3H), 1.37 (s, 9H) [Supplementary Fig. 26, *top*]. ^{13}C NMR ($CDCl_3$, 125.77 MHz): δ 171.5, 171.4, 155.6, 143.6, 143.6, 141.4, 141.4, 136.3, 136.1, 128.0, 127.7, 127.6, 127.4, 125.2, 123.6, 123.0, 122.4, 120.2, 120.2, 120.0, 119.8, 119.2, 118.6, 111.4, 111.3, 110.6, 109.8, 80.2, 67.4, 60.6, 55.3 and 53.2, 46.7, 28.5, 27.9 [Supplementary Fig. 26, *bottom*]. MS (ESI-TOF) [m/z (%)]: 691 ([M +Na]$^+$, 10), 669 ([MH]$^+$, 100). HRMS (ESI-TOF) calculated for $C_{41}H_{40}N_4O_5Na$ ([M +Na]$^+$) 691.2891, found 691.2890.

Boc-[*L*-Ala]$_2$-OFm. A solution of Boc-*L*-Ala-OFm (500.0 mg, 1.36 mmol) in 2 mL of TFA/CH_2Cl_2 (1:1) was stirred at rt for 15 min. The solvent was removed and the residue was dried under high vacuum for 3 h. The resulting TFA salt was disolved in dry CH_2Cl_2 (5 mL), after which Boc-*L*-Ala-OH (283.8 mg, 1.50 mmol), HATU (570.3 mg,

1.50 mmol) and DIEA (947.5 μL, 5.44 mmol) were successively added. The mixture was stirred for 1 h at rt and the solution was poured into a separating funnel and washed with HCl (5%) (3 × 3 mL) and $NaHCO_3$ (sat.) (3 × 3 mL). The organic layers were dried over Na_2SO_4 and concentrated under reduced pressure to give a yellow oil, which was purified by flash chromatography (10-25% EtOAc in hexanes) to give 554.3 mg of Boc-[*L*-Ala]$_2$-OFm [93%, R_f = 0.29 (25% EtOAc in hexanes), white foam]. ^{1}H NMR ($CDCl_3$, 500.13 MHz): δ 7.75 (dd, J_1 = 3.4 Hz, J_2 = 7.5 Hz, 2H), 7.64-7.48 (m, 2H), 7.46-7.34 (m, 2H), 7.34-7.26 (m, 2H), 6.56 (br s, 1H), 4.99 (br s, 1H), 4.63-4.53 (m, 1H), 4.48 (d, *J* = 6.5 Hz, 2H), 4.20 (t, *J* = 6.6 Hz, 1H), 4.18-4.07 (m, 1H), 1.42 (s, 9H), 1.32 (d, *J* = 7.1 Hz, 3H), 1.29 (d, *J* = 7.1 Hz, 3H) [Supplementary Fig. 27, *top*]. ^{13}C NMR ($CDCl_3$, 125.77 MHz): δ 172.8, 172.3, 155.7, 143.7, 143.5, 141.6, 141.5, 128.1, 128.1, 127.4, 125.2, 125.1, 120.3, 120.3, 80.4, 67.2, 50.2, 48.3, 47.0, 28.5, 18.5 [Supplementary Fig. 27, *bottom*]. MS (ESI-TOF) [m/z (%)]:461 ([M +Na]$^+$, 45), 439 ([MH]$^+$, 100). HRMS (ESI-TOF) calculated for $C_{25}H_{30}N_2O_5Na$ ([M +Na]$^+$), 461.2047 found 461.2048.

Synthesis of Tripeptides

Boc-*L*-^{H}Ala(N$_3$)-[*L*-Phe]$_2$-OFm. A solution of Boc-[*L*-Phe]$_2$-OFm (50.0 mg, 84.7 μmol) in 2 mL of TFA/CH_2Cl_2 (1:1) was stirred at rt for 15 min. The solvent was removed and the residue was dried under high vacuum for 3 h. The resulting TFA salt was disolved in dry CH_2Cl_2 (2 mL), after which Boc-*L*-^{H}Ala(N$_3$)-OH.DCHA (**1**, 43.3 mg, 101.7 μmol), HATU (38.7 mg, 101.7 μmol) and DIEA (59.0 μL, 338.8 μmol) were successively added. The mixture was stirred for 1 h at rt and the solution was poured into a separating funnel and washed with HCl (5%) (3 × 1 mL) and $NaHCO_3$ (sat.) (2 × 1 mL). The organic layers were dried over Na_2SO_4 and concentrated under reduced pressure to give a pale yellow foam, which was purified by flash chromatography (0-3% MeOH in CH_2Cl_2) to give 48.9 mg of the desired azidotripeptide [81%, R_f = 0.38 (5% MeOH in CH_2Cl_2), white foam]. ^{1}H NMR ($CDCl_3$, 500.13 MHz): δ 7.80-7.70 (m, 2H), 7.55-7.42 (m, 2H), 7.41-7.34 (m, 2H), 7.33-7.07 (m, 12H), 6.99-6.91 (m, 2H), 5.34-5.19 (m, 1H), 4.91-4.57 (m, 2H), 4.47-4.26 (m, 2H), 4.24-4.02 (m, 2H), 3.50-2.83 (m, 6H), 1.97-1.61 (m, 2H), 1.42 and 1.38 (s, 9H) [Supplementary Fig. 28, *top*]. ^{13}C NMR ($CDCl_3$, 125.77 MHz): δ 171.4, 171.2 and 171.1, 170.6 and 170.3, 155.7, 143.6, 143.5, 141.5, 141.4, 136.3, 135.7, 129.5, 129.4, 129.4, 129.3, 128.9, 128.8, 128.8, 128.8, 128.1, 128.1, 128.0, 127.4, 127.4, 127.3, 127.3, 125.2, 125.1, 120.2, 80.6, 67.4, 54.3, 53.7, 52.6, 47.9, 46.7, 38.8, 38.0, 37.9, 32.1, 28.5 [Supplementary Fig. 28, *bottom*]. MS (ESI-TOF) [m/z (%)]: 739 ([M +Na]$^+$, 100), 717 ([MH]$^+$, 16). HRMS (ESI-TOF) calculated for $C_{41}H_{44}N_6O_6Na$ ([M +Na]$^+$) 739.3215, found 739.3214.

Boc-*L*-^{H}Ala(N$_3$)-[*L*-Phe]$_2$-OH (2). A solution of the tripeptide Boc-*L*-^{H}Ala(N$_3$)-[*L*-Phe]$_2$-OFm (20.0 mg, 27.9 μmol) in 20% piperidine/CH_2Cl_2 (250 μL) was stirred at rt for 20 min and then the solvent was removed *in vacuo*. The residue was dissolved in CH_2Cl_2 (250 μL) and washed with HCl (5 %) (2 × 250 μL), dried over Na_2SO_4, filtered and concentrated to give a white solid. The corresponding residue was dissolved in MeOH (200 μL), filtered using a 0.2 μm syringe-driven filter, and the crude solution was purified by HPLC, affording 9.6 mg of the azidotripeptide **2** as a white solid [64%, t_R = 12.0 min (Zorbax SB-C18 semipreparative column, 60-5% *Phase A* in *Phase B*, 15.5 min)]. ^{1}H NMR (CD_3OD, 500.13 MHz): δ 7.36-7.08 (m, 10H), 4.75-4.50 (m, 2H),

4.23-3.86 (m, 1H), 3.29-3.08 (m, 4H), 3.04-2.95 (m, 1H), 2.92-2.83 (m, 1H), 1.92-1.73 (m, 1H), 1.72-1.59 (m, 1H), 1.43 (s, 9H) [Supplementary Fig. 29, *top*]. ^{13}C NMR (CD_3OD, 125.77 MHz): δ 174.6, 173.9, 173.0, 157.8, 138.4, 138.3, 130.6, 130.5, 129.6, 129.5, 127.9, 127.9, 81.0, 55.6, 55.4, 53.7, 49.2, 39.1, 38.6, 32.5, 28.9 [Supplementary Fig. 29, *bottom*]. MS (ESI-TOF) [m/z (%)]: 561 ([M +Na]$^+$, 15), 539 ([MH]$^+$, 100). HRMS (ESI-TOF) calculated for $C_{27}H_{34}N_6O_6Na$ ([M +Na]$^+$) 561.2432, found 561.2431.

Boc-*L*-$^{\mathbf{H}}$Ala(N$_3$)-[*L*-Trp]$_2$-OFm. A solution of Boc-[*L*-Trp]$_2$-OFm (75.0 mg, 112.0 μmol) in 2 mL of TFA/CH_2Cl_2 (1:1) was stirred at rt for 15 min. The solvent was removed and the residue was dried under high vacuum for 3 h. The resulting TFA salt was disolved in dry CH_2Cl_2 (2 mL), after which Boc-*L*-^{H}Ala(N_3)-OH.DCHA (**1**, 57.2 mg, 134.0 μmol), HATU (51.1 mg, 134.0 μmol) and DIEA (78.0 μL, 448.0 μmol) were successively added. The mixture was stirred for 1 h at rt and the solution was poured into a separating funnel and washed with HCl (5%) (3 × 1 mL) and $NaHCO_3$ (sat.) (2 × 1 mL). The organic layers were dried over Na_2SO_4 and concentrated under reduced pressure to give a pale yellow foam, which was purified by flash chromatography (0-3% MeOH in CH_2Cl_2) to give 83.2 mg of the desired azidotripeptide [93%, R_f = 0.45 (5% MeOH in CH_2Cl_2), pale yellow foam]. ^{1}H NMR ($CDCl_3$, 500.13 MHz): δ 8.31 (s, 1H), 8.24 (s, 1H), 7.80-7.68 (m, 2H), 7.61 (d, *J* = 7.5 Hz, 1H), 7.52-7.41 (m, 2H), 7.40-7.33 (m, 2H), 7.31-7.19 (m, 5H), 7.17-7.02 (m, 4H), 6.94-6.72 (m, 2H), 6.64-6.50 (m, 2H), 5.23 (m, 1H), 4.97-4.66 (m, 2H), 4.42-4.13 (m, 2H), 4.13-3.94 (m, 2H), 3.35-3.23 (m, 1H), 3.20-2.91 (m, 5H), 1.89-1.58 (m, 2H), 1.41 (s, 9H) [Supplementary Fig. 30, *top*]. ^{13}C NMR ($CDCl_3$, 125.77 MHz): δ 171.4, 171.2, 170.9, 155.7, 143.7, 143.6, 141.4, 141.4, 136.3, 136.1, 128.0, 127.6, 127.5, 127.4, 127.3, 125.3, 125.2, 123.9, 123.4, 122.4, 122.2, 120.2, 120.2, 119.9, 119.6, 118.9, 118.4, 111.5, 110.1, 109.4, 80.7, 67.4, 53.9, 53.1, 52.7, 48.0, 46.7, 38.9, 32.2, 32.1, 28.5 [Supplementary Fig. 30, *bottom*]. MS (ESI-TOF) [m/z (%)]: 817 ([M +Na]$^+$, 15), 795 ([MH]$^+$, 100). HRMS (ESI-TOF) calculated for $C_{45}H_{46}N_8O_6Na$ ([M +Na]$^+$) 817.3433, found 817.3428.

Boc-*L*-$^{\mathbf{H}}$Ala(N$_3$)-[*L*-Trp]$_2$-OH (3). A solution of the tripeptide Boc-*L*-^{H}Ala(N_3)-[*L*-Trp]$_2$-OFm (15.0 mg, 18.9 μmol) in 20% piperidine/CH_2Cl_2 (250 μL) was stirred at rt for 20 min and then the solvent was removed *in vacuo*. The residue was dissolved in CH_2Cl_2 (250 μL) and washed with HCl (5 %) (2 × 250 μL), dried over Na_2SO_4, filtered and concentrated to give a white solid. The corresponding residue was dissolved in MeOH (200 μL), filtered using a 0.2 μm syringe-driven filter, and the crude solution was purified by HPLC, affording 8.3 mg of the azidotripeptide **3** as a white solid [88%, t_R = 10.7 min (Zorbax SB-C18 semipreparative column, 60-5% *Phase A* in *Phase B*, 15.5 min)]. ^{1}H NMR (CD_3OD, 500.13 MHz): δ 7.58 (d, *J* = 7.8 Hz, 1H), 7.38-7.26 (m, 3H), 7.16-6.90 (m, 6H), 4.78-4.54 (m, 2H), 4.09-3.81 (m, 1H), 3.29-3.01 (m, 6H), 1.80-1.65 (m, 1H), 1.61-1.49 (m, 1H), 1.40 (s, 9H) [Supplementary Fig. 31, *top*]. ^{13}C NMR (CD_3OD, 125.77 MHz): δ 175.4, 174.0, 173.4, 157.8, 138.2, 138.0, 129.1, 129.0, 125.0, 124.8, 122.6, 122.5, 120.0, 120.0, 119.6, 119.5, 112.5, 112.4, 110.8, 110.7, 81.0, 55.2, 53.8, 49.4, 49.1, 32.2, 28.8, 28.5 [Supplementary Fig. 31, *bottom*]. MS (ESI-TOF) [m/z (%)]: 639 ([M +Na]$^+$, 100), 617 ([MH]$^+$, 35). HRMS (ESI-TOF) calculated for $C_{31}H_{36}N_8O_6Na$ ([M +Na]$^+$) 639.2650, found 639.2645.

Boc-*L*-$^{\mathbf{H}}$Ala(N$_3$)-[*L*-Ala]$_2$-OFm. A solution of Boc-[*L*-Ala]$_2$-OFm (50.2 mg, 114.6 μmol) in 1 mL of TFA/CH_2Cl_2 (1:1) was stirred at rt for 15 min. The solvent was

removed and the residue was dried under high vacuum for 3 h. The resulting TFA salt was disolved in dry CH_2Cl_2 (1 mL), after which Boc-*L*-^{H}Ala(N_3)-OH.DCHA (**1**, 48.8 mg, 114.6 μmol), HATU (48.0 mg, 126.0 μmol) and DIEA (79.9 μL, 458.2 μmol) were successively added. The mixture was stirred for 1 h at rt and the solution was poured into a separating funnel and washed with HCl (5%) (2 × 1 mL) and $NaHCO_3$ (sat.) (1 × 1 mL). The organic layers were dried over Na_2SO_4 and concentrated under reduced pressure to give a yellow oil, which was purified by flash chromatography (0-5% MeOH in CH_2Cl_2) to give 56.7 mg of the desired azidotripeptide [88%, R_f = 0.48 (5% MeOH in CH_2Cl_2), white solid]. ^{1}H NMR ($CDCl_3$, 500.13 MHz): δ 7.74 (dd, J_1 = 3.6 Hz, J_2 = 7.5 Hz, 2H), 7.63-7.47 (m, 2H), 7.43-7.34 (m, 2H), 7.33-7.25 (m, 2H), 6.88 (br s, 1H), 6.76 (br s, 1H), 5.33 (br s, 1H), 4.60-4.51 (m, 1H), 4.50-4.42 (m, 3H), 4.30-4.22 (m, 1H), 4.20 (t, *J* = 6.7 Hz , 1H), 3.41 (t, *J* = 6.4 Hz, 2H), 2.11-1.95 (m, 1H), 1.94-1.81 (m, 1H), 1.42 (s, 9H), 1.34 (d, *J* = 7.4 Hz, 3H), 1.28 (d, *J* = 7.2 Hz, 3H) [Supplementary Fig. 32, *top*]. ^{13}C NMR ($CDCl_3$, 125.77 MHz): δ 172.7, 171.6, 171.3, 155.8, 143.7, 143.5, 141.6, 141.5, 128.1, 128.1, 127.4, 127.4, 125.1, 125.1, 120.3, 120.2, 80.7, 67.2, 52.3, 48.4, 48.4, 47.0, 38.8, 31.9, 28.5, 18.5, 18.3 [Supplementary Fig. 32, *bottom*]. MS (ESI-TOF) [m/z (%)]: 587 ([M +Na]$^+$, 5), 565 ([MH]$^+$, 100). HRMS (ESI-TOF) calculated for $C_{29}H_{37}N_6O_6$ ([MH]$^+$) 565.2769, found 565.2765.

Boc-*L*-^{H}Ala(N_3)-[*L*-Ala]$_2$-OH (4). A solution of the tripeptide Boc-*L*-^{H}Ala(N_3)-[*L*-Ala]$_2$-OFm (30.0 mg, 53.2 μmol) in 20% piperidine/CH_2Cl_2 (250 μL) was stirred at rt for 20 min and then the solvent was removed *in vacuo*. The residue was dissolved in CH_2Cl_2 (250 μL) and washed with HCl (5 %) (2 × 250 μL), dried over Na_2SO_4, filtered and concentrated to give a white solid. The corresponding residue was dissolved in MeOH (200 μL), filtered using a 0.2 μm syringe-driven filter, and the crude solution was purified by HPLC, affording 12.1 mg of the azidotripeptide **4** as a colorless film [59%, t_R = 10.5 min (Zorbax SB-C18 semipreparative column, 60-5% *Phase A* in *Phase B*, 15.5 min)]. ^{1}H NMR (CD_3OD, 500.13 MHz): δ 4.46-4.27 (m, 2H), 4.23-4.07 (m, 1H), 3.54-3.36 (m, 2H), 2.11-1.92 (m, 1H), 1.91-1.74 (m, 1H), 1.45 (s, 9H), 1.39 (d, *J* = 7.3 Hz, 3H), 1.37 (d, *J* = 6.8 Hz, 3H) [Supplementary Fig. 33, *top*]. ^{13}C NMR (CD_3OD, 125.77 MHz): δ 174.8, 172.9, 172.6, 156.4, 79.4, 52.0, 48.8, 48.4, 48.2, 31.0, 27.3, 16.7, 16.4 [Supplementary Fig. 33, *bottom*]. MS (ESI-TOF) [m/z (%)]: 409 ([M +Na]$^+$, 10), 387 ([MH]$^+$, 100). HRMS (ESI-TOF) calculated for $C_{15}H_{26}N_6O_6Na$ ([M +Na]$^+$) 409.1806, found 409.1807.

Synthesis of Tris(triazole) Peptides

- *Copper catalyzed azide-alkyne cycloaddition (CuAAC) methodology*

Mono(triazole) Boc-*L*-^{H}Ala-OH (Mono). Representative procedure for the synthesis of mono(triazole) peptides. A solution of tripropargylamine (5.0 mg, 38.1 μmol) and Boc-*L*-^{H}Ala(N_3)-OH.DCHA (**1**, 17.8 mg, 41.9 μmol) in EtOH (375 μL) was stirred for 5 min ar rt. Then, a preactivated solution of $CuSO_4.5H_2O$ (0.91 mg, 5.72 μmol) and *(+)*-sodium *L*-ascorbate (2.27 mg, 11.4 μmol) in H_2O (375 μL) was added. The heterogeneous mixture was stirred vigorously at rt under N_2 for 5 h. Afterwards, the corresponding yellow solution was filtered using a 0.2 μm syringe-driven filter. The crude solution was then purified by HPLC, affording 8.3 mg of the desired mono(triazole) peptide **Mono** as a colorless film [58%, t_R = 8.8 min (Zorbax SB-C18 semipreparative column, 60-5% *Phase A* in *Phase B*, 18.5 min)], as well as 6.8 mg of the

bis(triazole) peptide **Bis** [29%] and 1.9 mg of the tris(triazole) peptide **5** [6%]. ^{1}H NMR (CD_3OD, 500.13 MHz): δ 8.09 (s, 1H), 4.82-4.38 (m, 3H), 4.26-3.74 (m, 4H), 3.36-3.32 (m, 1H), 3.30-3.26 (m, 1H), 2.82 (s, 2H), 2.70-2.15 (m, 2H), 1.46 (s, 9H) [Supplementary Fig. 34, *top*]. ^{13}C NMR (CD_3OD, 125.77 MHz): δ 162.8, 158.2, 130.7, 129.0, 81.7, 80.7, 77.0, 49.6, 49.3, 32.5, 30.6, 28.7, 28.6 [Supplementary Fig. 34, *bottom*]. MS (ESI-TOF) [m/z (%)]: 376 ($[MH]^+$, 100). HRMS (ESI-TOF) calculated for $C_{18}H_{26}N_5O_4$ ($[MH]^+$) 376.1979, found 376.1981.

Bis(triazole) Boc-*L*-^{H}Ala-OH (Bis). Representative procedure for the synthesis of bis(triazole) peptides. A solution of tripropargylamine (5.0 mg, 38.1 μmol) and Boc-*L*-^{H}Ala(N_3)-OH.DCHA (**1**, 35.7 mg, 83.9 μmol) in EtOH (375 μL) was stirred for 5 min ar rt. Then, a preactivated solution of $CuSO_4.5H_2O$ (0.91 mg, 5.72 μmol) and *(+)*-sodium *L*-ascorbate (2.27 mg, 11.4 μmol) in H_2O (375 μL) was added. The heterogeneous mixture was stirred vigorously at rt under N_2 for 5 h. Afterwards, the corresponding yellow solution was filtered using a 0.2 μm syringe-driven filter. The crude solution was then purified by HPLC, affording 12.0 mg of the desired bis(triazole) peptide **Bis** as a colorless film [51%, t_R = 10.3 min (Zorbax SB-C18 semipreparative column, 60-5% *Phase A* in *Phase B*, 18.5 min)], as well as 2.9 mg of the mono(triazole) peptide **Mono** [20%] and 7.7 mg of the tris(triazole) peptide **5** [23%]. ^{1}H NMR (CD_3OD, 500.13 MHz): δ 8.18-7.85 (m, 2H), 4.72-4.41 (m, 4H), 4.16-3.78 (m, 6H), 3.56-3-37 (m, 2H), 2.81 (s, 1H), 2.64-2.38 (m, 2H), 2.36-2.11 (m, 2H), 1.45 (s, 18H) [Supplementary Fig. 35, *top*]. ^{13}C NMR (CD_3OD, 125.77 MHz): δ 175.0, 158.1, 144.4, 126.2, 80.8, 78.1, 76.4, 53.6, 52.3, 42.7, 33.1, 28.7, 28.6 [Supplementary Fig. 35, *bottom*]. MS (ESI-TOF) [m/z (%)]: 620 ($[MH]^+$, 100). HRMS (ESI-TOF) calculated for $C_{22}H_{42}N_9O_8$ ($[MH]^+$) 620.3151, found 620.3149.

Tris(triazole) Boc-*L*-^{H}Ala-OH (5). Representative procedure for the synthesis of tris(triazole) peptides. A solution of tripropargylamine (10.0 mg, 76.2 μmol) and Boc-*L*-^{H}Ala(N_3)-OH.DCHA (**1**, 107.1 mg, 251.6 μmol) in EtOH (750μL) was stirred for 5 min ar rt. Then, a preactivated solution of $CuSO_4.5H_2O$ (1.83 mg, 11.43 μmol) and *(+)*-sodium *L*-ascorbate (4.53 mg, 22.9 μmol) in H_2O (750 μL) was added. The heterogeneous mixture was stirred vigorously at rt under N_2 for 24 h. Afterwards, the corresponding yellow solution was filtered using a 0.2 μm syringe-driven filter. The crude solution was then purified by HPLC, affording 83.7 mg of the tris(triazole) peptide **5** as a white foam [78%, t_R = 9.6 min (Zorbax SB-C18 semipreparative column, 60-5% *Phase A* in *Phase B*, 18.5 min)]. ^{1}H NMR (CD_3OD, 500.13 MHz): δ 8.39 (s, 3H), 4.88-4.37 (m, 12H), 4.19-3.86 (m, 3H), 2.74-2.42 (m, 3H), 2.40-2.13 (m, 3H), 1.42 (s, 27H) [Supplementary Fig. 36, *top*]. ^{13}C NMR (CD_3OD, 125.77 MHz): δ 175.1, 158.2, 137.6, 129.3, 81.0, 55.3, 52.1, 47.6, 33.1, 28.8, 28.7 [Supplementary Fig. 36, *bottom*]. MS (ESI-TOF) [m/z (%)]: 864 ($[MH]^+$, 100). HRMS (ESI-TOF) calculated for $C_{36}H_{58}N_{13}O_{12}$ ($[MH]^+$) 864.4322, found 864.4325.

Tris(triazole) Boc-*L*-^{H}Ala-[*L*-Phe]$_2$-OH (6). Using the conditions described for **5**, 100.9 mg of tris(triazole) peptide **6** were obtained from Boc-*L*-^{H}Ala(N_3)-[*L*-Phe]$_2$-OH (**2**) [76%, t_R = 18.0 min (Zorbax SB-C18 semipreparative column, 60-5% *Phase A* in *Phase B*, 18.5 min)]. ^{1}H NMR (CD_3OD, 500.13 MHz): δ 7.98 (s, 3H), 7.24-7.16 (m, 25H), 7.16-7.08 (m, 5H), 4.72-4.54 (m, 6H), 4.42-4.21 (m, 6H), 4.07-3.92 (m, 3H), 3.84 (s, 6H), 3.47-3.05 (m, 6H), 3.04-2.94 (m, 3H), 2.90-2.79 (m, 3H), 2.26-2.11 (m, 3H), 2.10-1.96 (m, 3H), 1.41 (s, 27H) [Supplementary Fig. 38, *top*]. ^{13}C NMR (CD_3OD,

125.77 MHz): δ 174.9, 173.5, 173.1, 157.7, 144.9, 138.6, 138.4, 130.6, 130.6, 129.6, 129.6, 127.8, 126.3, 81.0, 55.9, 55.7, 53.3, 48.5, 48.2, 39.1, 38.6, 33.9, 28.9 [Supplementary Fig. 38, *bottom*]. MS (ESI-TOF) [m/z (%)]: 885 ([M +Na]$^{2+}$, 35), 874 ([MH]$^{2+}$, 100). HRMS (ESI-TOF) calculated for $C_{90}H_{111}N_{19}O_{18}Na$ ([M +Na]$^{+}$) 1768.8247, found 1768.8245.

Tris(triazole) Boc-*L*-^{H}Ala-[*L*-Trp]$_2$-OH (7). Using the conditions described for **5**, 111.2 mg of tris(triazole) peptide **7** were obtained from Boc-*L*-^{H}Ala(N_3)-[*L*-Trp]$_2$-OH (**3**) [74%, t_R = 17.0 min (Zorbax SB-C18 semipreparative column, 60-5% *Phase A* in *Phase B*, 18.5 min)]. ^{1}H NMR (CD_3OD, 500.13 MHz): δ 7.75 (s, 3H), 7.55 (d, *J* = 8.0 Hz, 3H), 7.40-7.30 (m, 3H), 7.27 (d, *J* = 7.0 Hz, 3H), 7.22 (d, *J* = 7.2 Hz, 3H), 7.08 (s, 3H), 7.03 (t, *J* = 6.4 Hz, 3H), 7.01-6.92 (m, 9H), 6.86 (t, *J* = 8.0 Hz, 3H), 4.74-4.64 (m, 3H), 4.63-4.54 (m, 3H), 4.19-4.05 (m, 3H), 4.04-3.94 (m, 3H), 3.94-3.82 (m, 3H), 3.71 (s, 6H), 3.47-3.13 (m, 9H), 3.13-3.00 (m, 3H), 2.15-1.95 (m, 3H), 1.91-1.74 (m, 3H), 1.37 (s, 27H) [Supplementary Fig. 40, *top*]. ^{13}C NMR (CD_3OD, 125.77 MHz): δ 173.6, 173.2, 169.7, 157.7, 145.0, 138.1, 137.9, 129.4, 129.0, 126.2, 124.9, 124.8, 122.5, 122.3, 120.0, 119.9, 119.8, 119.6, 112.4, 112.3, 111.4, 110.8, 81.0, 56.3, 55.6, 53.4, 48.9, 47.8, 33.5, 31.0, 28.9, 28.7 [Supplementary Fig. 40, *bottom*]. MS (ESI-TOF) [m/z (%)]: 1013 ([M +Na_2]$^{2+}$, 25), 1002 ([M +Na]$^{2+}$, 80), 991 ([MH]$^{2+}$, 100). HRMS (ESI-TOF) calculated for $C_{102}H_{117}N_{25}O_{18}Na_2$ ([M +Na_2]$^{2+}$) 1012.9396, found 1012.9391.

Tris(triazole) Boc-*L*-^{H}Ala-[*L*-Ala]$_2$-OH (8). Using the conditions described for **5**, 77.6 mg of tris(triazole) peptide **8** were obtained from Boc-*L*-^{H}Ala(N_3)-[*L*-Ala]$_2$-OH (**4**) [77%, t_R = 12.7 min (Zorbax SB-C18 semipreparative column, 60-5% *Phase A* in *Phase B*, 18.5 min)]. ^{1}H NMR (CD_3OD, 500.13 MHz): δ 8.07 (s, 3H), 4.61-4.45 (m, 6H), 4.44-4.34 (m, 3H), 4.33-4.24 (m, 3H), 4.16-3.97 (m, 3H), 3.81 (s, 6H), 2.51-2.29 (m, 3H), 2.28-2.07 (m, 3H), 1.43 (s, 27H), 1.38 (d, *J* = 7.2 Hz, 18H) [Supplementary Fig. 42, *top*]. ^{13}C NMR (CD_3OD, 125.77 MHz): δ 176.8, 174.5, 173.7, 157.8, 145.2, 126.2, 81.0, 53.3, 50.6, 50.0, 49.8, 48.5, 33.8, 28.9, 18.3, 18.2 [Supplementary Fig. 42, *bottom*]. MS (ESI-TOF) [m/z (%)]: 657 ([M +Na]$^{2+}$, 45), 646 ([MH]$^{2+}$, 100). HRMS (ESI-TOF) calculated for $C_{54}H_{87}N_{19}O_{18}Na$ ([M +Na]$^{+}$) 1312.6369, found 1312.6352.

Solution-phase peptide synthetic methodology

Tris(triazole) Boc-*L*-^{H}Ala-[*L*-Phe]$_2$-OFm. A solution of Boc-[*L*-Phe]$_2$-OFm (20.8 mg, 35.2 μmol) in 1 mL of TFA/CH_2Cl_2 (1:1) was stirred at rt for 15 min. The solvent was removed and the residue was dried under high vacuum for 3 h. The resulting TFA salt was disolved in dry CH_2Cl_2 (500 μL), after which tris(triazole) Boc-*L*-^{H}Ala-OH (**5**, 9.2 mg, 10.7 μmol), HATU (13.4 mg, 35.2 μmol) and DIEA (22.3 μL, 128.0 μmol) were successively added. The mixture was stirred for 1 h at rt and the solution was poured into a separating funnel and washed with HCl (5%) (3 × 500 μL) and $NaHCO_3$ (sat.) (2 × 500 μL). The organic layers were dried over Na_2SO_4 and concentrated under reduced pressure to give a pale yellow solid, which was purified by flash chromatography (0-5% MeOH in CH_2Cl_2) to give 17.9 mg of the desired tris(triazole) peptide [74%, R_f = 0.31 (5% MeOH in CH_2Cl_2), white solid]. ^{1}H NMR ($CDCl_3$, 500.13 MHz): δ 7.86-7.59 (m, 6H), 7.58-7.26 (m, 15H), 7.23-6.58 (m, 42H), 5.49-5.23 (m, 3H), 4.92-4.49 (m, 6H), 4.46-3.55 (m, 24H), 3.18-2.71 (m, 12H), 2.41-1.76 (m, 6H), 1.36 (s, 27H) [Supplementary Fig. 37, *top*]. ^{13}C NMR ($CDCl_3$, 125.77 MHz): δ 171.3, 171.1, 170.8, 155.7, 143.7, 143.6, 143.5, 143.5, 141.4, 141.4,

141.4, 136.7, 136.0, 129.5, 129.5, 129.5, 129.4, 129.4, 128.8, 128.7, 128.7, 128.1, 128.1, 128.1, 127.3, 127.3, 127.1, 80.5, 67.4, 53.8, 53.7, 51.3, 46.7, 37.9, 37.5, 32.1, 30.4, 29.9, 28.5 [Supplementary Fig. 37, *bottom*]. MS (ESI-TOF) [m/z (%)]: 1152 ([M +Na]$^{2+}$, 30), 1141 ([MH]$^{2+}$, 100). HRMS (ESI-TOF) calculated for $C_{132}H_{141}N_{19}O_{18}Na_2$ ([M +Na$_2$]$^{2+}$) 1163.2043, found 1163.0249.

Tris(triazole) Boc-*L*-^{H}Ala-[*L*-Phe]$_2$-OH (6). A solution of the tris(triazole) Boc-*L*-^{H}Ala-[*L*-Phe]$_2$-OFm (20.0 mg, 8.8 μmol) in 20% piperidine/CH_2Cl_2 (250 μL) was stirred at rt for 20 min and then the solvent was removed *in vacuo*. The residue was dissolved in CH_2Cl_2 (250 μL) and washed with HCl (5 %) (2 × 250 μL), dried over Na_2SO_4, filtered and concentrated to give a white solid. The corresponding residue was dissolved in MeOH (200 μL), filtered using a 0.2 μm syringe-driven filter, and the crude solution was purified by HPLC, affording 11.8 mg of the tris(triazole) peptide **6** as a white solid [77%, t_R = 18.0 min (Zorbax SB-C18 semipreparative column, 60-5% *Phase A* in *Phase B*, 18.5 min)]. ^{1}H NMR (CD_3OD, 500.13 MHz): δ 7.98 (s, 3H), 7.24-7.16 (m, 25H), 7.16-7.08 (m, 5H), 4.72-4.54 (m, 6H), 4.42-4.21 (m, 6H), 4.07-3.92 (m, 3H), 3.84 (s, 6H), 3.47-3.05 (m, 6H), 3.04-2.94 (m, 3H), 2.90-2.79 (m, 3H), 2.26-2.11 (m, 3H), 2.10-1.96 (m, 3H), 1.41 (s, 27H) [Supplementary Fig. 38, *top*]. ^{13}C NMR (CD_3OD, 125.77 MHz): δ 174.9, 173.5, 173.1, 157.7, 144.9, 138.6, 138.4, 130.6, 130.6, 129.6, 129.6, 127.8, 126.3, 81.0, 55.9, 55.7, 53.3, 48.5, 48.2, 39.1, 38.6, 33.9, 28.9 [Supplementary Fig. 38, *bottom*]. MS (ESI-TOF) [m/z (%)]: 885 ([M +Na]$^{+}$, 40), 874 ([MH]$^{+}$, 100). HRMS (ESI-TOF) calculated for $C_{90}H_{111}N_{19}O_{18}Na$ ([M +Na]$^{+}$) 1768.8247, found 1768.8245.

Tris(triazole) Boc-*L*-^{H}Ala-[*L*-Trp]$_2$-OFm. A solution of Boc-[*L*-Trp]$_2$-OFm (12.8 mg, 17.6 μmol) in 1 mL of TFA/CH_2Cl_2 (1:1) was stirred at rt for 15 min. The solvent was removed and the residue was dried under high vacuum for 3 h. The resulting TFA salt was disolved in dry CH_2Cl_2 (500 μL), after which tris(triazole) Boc-*L*-^{H}Ala-OH (**5**, 4.6 mg, 5.3 μmol), HATU (6.7 mg, 17.6 μmol) and DIEA (11.2 μL, 128.0 μmol) were successively added. The mixture was stirred for 1 h at rt and the solution was poured into a separating funnel and washed with HCl (5%) (3 × 500 μL) and $NaHCO_3$ (sat.) (2 × 500 μL). The organic layers were dried over Na_2SO_4 and concentrated under reduced pressure to give a yellow solid, which was purified by flash chromatography (0-7% MeOH in CH_2Cl_2) to give 9.6 mg of the desired tris(triazole) peptide [72%, R_f = 0.41 (7% MeOH in CH_2Cl_2), white solid]. ^{1}H NMR ($CDCl_3$, 500.13 MHz): δ 9.08 (br s, 3H), 8.53 (br s, 3H), 7.81-7.62 (m, 6H), 7.60-7.26 (m, 15H), 7.23-7.08 (m, 18H), 7.06-6.50 (m, 24H), 5.61-5.21 (m, 3H), 5.05-4.56 (m, 6H), 4.46-3.78 (m, 18H), 3.63 (br s, 6H), 3.37-3.2.82 (m, 12H), 2.16-1.70 (m, 6H), 1.35 (s, 27H) [Supplementary Fig. 39, *top*]. ^{13}C NMR ($CDCl_3$, 25.77 MHz): δ 173.8, 171.7, 171.1, 155.7, 143.7, 143.5, 141.4, 141.4, 136.3, 136.2, 131.1, 128.1, 128.1, 127.6, 127.4, 127.4, 127.3, 125.3, 125.2, 124.6, 124.0, 123.7, 122.3, 122.1, 120.2, 120.2, 119.8, 119.4, 118.8, 118.4, 111.7, 111.6, 109.9, 109.2, 80.6, 67.0, 54.2, 53.2, 51.8, 46.7, 46.3, 38.9, 34.2, 30.6, 29.1, 28.5 [Supplementary Fig. 39, *bottom*]. MS (ESI-TOF) [m/z (%)]: 1269 ([M +Na]$^{2+}$, 100), 1258 ([MH]$^{2+}$, 70). HRMS (ESI-TOF) calculated for $C_{129}H_{123}N_{25}O_{12}Na_2$ ([M +Na$_2$]$^{2+}$) 1280.0570, found 1280.0549.

Tris (triazole) Boc-*L*-^{H}Ala-[*L*-Trp]$_2$-OH (7). A solution of the tris(triazole) Boc-*L*-^{H}Ala-[*L*-Trp]$_2$-OFm (20.0 mg, 8.0 μmol) in 20% piperidine/CH_2Cl_2 (250 μL) was stirred at rt for 20 min and then the solvent was removed *in vacuo*. The residue was

dissolved in CH_2Cl_2 (250 μL) and washed with HCl (5 %) (2 × 250 μL), dried over Na_2SO_4, filtered and concentrated to give a white solid. The corresponding residue was dissolved in MeOH (200 μL), filtered using a 0.2 μm syringe-driven filter, and the crude solution was purified by HPLC, affording 12.3 mg of the tris(triazole) peptide **7** as a white solid [78%, t_R = 17.0 min (Zorbax SB-C18 semipreparative column, 60-5% *Phase A* in *Phase B*, 18.5 min)]. ^{1}H NMR (CD_3OD, 500.13 MHz): δ 7.75 (s, 3H), 7.55 (d, *J* = 8.0 Hz, 3H), 7.40-7.30 (m, 3H), 7.27 (d, *J* = 7.0 Hz, 3H, 3 × CH), 7.22 (d, *J* = 7.2 Hz, 3H), 7.08 (s, 3H), 7.03 (t, *J* = 6.4 Hz, 3H), 7.01-6.92 (m, 9H), 6.86 (t, *J* = 8.0 Hz, 3H), 4.74-4.64 (m, 3H), 4.63-4.54 (m, 3H), 4.19-4.05 (m, 3H), 4.04-3.94 (m, 3H), 3.94-3.82 (m, 3H), 3.71 (s, 6H), 3.47-3.13 (m, 9H), 3.13-3.00 (m, 3H), 2.15-1.95 (m, 3H), 1.91-1.74 (m, 3H), 1.37 (s, 27H) [Supplementary Fig. 40, *top*]. ^{13}C NMR (CD_3OD, 125.77 MHz): δ 173.6, 173.2, 169.7, 157.7, 145.0, 138.1, 137.9, 129.4, 129.0, 126.2, 124.9, 124.8, 122.5, 122.3, 120.0, 119.9, 119.8, 119.6, 112.4, 112.3, 111.4, 110.8, 81.0, 56.3, 55.6, 53.4, 48.9, 47.8, 33.5, 31.0, 28.9, 28.7 [Supplementary Fig. 40, *bottom*]. MS (ESI-TOF) [m/z (%)]: 991 ($[MH]^{2+}$, 100). HRMS (ESI-TOF) calculated for $C_{102}H_{119}N_{25}O_{18}$ ($[MH]^{2+}$) 990.9577, found 990.9573.

Tris(triazole) Boc-*L*-$^{\mathbf{H}}$Ala-[*L*-Ala]$_2$-OFm. A solution of Boc-[*L*-Ala]$_2$-OFm (25.1 mg, 57.3 μmol) in 500 μL of TFA/CH_2Cl_2 (1:1) was stirred at rt for 15 min. The solvent was removed and the residue was dried under high vacuum for 3 h. The resulting TFA salt was disolved in dry CH_2Cl_2 (500 μL), after which tris(triazole) Boc-*L*-^{H}Ala-OH (**5**, 15.0 mg, 17.4 μmol), HATU (21.8 mg, 57.3 μmol) and DIEA (36.3 μL, 208.3 μmol) were successively added. The mixture was stirred for 1 h at rt and the solution was poured into a separating funnel and washed with HCl (5%) (3 × 500 μL) and $NaHCO_3$ (sat.) (2 × 500 μL). The organic layers were dried over Na_2SO_4 and concentrated under reduced pressure to give a pale yellow solid, which was purified by flash chromatography (0-5% MeOH in CH_2Cl_2) to give 24.5 mg of the desired tris(triazole) peptide [77%, R_f = 0.25 (5% MeOH in CH_2Cl_2), white solid]. ^{1}H NMR ($CDCl_3$, 500.13 MHz): δ 7.80-7.66 (m, 6H), 7.63-7.48 (m, 6H), 7.43-7.34 (m, 6H), 7.33-7.25 (m, 6H), 7.24-7.10 (m, 3H), 7.03-6.42 (m, 6H), 5.57-5.23 (m, 3H), 4.68-4.13 (m, 24H), 4.29-4.05 (m, 3H), 3.68 (s, 6H), 2.47-2.01 (m, 6H), 1.46-1.26 (m, 45H) [Supplementary Fig. 41, *top*]. ^{13}C NMR ($CDCl_3$, 125.77 MHz): δ 172.9, 172.0, 171.1, 155.7, 148.5, 143.7, 143.4, 141.5, 141.5, 128.1, 128.1, 128.1, 127.4, 125.2, 125.1, 120.5, 120.3, 120.3, 80.5, 67.3, 51.2, 49.5, 48.4, 47.0, 46.9, 31.1, 29.9, 28.5, 18.2, 17.9 [Supplementary Fig. 41, *bottom*]. MS (ESI-TOF) [m/z (%)]: 924 ($[M +Na]^{2+}$, 100), 913 ($[MH]^{2+}$, 70). HRMS (ESI-TOF) calculated for $C_{96}H_{117}N_{19}O_{18}Na$ ($[M +Na]^+$) 1846.8716, found 1846.8718.

Tris(triazole) Boc-*L*-$^{\mathbf{H}}$Ala-[*L*-Ala]$_2$-OH (8). A solution of the tris(triazole) Boc-*L*-^{H}Ala-[*L*-Ala]$_2$-OFm (15.0 mg, 8.2 μmol) in 20% piperidine/CH_2Cl_2 (250 μL) was stirred at rt for 20 min and then the solvent was removed *in vacuo*. The residue was dissolved in CH_2Cl_2 (250 μL) and washed with HCl (5 %) (2 × 250 μL), dried over Na_2SO_4, filtered and concentrated to give a white solid. The corresponding residue was dissolved in MeOH (200 μL), filtered using a 0.2 μm syringe-driven filter, and the crude solution was purified by HPLC, affording 5.8 mg of the tris(triazole) peptide **8** as a white solid [55%, t_R = 12.7 min (Zorbax SB-C18 semipreparative column, 60-5% *Phase A* in *Phase B*, 18.5 min)]. ^{1}H NMR (CD_3OD, 500.13 MHz): δ 8.07 (s, 3H), 4.61-4.45 (m, 6H), 4.44-4.34 (m, 3H), 4.33-4.24 (m, 3H), 4.16-3.97 (m, 3H), 3.81 (s, 6H), 2.51-2.29 (m,

3H), 2.28-2.07 (m, 3H), 1.43 (s, 27H), 1.38 (d, $J = 7.2$ Hz, 18H) [Supplementary Fig. 42, *top*]. ^{13}C NMR (CD_3OD, 125.77 MHz): δ 176.8, 174.5, 173.7, 157.8, 145.2, 126.2, 81.0, 53.3, 50.6, 50.0, 49.8, 48.5, 33.8, 28.9, 18.3, 18.2 [Supplementary Fig. 42, *bottom*]. MS (ESI-TOF) [m/z (%)]: 657 ($[M + Na]^{2+}$, 25), 646 ($[MH]^{2+}$, 100). HRMS (ESI-TOF) calculated for $C_{54}H_{87}N_{19}O_{18}Na$ ($[M + Na]^{+}$) 1312.6369, found 1312.6352.

Autocatalytic Formation of Oligotriazole Peptide Nanospheres

Representative control for the autocatalytic formation of tris(triazole) Boc-*L*-^{H}Ala-OH (5) [0 equiv. of tris(triazole) peptide 5]. 187 μL of tripropargylamine (20 mM solution in 150 mM TES buffer pH 8.5 in H_2O; Final concentration: 5 mM), 187 μL of Boc-*L*-^{H}Ala(N_3)-OH.DCHA (**1**, 66 mM solution in 150 mM TES buffer pH 8.5 in H_2O; Final concentration: 16.5 mM) and 2 μL of 150 mM TES buffer pH 8.5 in H_2O were added to a 2 mL vial and stirred for 5 min at rt. Then, we added 187 μL of *(+)*-sodium *L*-ascorbate (20 mM solution in 150 mM TES buffer pH 8.5 in H_2O; Final concentration: 5 mM). Finally, 187 μL of $CuSO_4.5H_2O$ (10 mM solution in 150 mM TES buffer pH 8.5 in H_2O; Final concentration: 2.5 mM) were added. The heterogeneous mixture was stirred vigorously at rt under N_2. Periodically, 5 μL aliquots were removed and used directly to monitor the progress of the CuAAC reaction by LC-ELSD-MS. Tris(triazole) peptide **5** formation was not observed after 600 min.

Representative procedure for the autocatalytic formation of tris(triazole) Boc-*L*-^{H}Ala-OH (5) [0.016 equiv. of tris(triazole) peptide 5]. 187 μL of tripropargylamine (20 mM solution in 150 mM TES buffer pH 8.5 in H_2O; Final concentration: 5 mM), 187 μL of Boc-*L*-^{H}Ala(N_3)-OH.DCHA (**1**, 66 mM solution in 150 mM TES buffer pH 8.5 in H_2O; Final concentration: 16.5 mM) and 2 μL of tris(triazole) Boc-*L*-^{H}Ala-OH (**5**, 30 mM solution in 150 mM TES buffer pH 8.5 in H_2O; Final concentration: 80 μM) were added to a 2 mL vial and stirred for 5 min at rt. Then, we added 187 μL of *(+)*-sodium *L*-ascorbate (20 mM solution in 150 mM TES buffer pH 8.5 in H_2O; Final concentration: 5 mM). Finally, 187 μL of $CuSO_4.5H_2O$ (10 mM solution in 150 mM TES buffer pH 8.5 in H_2O; Final concentration: 2.5 mM) were added. The heterogeneous mixture was stirred vigorously at rt under N_2. Periodically, 5 μL aliquots were removed and used directly to monitor the progress of the CuAAC reaction by LC-ELSD-MS.

Representative procedure for the autocatalytic formation of tris(triazole) Boc-*L*-^{H}Ala-OH (5) [0.1 equiv. of tris(triazole) peptide 5]. 187 μL of tripropargylamine (20 mM solution in 150 mM TES buffer pH 8.5 in H_2O; Final concentration: 5 mM), 187 μL of Boc-*L*-^{H}Ala(N_3)-OH.DCHA (**1**, 66 mM solution in 150 mM TES buffer pH 8.5 in H_2O; Final concentration: 16.5 mM) and 2 μL of tris(triazole) Boc-*L*-^{H}Ala-OH (**5**, 187.5 mM solution in 150 mM TES buffer pH 8.5 in H_2O; Final concentration: 500 μM) were added to a 2 mL vial and stirred for 5 min at rt. Then, we added 187 μL of *(+)*-sodium *L*-ascorbate (20 mM solution in 150 mM TES buffer pH 8.5 in H_2O; Final concentration: 5 mM). Finally, 187 μL of $CuSO_4.5H_2O$ (10 mM solution in 150 mM TES buffer pH 8.5 in H_2O; Final concentration: 2.5 mM) were added. The heterogeneous mixture was stirred vigorously at rt under N_2. Periodically, 5 μL aliquots were removed and used directly to monitor the progress of the CuAAC reaction by LC-ELSD-MS.

Representative procedure for the autocatalytic formation of tris(triazole) Boc-*L*-^{H}Ala-[*L*-Trp]$_2$-OH (7) [0.01 equiv. of tris(triazole) peptide 7]. 18.7 μL of tripropargylamine (4 mM solution in 150 mM TES buffer pH 8.5 in H_2O; Final concentration: 1 mM), 18.7 μL of Boc-*L*-^{H}Ala-[*L*-Trp]$_2$-OH (**3**, 13.2 mM solution in 150 mM TES buffer pH 8.5 in H_2O; Final concentration: 3.3 mM) and 0.2 μL of tris(triazole) Boc-*L*-^{H}Ala-[*L*-Trp]$_2$-OH (**7**, 3.8 mM solution in 150 mM TES buffer pH 8.5 in H_2O; Final concentration: 10 μM) were added to a 2 mL vial and stirred for 5 min at rt. Then, we added 18.7 μL of *(+)*-sodium *L*-ascorbate (4 mM solution in 150 mM TES buffer pH 8.5 in H_2O; Final concentration: 1 mM). Finally, 18.7 μL of $CuSO_4.5H_2O$ (2 mM solution in 150 mM TES buffer pH 8.5 in H_2O; Final concentration: 500 μM) were added. The heterogeneous mixture was stirred vigorously at rt under N_2. Periodically, 5 μL aliquots were removed and used directly to monitor the progress of the CuAAC reaction by LC-ELSD-MS. Autocatalytic formation of tris(triazole) peptides **6** and **8** was studied in the same way, using the corresponding azidopeptides **2** and **4**.

<u>Serial Transfers: Continual Synthesis of Catalytic Oligotriazole Peptide Nanospheres</u>

Representative control for the continual synthesis of tris(triazole) Boc-*L*-^{H}Ala-OH (5) [1.6% transfer; 0 equiv. of tris(triazole) peptide 5]. 184.5 μL of tripropargylamine (20.32 mM solution in 150 mM TES buffer pH 8.5 in H_2O; Final concentration: 5 mM), 184.5 μL of Boc-*L*-^{H}Ala(N_3)-OH.DCHA (**1**, 67.07 mM solution in 150 mM TES buffer pH 8.5 in H_2O; Final concentration: 16.5 mM) and 12 μL of 150 mM TES buffer pH 8.5 in H_2O) were added to a 2 mL vial and stirred for 5 min at rt. Then, we added 184.5 μL of *(+)*-sodium *L*-ascorbate (20.32 mM solution in 150 mM TES buffer pH 8.5 in H_2O; Final concentration: 5 mM). Finally, 184.5 μL of $CuSO_4.5H_2O$ (10.16 mM solution in 150 mM TES buffer pH 8.5 in H_2O; Final concentration: 2.5 mM) were added. The heterogeneous mixture was stirred vigorously at rt under N_2. Periodically, 5 μL aliquots were removed and used directly to monitor the progress of the CuAAC reaction by LC-ELSD-MS. Tris(triazole) peptide **5** formation was not observed after ≈480 min. Afterwards, a fraction (1.6%; 12 μL) of the reaction mixture was isolated and combined (≈510 min) with a fresh precursor solution [184.5 μL of tripropargylamine (20.32 mM solution in 150 mM TES buffer pH 8.5 in H_2O; Final concentration: 5 mM), 184.5 μL of Boc-*L*-^{H}Ala(N_3)-OH.DCHA (**1**, 67.07 mM solution in 150 mM TES buffer pH 8.5 in H_2O; Final concentration: 16.5 mM), 184.5 μL of *(+)*-sodium *L*-ascorbate (20.32 mM solution in 150 mM TES buffer pH 8.5 in H_2O; Final concentration: 5 mM) and 184.5 μL of $CuSO_4.5H_2O$ (10.16 mM solution in 150 mM TES buffer pH 8.5 in H_2O; Final concentration: 2.5 mM)]. Subsequent transfers (×3) and additions (×3) were made. Periodically, 5 μL aliquots were removed and used directly to monitor the progress of the CuAAC reaction by LC-ELSD-MS. Tris(triazole) peptide **5** formation was not observed after ≈2010 min.

Representative procedure for the continual synthesis of tris(triazole) Boc-*L*-^{H}Ala-OH (5) [1.6% transfer; 0.016 equiv. of tris(triazole) peptide 5]. 184.5 μL of tripropargylamine (20.32 mM solution in 150 mM TES buffer pH 8.5 in H_2O; Final concentration: 5 mM), 184.5 μL of Boc-*L*-^{H}Ala(N_3)-OH.DCHA (**1**, 67.07 mM solution in 150 mM TES buffer pH 8.5 in H_2O; Final concentration: 16.5 mM) and 12 μL of tris(triazole) Boc-*L*-^{H}Ala-OH (**5**, 5 mM solution in 150 mM TES buffer pH 8.5 in H_2O; Final concentration: 80 μM) were added to a 2 mL vial and stirred for 5 min at rt. Then,

we added 184.5 μL of *(+)*-sodium *L*-ascorbate (20.32 mM solution in 150 mM TES buffer pH 8.5 in H_2O; Final concentration: 5 mM). Finally, 184.5 μL of $CuSO_4.5H_2O$ (10.16 mM solution in 150 mM TES buffer pH 8.5 in H_2O; Final concentration: 2.5 mM) were added. The heterogeneous mixture was stirred vigorously at rt under N_2. Periodically, 5 μL aliquots were removed and used directly to monitor the progress of the CuAAC reaction by LC-ELSD-MS. Once the precursors were depleted (≈480 min), a fraction (1.6%; 12 μL) of the nanosphere population wass isolated and combined (≈510 min) with a fresh precursor solution [184.5 μL of tripropargylamine (20.32 mM solution in 150 mM TES buffer pH 8.5 in H_2O; Final concentration: 5 mM), 184.5 μL of Boc-*L*-^{H}Ala(N_3)-OH.DCHA (**1**, 67.07 mM solution in 150 mM TES buffer pH 8.5 in H_2O; Final concentration: 16.5 mM), 184.5 μL of *(+)*-sodium *L*-ascorbate (20.32 mM solution in 150 mM TES buffer pH 8.5 in H_2O; Final concentration: 5 mM) and 184.5 μL of $CuSO_4.5H_2O$ (10.16 mM solution in 150 mM TES buffer pH 8.5 in H_2O; Final concentration: 2.5 mM)]. Subsequent transfers (×3) and additions (×3) were made. Periodically, 5 μL aliquots were removed and used directly to monitor the progress of the CuAAC reaction by LC-ELSD-MS.

Representative procedure for the continual synthesis of tris(triazole) Boc-*L*-^{H}Ala-OH (5) [10% transfer; from 0.016 equiv. to 0.1 equiv. of tris(triazole) peptide 5]. 184.5 μL of tripropargylamine (20.32 mM solution in 150 mM TES buffer pH 8.5 in H_2O; Final concentration: 5 mM), 184.5 μL of Boc-*L*-^{H}Ala(N_3)-OH.DCHA (**1**, 67.07 mM solution in 150 mM TES buffer pH 8.5 in H_2O; Final concentration: 16.5 mM) and 12 μL of tris(triazole) Boc-*L*-^{H}Ala-OH (**5**, 5 mM solution in 150 mM TES buffer pH 8.5 in H_2O; Final concentration: 80 μM) were added to a 2 mL vial and stirred for 5 min at rt. Then, we added 184.5 μL of *(+)*-sodium *L*-ascorbate (20.32 mM solution in 150 mM TES buffer pH 8.5 in H_2O; Final concentration: 5 mM). Finally, 184.5 μL of $CuSO_4.5H_2O$ (10.16 mM solution in 150 mM TES buffer pH 8.5 in H_2O; Final concentration: 2.5 mM) were added. The heterogeneous mixture was stirred vigorously at rt under N_2. Periodically, 5 μL aliquots were removed and used directly to monitor the progress of the CuAAC reaction by LC-ELSD-MS. Once the precursors were depleted (≈480 min), a fraction (10%; 75.2 μL) of the nanosphere population was isolated and combined (≈540 min) with a new precursor solution [168.7 μL of tripropargylamine (22.22 mM solution in 150 mM TES buffer pH 8.5 in H_2O; Final concentration: 5 mM), 168.7 μL of Boc-*L*-^{H}Ala(N_3)-OH.DCHA (**1**, 73.36 mM solution in 150 mM TES buffer pH 8.5 in H_2O; Final concentration: 16.5 mM), 168.7 μL of *(+)*-sodium *L*-ascorbate (22.22 mM solution in 150 mM TES buffer pH 8.5 in H_2O; Final concentration: 5 mM) and 168.7 μL of $CuSO_4.5H_2O$ (11.11 mM solution in 150 mM TES buffer pH 8.5 in H_2O; Final concentration: 2.5 mM)]. Periodically, 5 μL aliquots were removed and used directly to monitor the progress of the CuAAC reaction by LC-ELSD-MS. Once the precursors were depleted (≈660 min), a fraction (10%; 75.2 μL) of the nanosphere population was isolated and combined (≈720 min) with a fresh precursor solution. Subsequent transfers (×3) and additions (×3) were made. Periodically, 5 μL aliquots were removed and used directly to monitor the progress of the CuAAC reaction by LC-ELSD-MS.

LC/ELSD/MS Analysis

CuAAC reactions were performed in the appropriate buffer as described above. Aliquots of 5 μL of sample were taken at various time points, and analyzed using an Eclipse Plus C8 analytical column [60-5% *Phase A* in *Phase B*, 5.5 min for tris(triazole) peptide **5**; 40-5% *Phase A* in *Phase B* , 5.5 min for tris(triazole) peptides **6**-**8**;] with an Evaporative Light Scattering Detector (ELSD) at a flow of 1.0 mL/min. For all LC/MS runs, solvent *Phase A* consisted of H_2O with 0.1% formic acid and solvent *Phase B* of MeOH with 0.1% formic acid.

Transmission Electron Microscopy (TEM) Studies

General. A deposition System Balzers Med010 was used to evaporate a homogeneous layer of carbon. The samples were collected over 400 mesh Cu grids. The grids were then negatively stained with a solution of 1% (w/w) uranyl acetate. Micrographs were recorded on a FEI Tecnai™ Sphera microscope operating at 200 kV and equipped with a LaB_6 electron gun, using the standard cryotransfer holders developed by Gatan, Inc. For image processing, micrographs were digitized in a Zess SCAI scanner with different sampling windows.

TEM measurements. Copper grids (formvar/carbon-coated, 400 mesh copper) were prepared by glow discharging the surface at 20 mA for 1.5 min. Once the surface for vesicle adhesion is ready, 3.5 μL of a 5 mM solution of tris(triazole) peptide (**5**, **6**, **7** or **8**) in H_2O or organic-aqueous solution (previously tumbled at rt for 24 h) was deposited on the grid surface. This solution was allowed to sit for 10 seconds before being washed away with 10 drops of glass distilled H_2O and subsequent staining with 3 drops of 1% w/w uranyl acetate. The stain was allowed to sit for 10 seconds before wicking away with filter paper. All grid treatments and simple depositions were on the dark/shiny/glossy formvar-coated face of the grid (this side face up during glow discharge). Samples were then imaged *via* TEM, revealing the presence of several populations of spherical compartments (50-950 nm in diameter), consistent with the vesicle architecture.

Phase Contrast Microscopy: Nanosherical Structures

Hydration method. 50.0 μL of a 10 mM solution of tris(triazole) peptide (**5**, **6**, **7** or **8**) in MeOH were added to a 1 mL vial, placed under N_2 and dried for 15 min to prepare a peptide film. Then, 100 μL of H_2O or organic-aqueous solution [MeOH:H_2O (1:1) or MeOH:H_2O (7:3)] were added, and the solution was tumbled at 25 ºC for 4 h. The peptide-containing solutions were finally monitored by phase contrast microscopy in order to determine the nanosphere structure.

Sonication method. 10 μL of a 10 mM solution of (**5**, **6**, **7** or **8**) in MeOH was added to a 1 mL vial, placed under N_2 and dried for 15 min to prepare a lipid film. Then, 100 μL of H_2O or organic-aqueous solution [MeOH:H_2O (1:1) or MeOH:H_2O (7:3)] were added, and the resulting mixture was sonicated with heat (≈55ºC) for 1 h. The corresponding mixture was finally monitored by phase contrast microscopy in order to determine the nanosphere structure.

Dynamic Light Scattering (DLS) Measurements

10.0 μL of a 10 mM solution of tris(triazole) peptide (**5**, **7** or **8**) in MeOH were added to a 1 mL vial, placed under N_2 and dried for 15 min to prepare a peptide film. Then, 100 μL of H_2O were added, and the solution was tumbled at 25 ºC for 24 h. The peptide-containing solutions were finally analyzed by DLS, which corroborated the nanostructure sizes.

Encapsulation Experiments

Encapsulation of HPTS/Rhodamine B. 50.0 μL of a 10 mM solution of tris(triazole) peptide (**5**, **7** or **8**) in MeOH were added to a 1 mL vial, placed under N_2 and dried for 15 min to prepare a peptide film. Then, 100 μL of 50 μM dye (HPTS or rhodamine B) in H_2O were added, and the solution was tumbled at 25 ºC for 24 h. Afterward, the mixture was transferred to a 100K spin filter and centrifuged for 10 min at 9000-10000 rcf in order to remove the non-encapsulated dye. Then, the sample was washed and centrifuged with H_2O (5 × 250 μL). The peptide-containing solution was finally examined by fluorescence microscopy, observing small spheres containing the desired dye.

Release Experiments

Release of HPTS/Rhodamine B. Release of the dye was obtained upon acidification of a 100 μL aqueous solution of self-assembled peptide (**5**, **7** or **8**) nanospheres containing dye (HPTS or rhodamine) at pH 5.5 and subsequent strirring at rt for 24 h. The peptide-containing solution was finally examined by phase contrast and fluorescence microscopy, observing small spheres without dye entrapped.

Nature of the Catalytically Species Experiments

50.0 μL of a 58 mM solution of tris(triazole) peptide **5** in MeOH were added to a 1 mL vial, placed under N_2 and dried for 15 min to prepare a peptide film. Then, 100 μL of H_2O were added, and the solution was tumbled at 25 ºC for 24 h. Afterward, the mixture was transferred to a 30K spin filter and centrifuged for 10 min at 9000-10000 rcf. HPLC/ELSD traces show that filtrate and nanospheres solutions present approximately the same concentration of tris(triazole) peptide **5**. Both solutions were finally analized by DLS, which showed population sizes of approximately 150 nm.

Continued Production of Tris(triazole) on the Nanospheres: Growth

Generation of self-assembled peptide nanospheres formed from tris(triazole)Boc-L-^{H}Ala-OH (5) [Hydration method]. 100.0 μL of a 30 mM solution of tris(triazole) peptide **5** in MeOH were added to a 1 mL vial, placed under N_2 and dried for 15 min to prepare a peptide film. Then, 100 μL of 150 mM TES buffer pH 8.5 in H_2O were added, and the solution was tumbled at 25 ºC for 2 h.

Growth of self-assembled peptide nanospheres formed from tris(triazole)Boc-L-^{H}Ala-OH (5) under standard CuAAC autocatalytic conditions [DLS measurements over time]. 187 μL of tripropargylamine (20 mM solution in 150 mM TES buffer pH 8.5 in H_2O; Final concentration: 5 mM), 187 μL of Boc-*L*-^{H}Ala(N_3)-OH.DCHA (**1**, 66 mM solution in 150 mM TES buffer pH 8.5 in H_2O; Final concentration: 16.5 mM) and 2 μL of self-assembled peptide nanospheres formed

from tris(triazole) Boc-*L*-^{H}Ala-OH (**5**, 30 mM solution in 150 mM TES buffer pH 8.5 in H_2O; Final concentration: 80 μM) were added to a 2 mL vial and stirred for 5 min at rt. Then, we added 187 μL of *(+)*-sodium *L*-ascorbate (20 mM solution in 150 mM TES buffer pH 8.5 in H_2O; Final concentration: 5 mM). Finally, 187 μL of $CuSO_4.5H_2O$ (10 mM solution in 150 mM TES buffer pH 8.5 in H_2O; Final concentration: 2.5 mM) were added. The heterogeneous mixture was stirred vigorously at rt under N_2. Periodically, 100 μL aliquots were removed and used directly to monitor the progress of the peptide nanospheres growth by DLS.

Selectivity Experiments

Representative procedure for the selective formation of tris(triazole) Boc-*L*-^{H}Ala-[*L*-Trp]$_2$-OH (7). 18.7 μL of tripropargylamine (4 mM solution in 150 mM TES buffer pH 8.5 in H_2O; Final concentration: 1 mM), 9.35 μL of Boc-*L*-^{H}Ala-[*L*-Trp]$_2$-OH (**3**, 26.4 mM solution in 150 mM TES buffer pH 8.5 in H_2O; Final concentration: 3.3 mM), 9.35 μL of Boc-*L*-^{H}Ala-[*L*-Ala]$_2$-OH (**4**, 26.4 mM solution in 150 mM TES buffer pH 8.5 in H_2O; Final concentration: 3.3 mM) and 0.2 μL of tris(triazole) Boc-*L*-^{H}Ala-[*L*-Trp]$_2$-OH (**7**, 3.8 mM solution in 150 mM TES buffer pH 8.5 in H_2O; Final concentration: 10 μM) were added to a 2 mL vial and stirred for 5 min at rt. Then, we added 18.7 μL of *(+)*-sodium *L*-ascorbate (4 mM solution in 150 mM TES buffer pH 8.5 in H_2O; Final concentration: 1 mM). Finally, 18.7 μL of $CuSO_4.5H_2O$ (2 mM solution in 150 mM TES buffer pH 8.5 in H_2O; Final concentration: 500 μM) were added. The heterogeneous mixture was stirred vigorously at rt under N_2. Periodically, 5 μL aliquots were removed and used directly to monitor the progress of the CuAAC reaction by LC-ELSD-MS. Tris(triazole) peptide **7** selective formation was successfully observed.

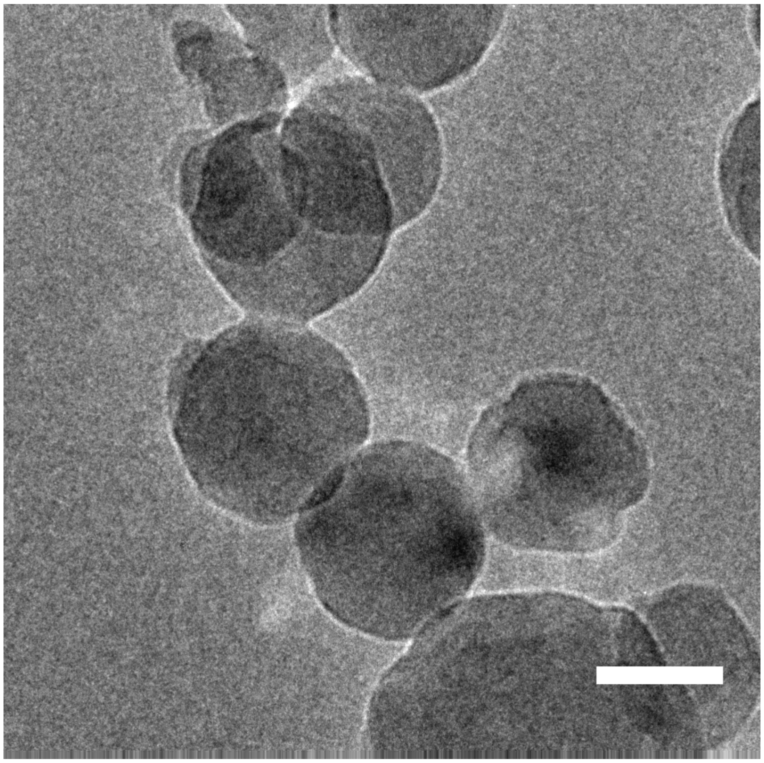

Supplementary Figure 1. Characterization of peptide nanospherical architecture. Cryo-EM image of spontaneously formed self-assembled peptide nanospheres from an aqueous solution of tris(triazole) Boc-*L*-^{H}Ala-OH (**5**). Scale bar denotes 100 nm.

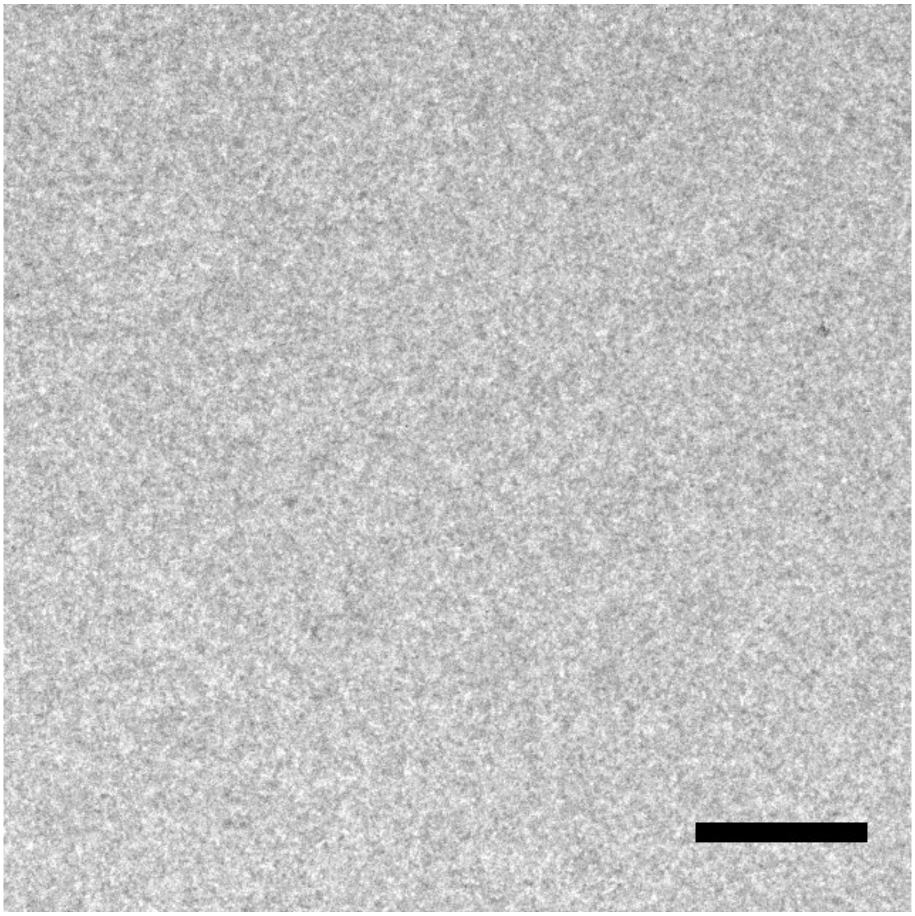

Supplementary Figure 2. Negative staining TEM image of an aqueous solution of azido-modified peptide precursor Boc-*L*-^{H}Ala(N_3)-OH.DCHA (**1**). Formation of self-assembled nanospheres from azidopeptide derivative was not observed. Scale bar denotes 250 nm.

a)

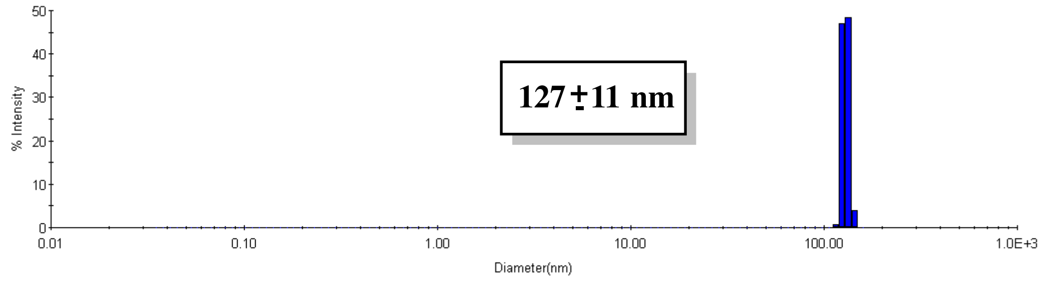

b)

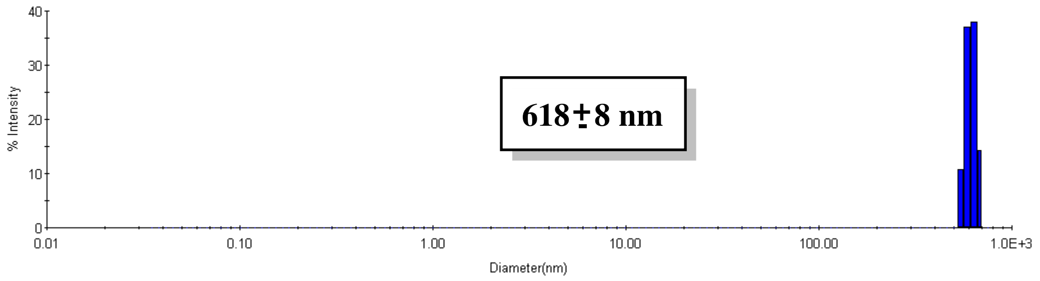

c)

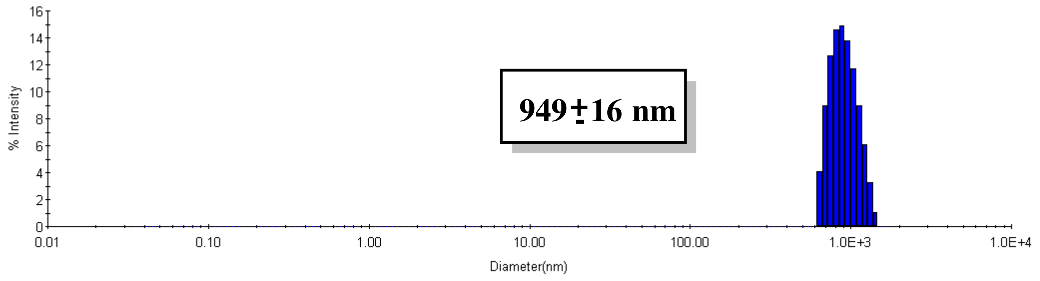

Supplementary Figure 3. Dynamic Light Scattering corresponding to 1.0 mM aqueous solution of tris(triazole) peptide **5** (**a**), **7** (**b**) and **8** (**c**).

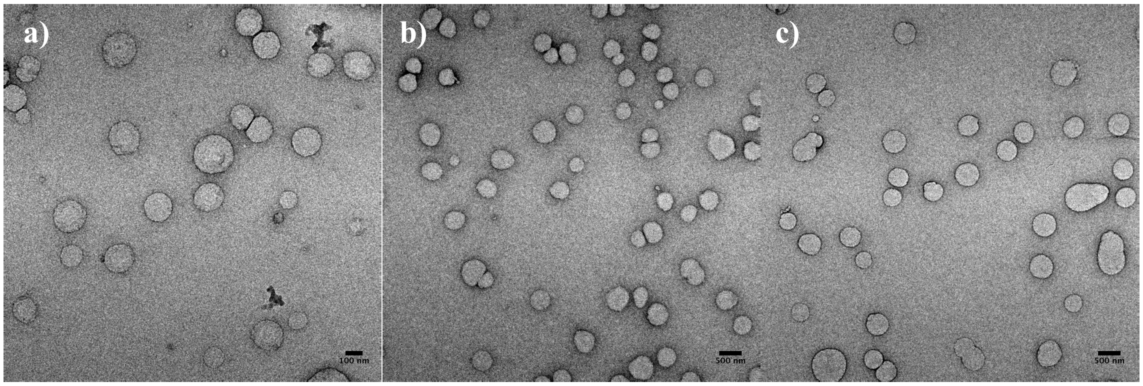

Supplementary Figure 4. Characterization of peptide nanospherical structures in organic-aqueous solution. TEM images of negatively stained self-assembled peptide nanospheres formed from oligotriazole peptide **5** (**a**), **7** (**b**) and **8** (**c**) in MeOH/H_2O (1:1) [Scale bar denotes 100 nm for **a** and 500 nm for **b** an **c**].

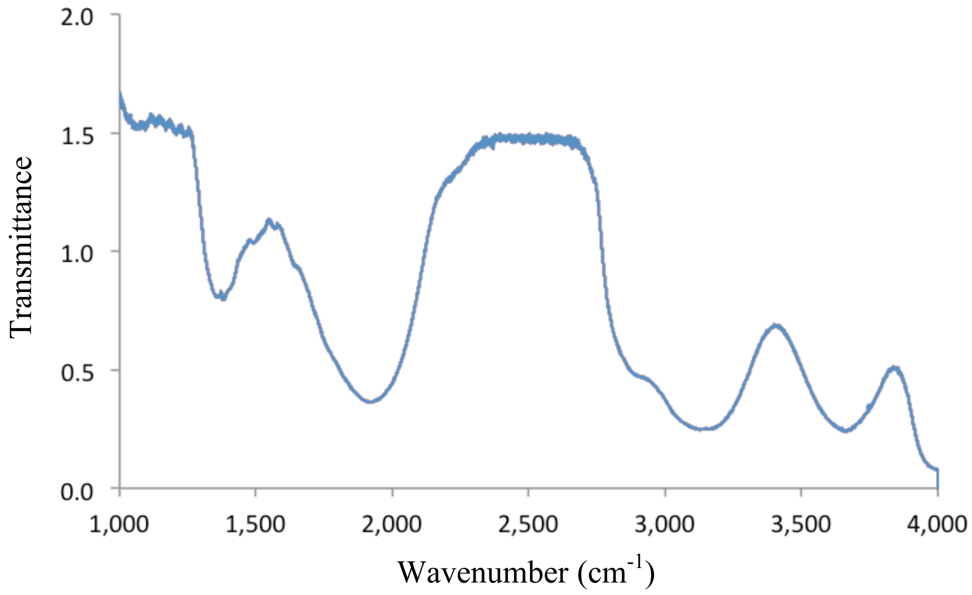

Supplementary Figure 5. Conformation of the peptide backbones. FT-IR spectrum of tris(triazole) Boc-*L*-^{H}Ala-OH (**5**) in D_2O.

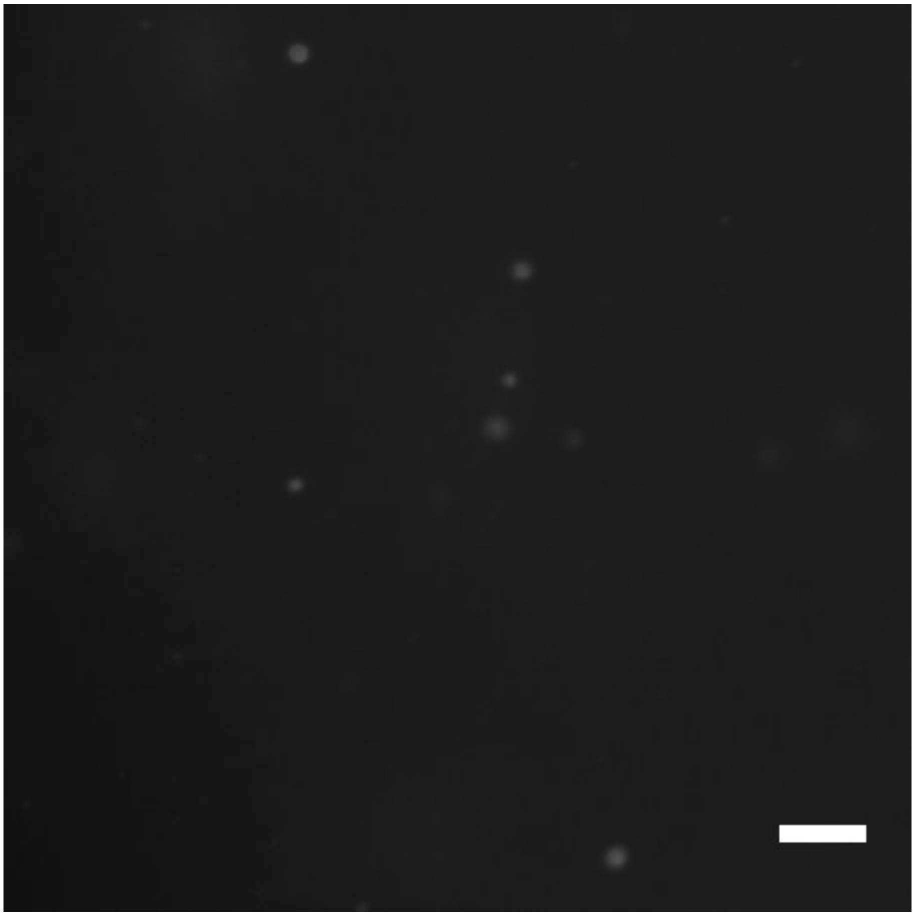

Supplementary Figure 6. Fluorescent microscope image demonstrating the entrapment of Rhodamine B in tris(triazole) Boc-*L*-^{H}Ala-OH (**5**) nanospheres. Scale bar denotes 10 µm.

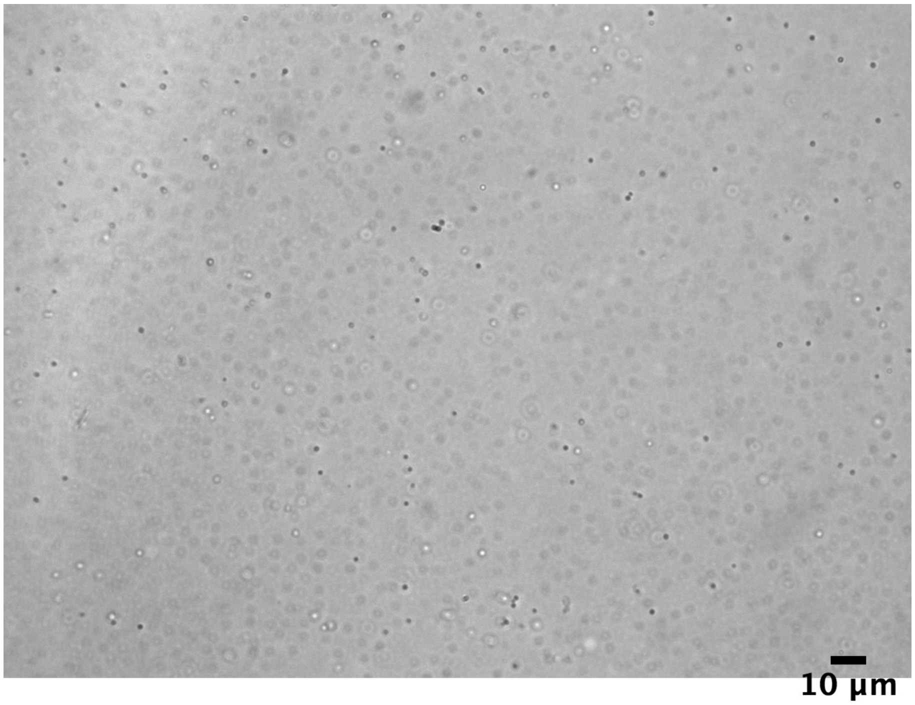

Supplementary Figure 7. Release of dye (Rhodamine B) upon acidification of tris(triazole) Boc-*L*-^{H}Ala-OH (**5**) nanospheres at pH 5.5 after 24 h. Phase contrast image showing the empty self-assembled nanospheres.

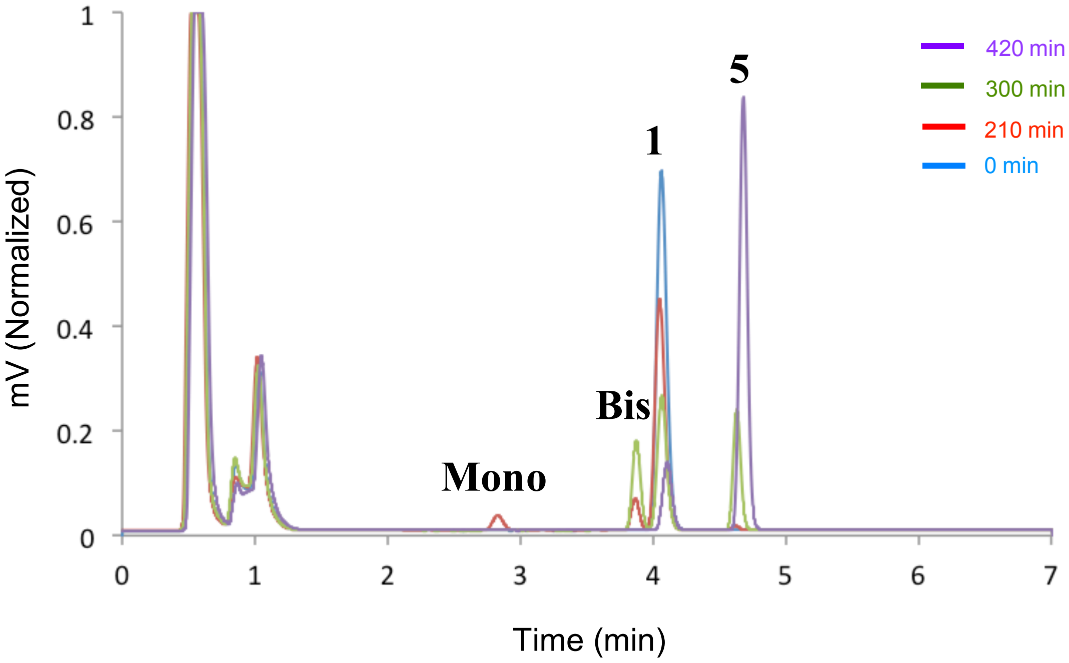

Supplementary Figure 8. Autocatalytic formation of tris(triazole) Boc-*L*-^{H}Ala-OH (**5**) by cycloaddition reaction of tripropargylamine with the azidoderivative Boc-*L*-^{H}Ala(N_3)-OH.DCHA (**1**) in the presence of 0.016 equiv. of tris(triazole) **5**. ELSD traces monitoring the progress of the CuAAC reaction. The retention times for all the species were verified by mass spectrometry and the use of known standards.

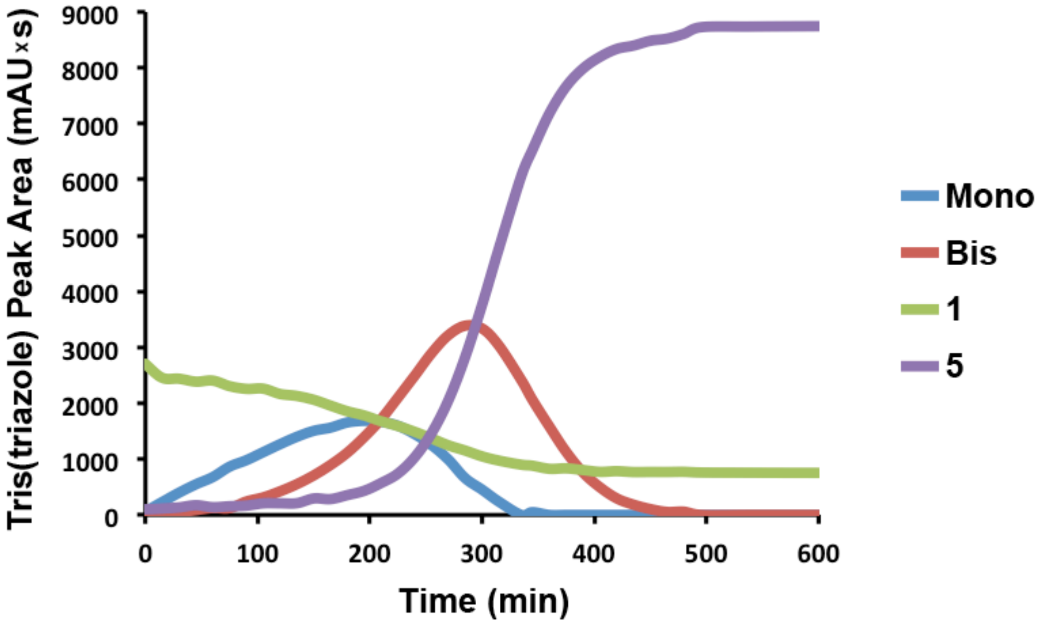

Supplementary Figure 9. Kinetics of the formation and/or consumption of all the species involved in the autocatalytic formation of tris(triazole) Boc-*L*-^{H}Ala-OH (**5**) by cycloaddition reaction of tripropargylamine with the azidoderivative Boc-*L*-^{H}Ala(N_3)-OH.DCHA (**1**) in the presence of 0.016 equiv. of tris(triazole) **5**. HPLC spectra (210 nm) were used to monitor the progress of the reactions. The retention times for all the species were verified by mass spectrometry and the use of known standards.

a)

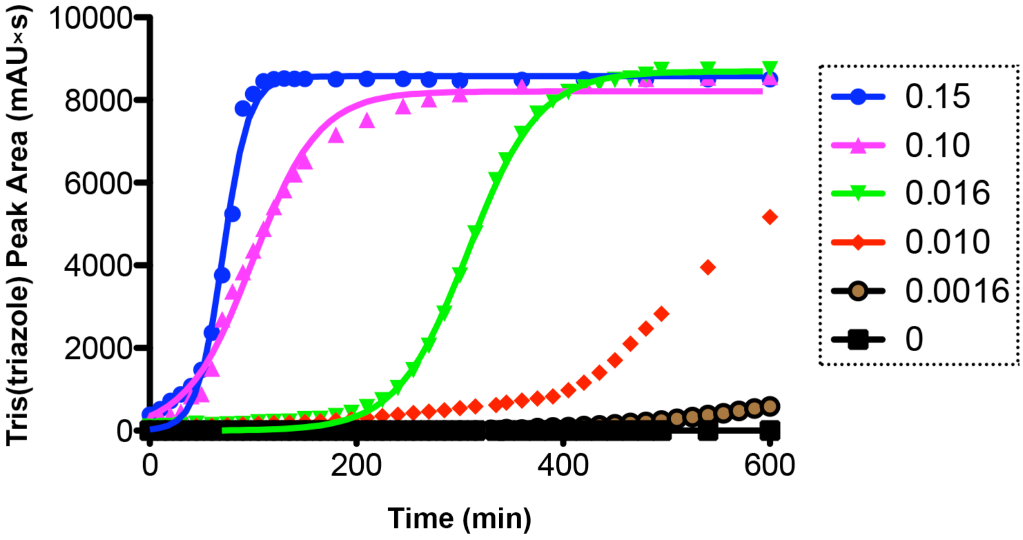

b)

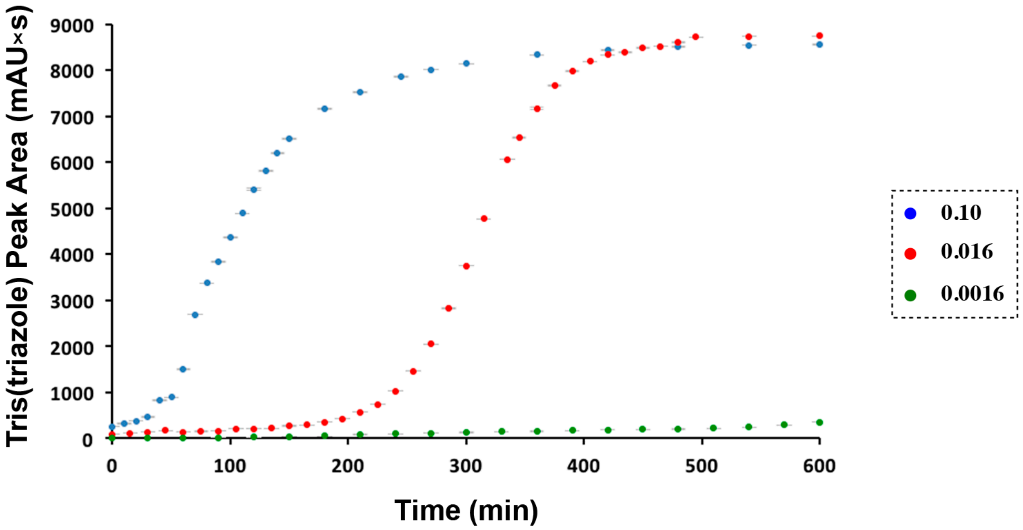

Supplementary Figure 10. Autocatalytic formation of tris(triazole) Boc-*L*-^{H}Ala-OH (**5**). **a)** Autocatalytic formation of tris(triazole) Boc-*L*-^{H}Ala-OH (**5**) by cycloaddition reaction of tripropargylamine with the azidoderivative Boc-*L*-^{H}Ala(N_3)-OH.DCHA (**1**) in the presence of 0, 0.0016, 0.01, 0.016, 0.1 or 0.15 equivalents of tris(triazole) peptide **5**. Sigmoidal growth curves are observed and can be fit to a four-parameter logistic equation (solid lines), which are typically used to describe autocatalytic processes. HPLC spectra (210 nm) were used to monitor the progress of the CuAAC reaction. The retention times for all the species were verified by mass spectrometry and the use of known standards. **b)** Representative examples of autocatalytic formation of tris(triazole) Boc-*L*-^{H}Ala-OH (**5**), showing the experimental errors (n = 3).

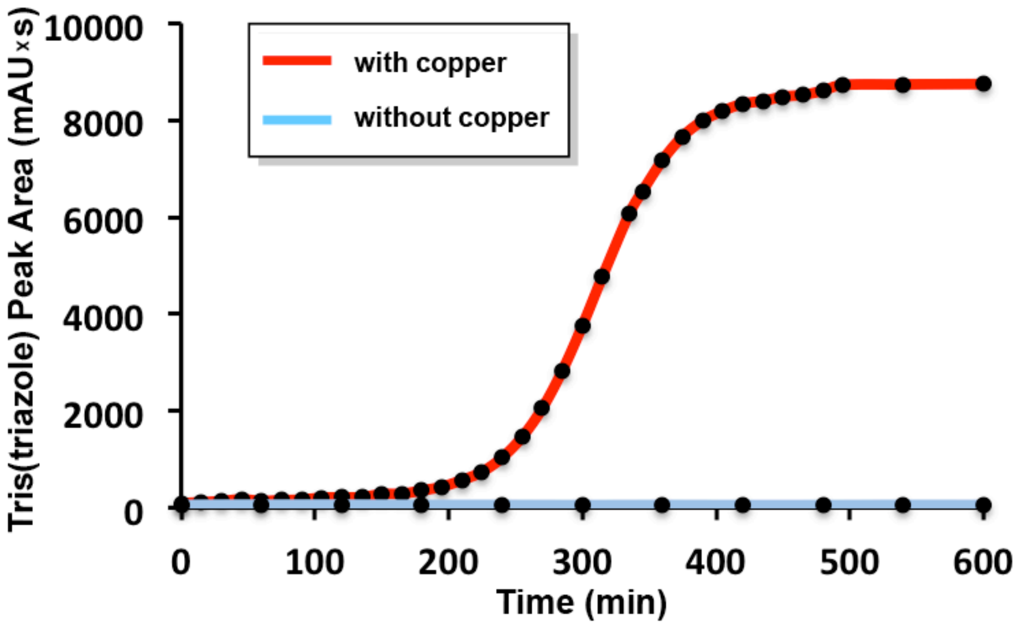

Supplementary Figure 11. Autocatalytic formation of tris(triazole) Boc-*L*-^{H}Ala-OH (**5**) by cycloaddition reaction of tripropargylamine with the azidoderivative Boc-*L*-^{H}Ala(N_3)-OH.DCHA (**1**) in the presence of 0.016 equiv. of tris(triazole) **5** and the absence (light blue line) or presence (red line) of $CuSO_4.5H_2O$. HPLC spectra (210 nm) were used to monitor the progress of the CuAAC reaction. The retention times for all the species were verified by mass spectrometry and the use of known standards.

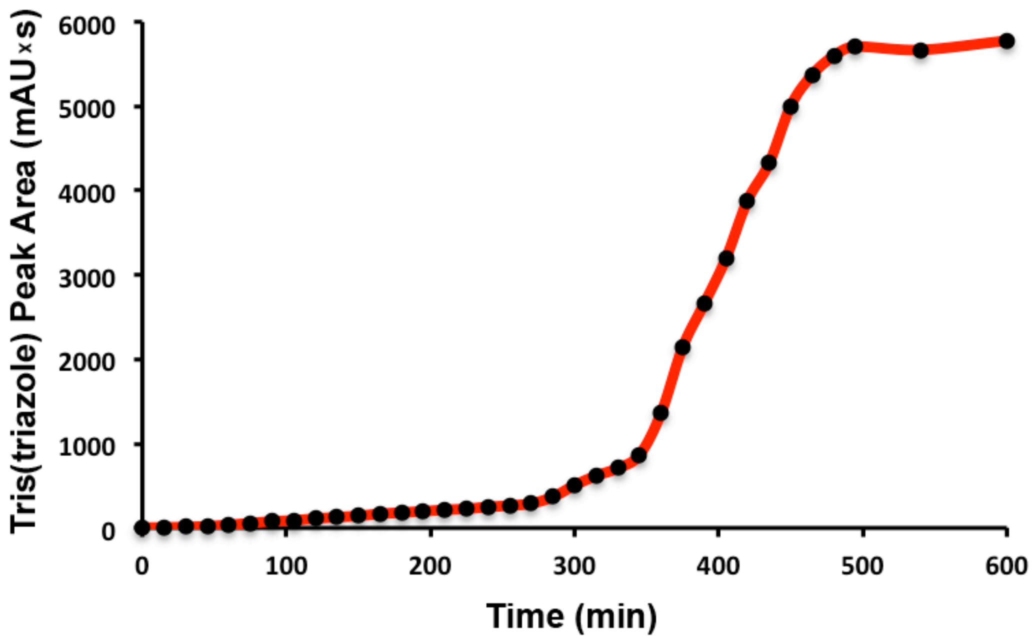

Supplementary Figure 12. Autocatalytic formation of tris(triazole) Boc-*L*-HAla-[*L*-Phe]$_2$-OH (**6**) by cycloaddition reaction of tripropargylamine with the azidoderivative Boc-*L*-HAla(N_3)-[*L*-Phe]$_2$-OH (**2**) in the presence of 0.01 equivalents of tris(triazole) peptide **6**. HPLC spectra (210 nm) were used to monitor the progress of the CuAAC reaction. Retention times were verified by mass spectrometry.

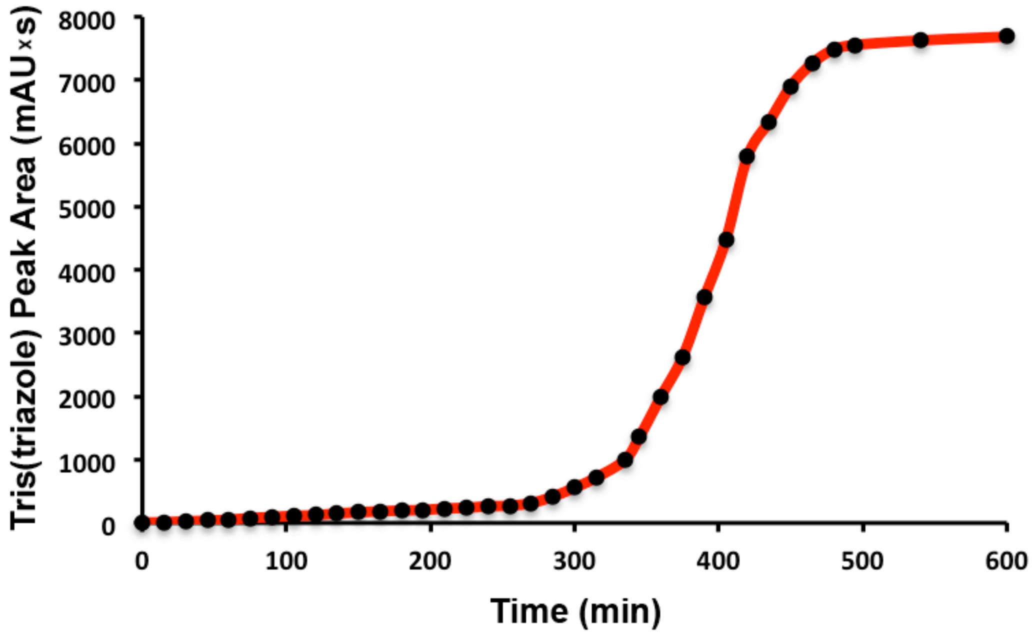

Supplementary Figure 13. Autocatalytic formation of tris(triazole) Boc-*L*-[H]Ala-[*L*-Trp]$_2$-OH (**7**) by cycloaddition reaction of tripropargylamine with the azidoderivative Boc-*L*-[H]Ala(N_3)-[*L*-Trp]$_2$-OH (**3**) in the presence of 0.01 equivalents of tris(triazole) peptide **7**. HPLC spectra (210 nm) were used to monitor the progress of the CuAAC reaction. Retention times were verified by mass spectrometry.

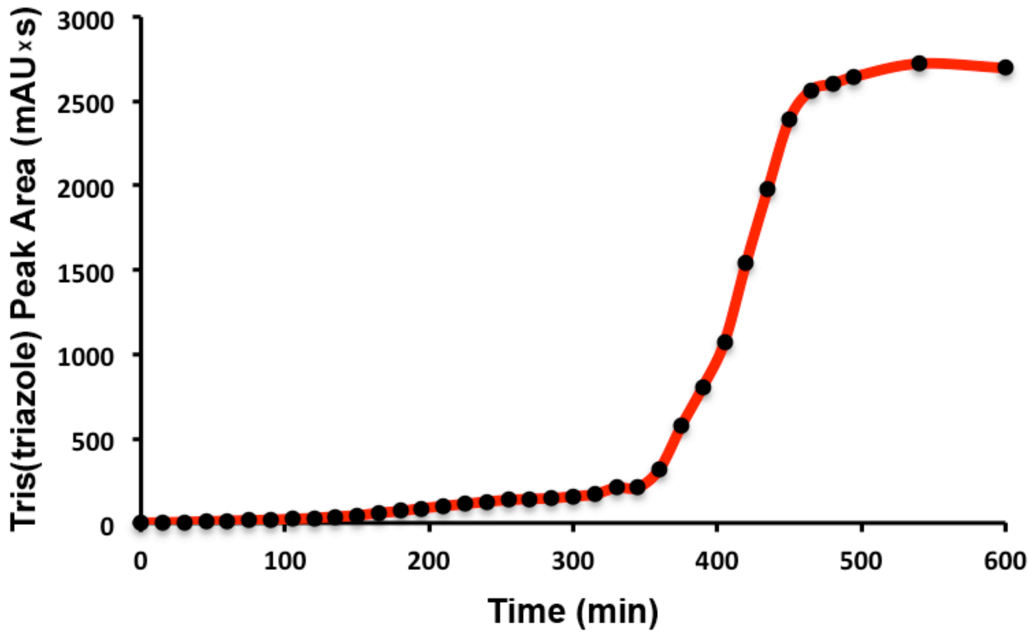

Supplementary Figure 14. Autocatalytic formation of tris(triazole) Boc-*L*-^{H}Ala-[*L*-Ala]$_2$-OH (**8**) by cycloaddition reaction of tripropargylamine with the azidoderivative Boc-*L*-^{H}Ala(N_3)-[*L*-Trp]$_2$-OH (**4**) in the presence of 0.01 equivalents of tris(triazole) peptide **8**. HPLC spectra (210 nm) were used to monitor the progress of the CuAAC reaction. Retention times were verified by mass spectrometry.

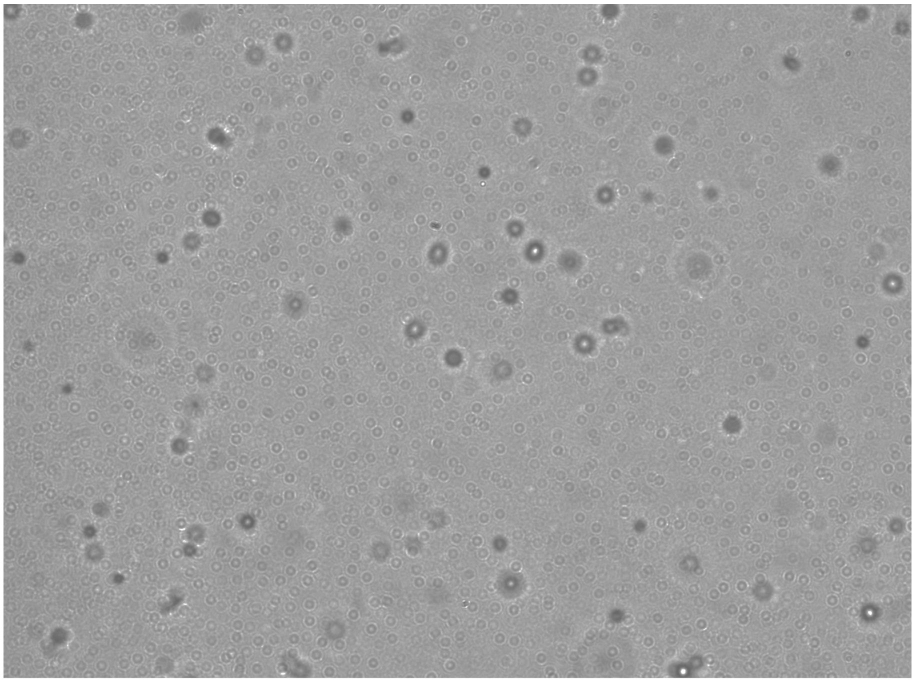

Supplementary Figure 15. Phase-contrast image of self-assembled nanospheres formed by hydration of a thin film of tris(triazole) Boc-*L*-^{H}Ala-[*L*-Trp]$_2$-OH (**7**).

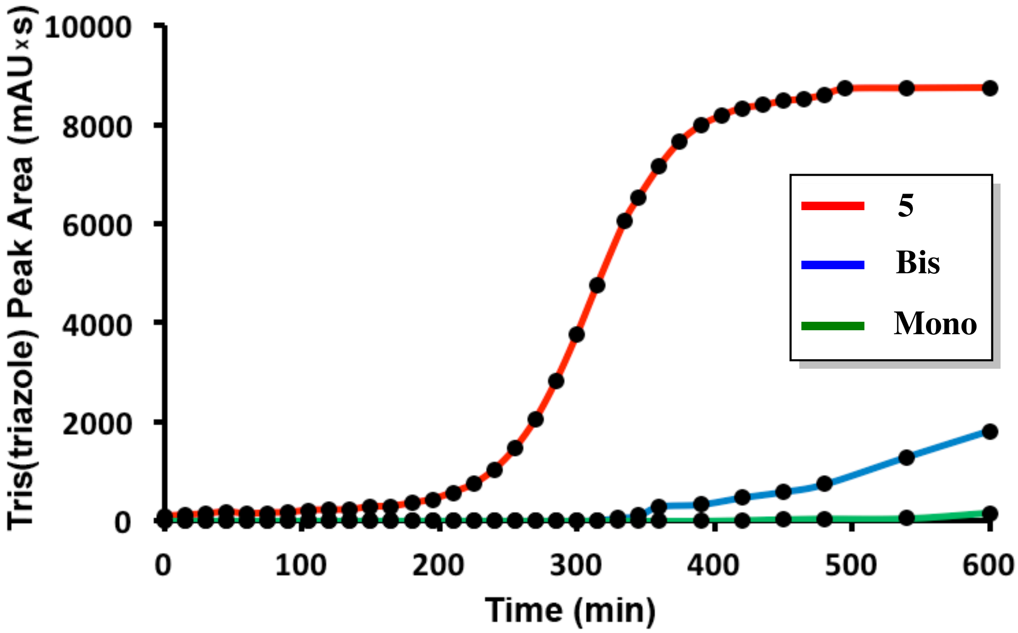

Supplementary Figure 16. Autocatalytic formation of tris(triazole) Boc-*L*-^{H}Ala-OH (**5**) by cycloaddition reaction of tripropargylamine with the azidoderivative Boc-*L*-^{H}Ala(N_3)-OH.DCHA (**1**) in the presence of 0.016 equiv. of tris(triazole) **5**, bis(triazole) Boc-*L*-^{H}Ala-OH (**Bis**) or mono(triazole) Boc-*L*-^{H}Ala-OH (**Mono**). HPLC spectra (210 nm) were used to monitor the progress of the CuAAC reaction. The retention times for all the species were verified by mass spectrometry and the use of known standards.

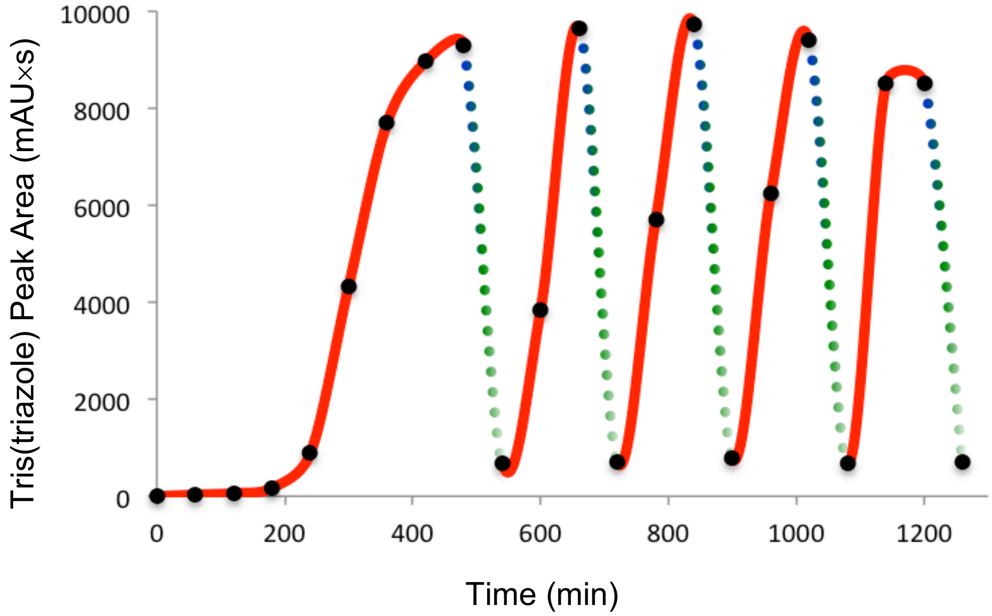

Supplementary Figure 17. Continual synthesis of catalytic peptide nanospheres through serial transfers. Tris(triazole) Boc-*L*-^{H}Ala-OH (**5**) formation (red line) with sequential serial transfers (10%) of nanospheres into fresh precursor solutions (blue/green degraded line) shows repeated and long-term oligotriazole peptide formation relative to controls lacking catalytic nanospheres (grey line). Tris(triazole) formation was monitored over time using combined liquid chromatography (LC), mass spectrometry (MS), and evaporative light-scattering detection (ELSD) measurements (raw LC data at 210 nm shown).

a)

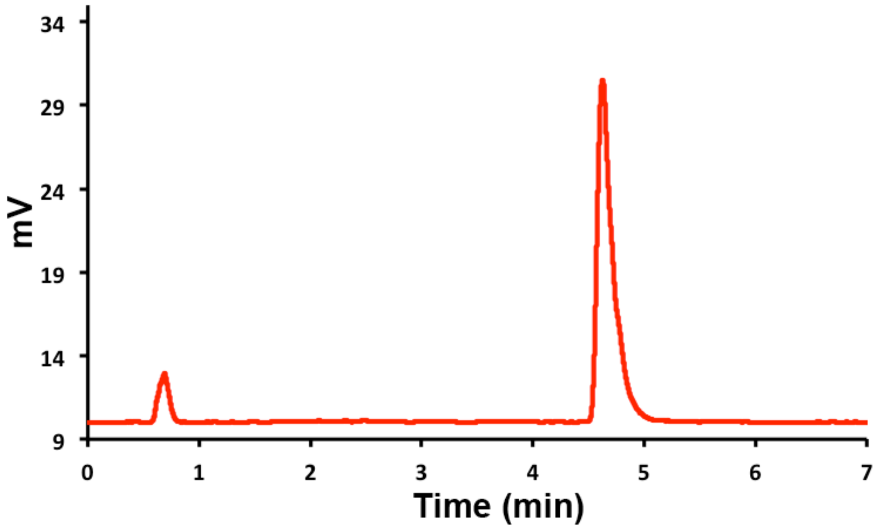

b)

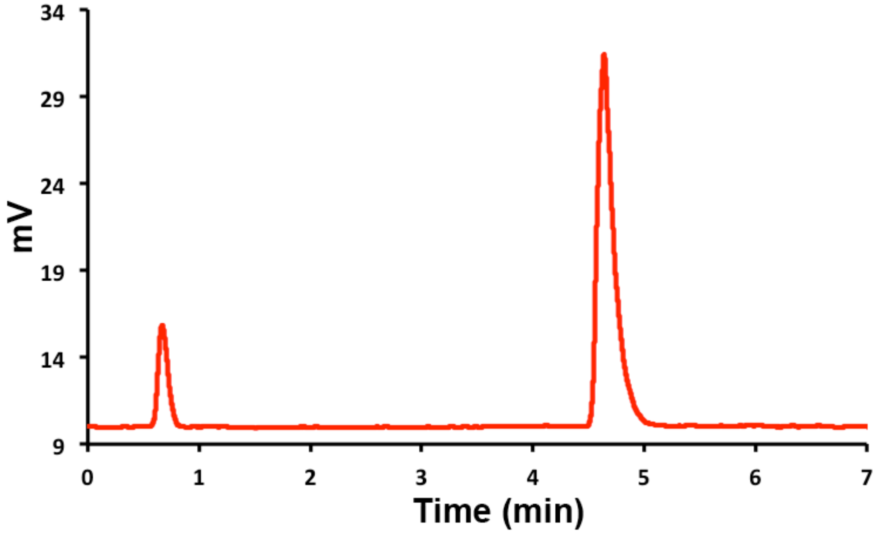

Supplementary Figure 18. Nature of the catalytically active species of the tris(triazole) peptide Boc-*L*-^{H}Ala-OH (**5**). ELSD traces of the filtrate (**a**) and nanospheres (**b**) solution, showing approximately the same concentration of tris(triazole) peptide **5**. Retention times were verified by mass spectrometry and the use of known standards.

a)

b)

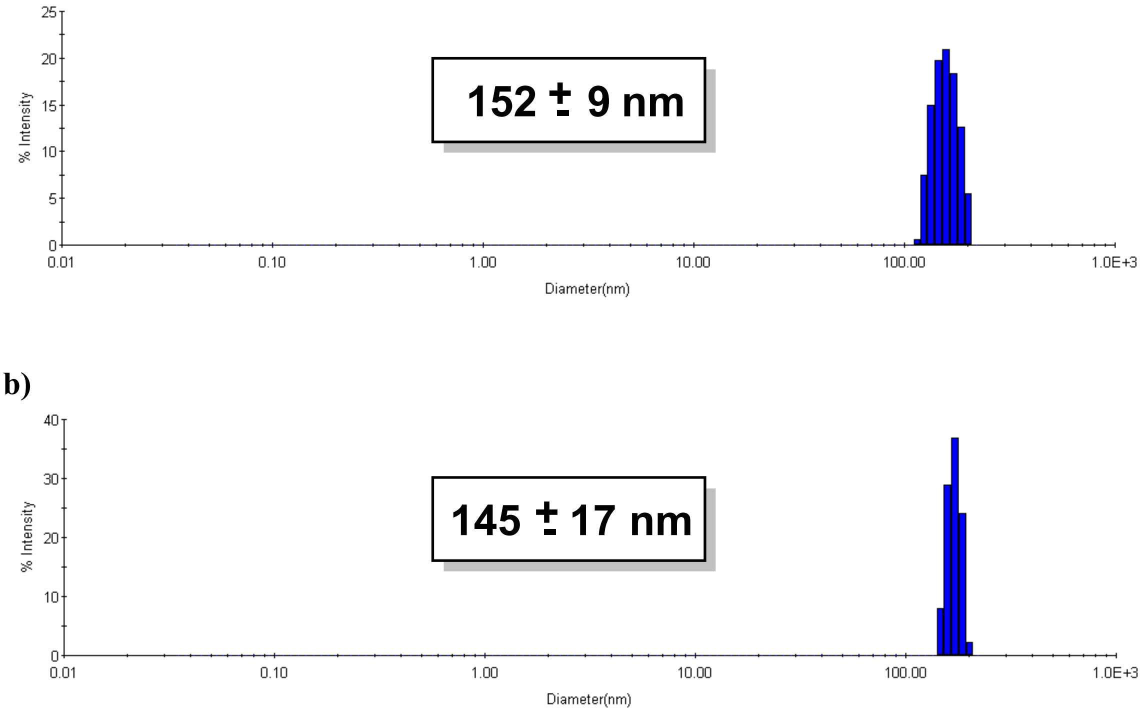

Supplementary Figure 19. Nature of the catalytically active species of the tris(triazole) peptide Boc-*L*-^{H}Ala-OH (**5**). Dynamic Light Scattering corresponding to the filtrate (**a**) and nanospheres (**b**) solution, showing approximately the same population sizes.

a) b)

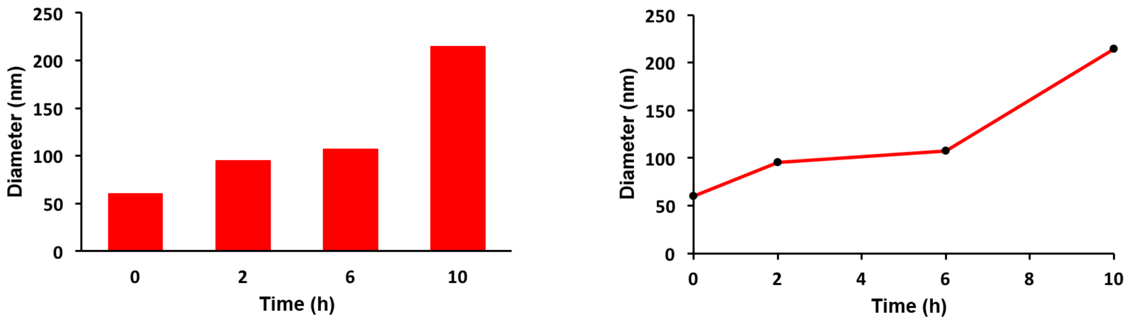

Supplementary Figure 20. Effect of the continued production of tris(triazole) on the nanospheres. Dynamic Light Scattering measurements over time shows the growth of the self-assembled peptide nanospheres formed from oligotriazole peptide **5** under standard CuAAC autocatalytic conditions.

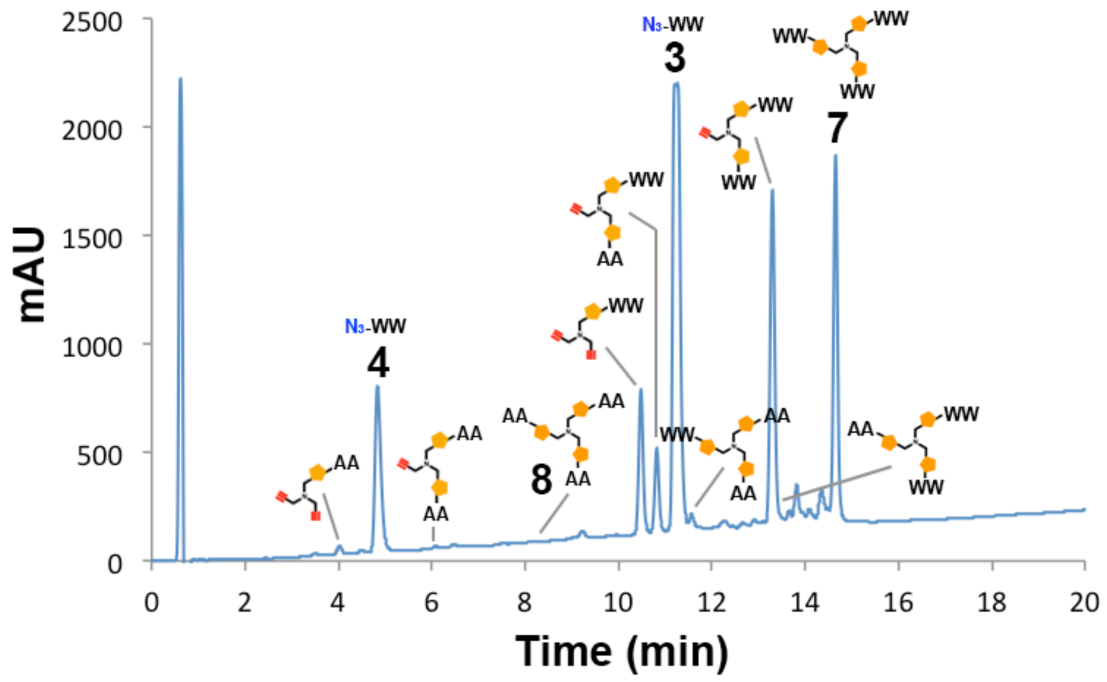

Supplementary Figure 21. Selective formation of tris(triazole) Boc-*L*-^{H}Ala-[*L*-Trp]$_2$-OH (**7**) [W: tryptophan; A: alanine].

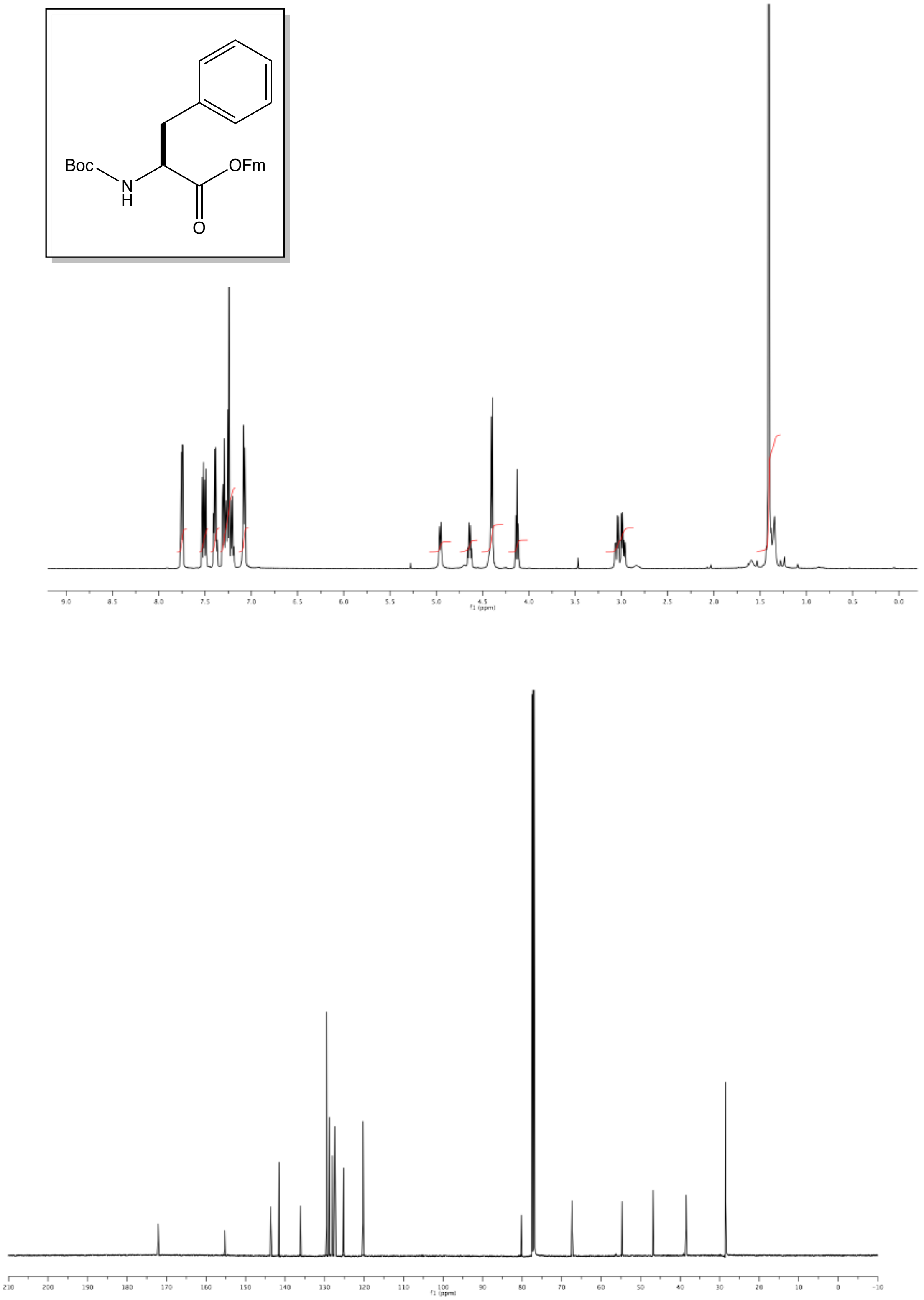

Supplementary Figure 22. ^{1}H NMR ($CDCl_3$, 500.13 MHz) (*top*) and ^{13}C NMR ($CDCl_3$, 125.77 MHz) (*bottom*) spectra of Boc-*L*-Phe-OFm.

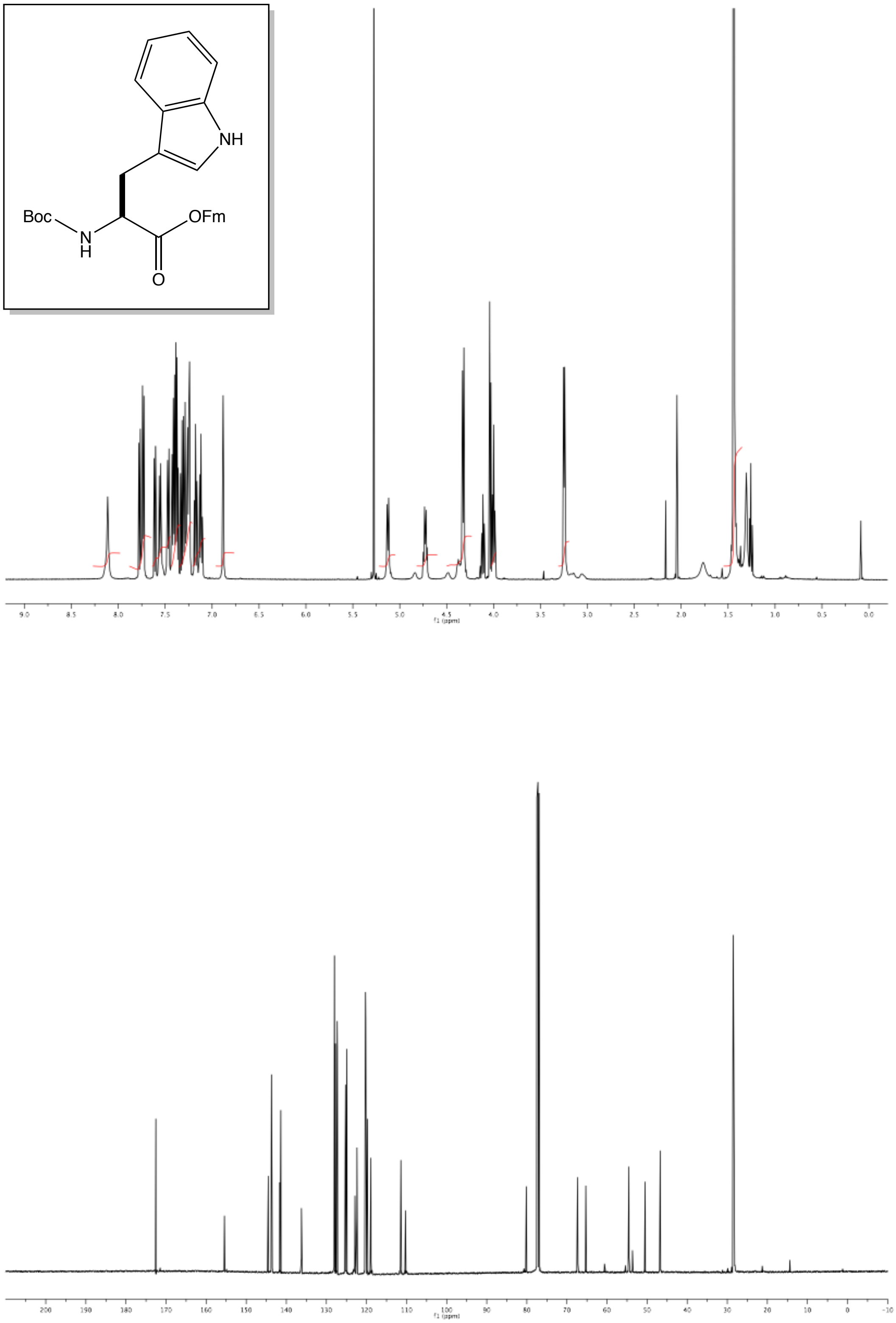

Supplementary Figure 23. ^{1}H NMR ($CDCl_3$, 500.13 MHz) (*top*) and ^{13}C NMR ($CDCl_3$, 125.77 MHz) (*bottom*) spectra of Boc-*L*-Trp-OFm.

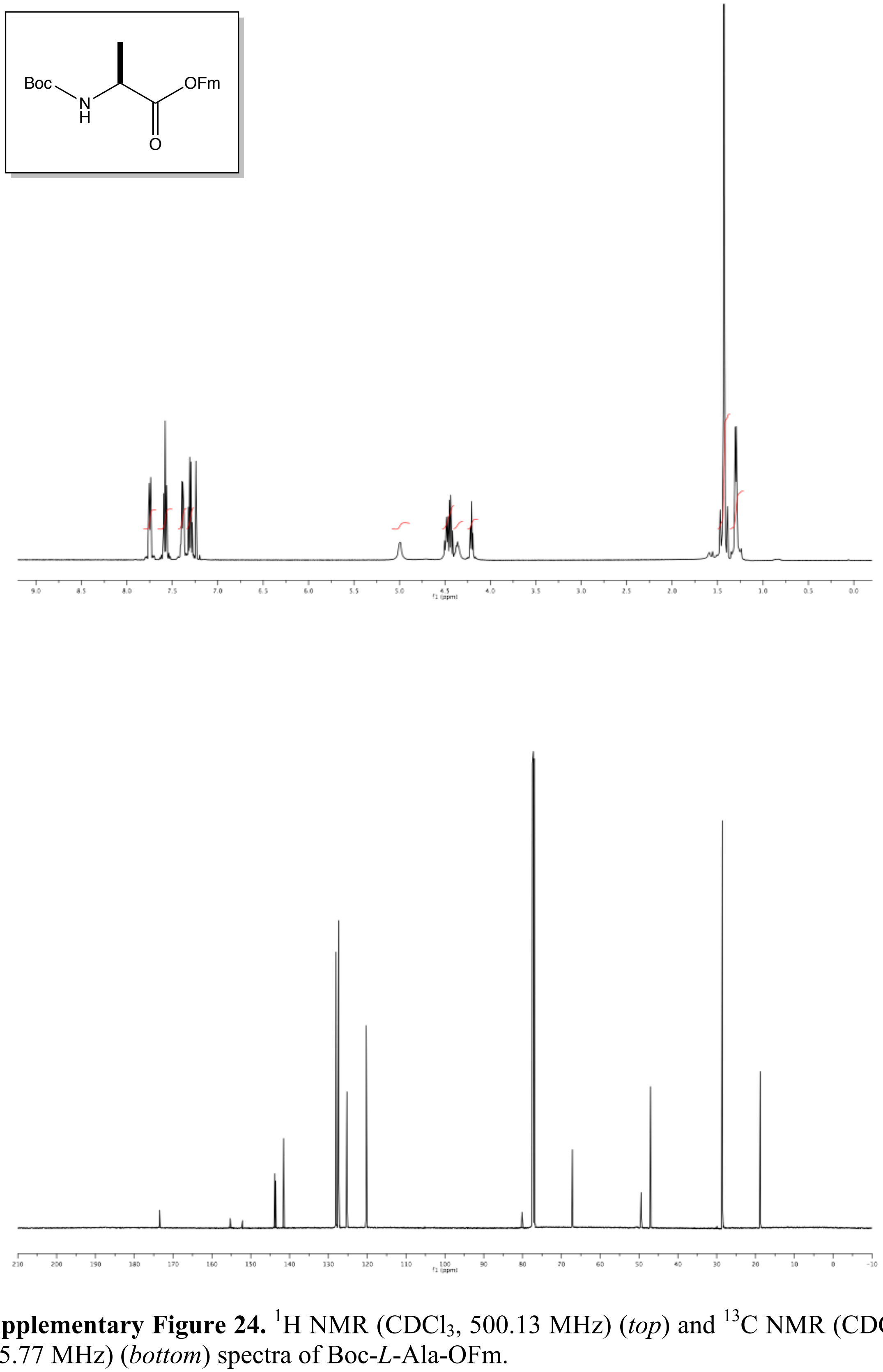

Supplementary Figure 24. ^{1}H NMR ($CDCl_3$, 500.13 MHz) (*top*) and ^{13}C NMR ($CDCl_3$, 125.77 MHz) (*bottom*) spectra of Boc-*L*-Ala-OFm.

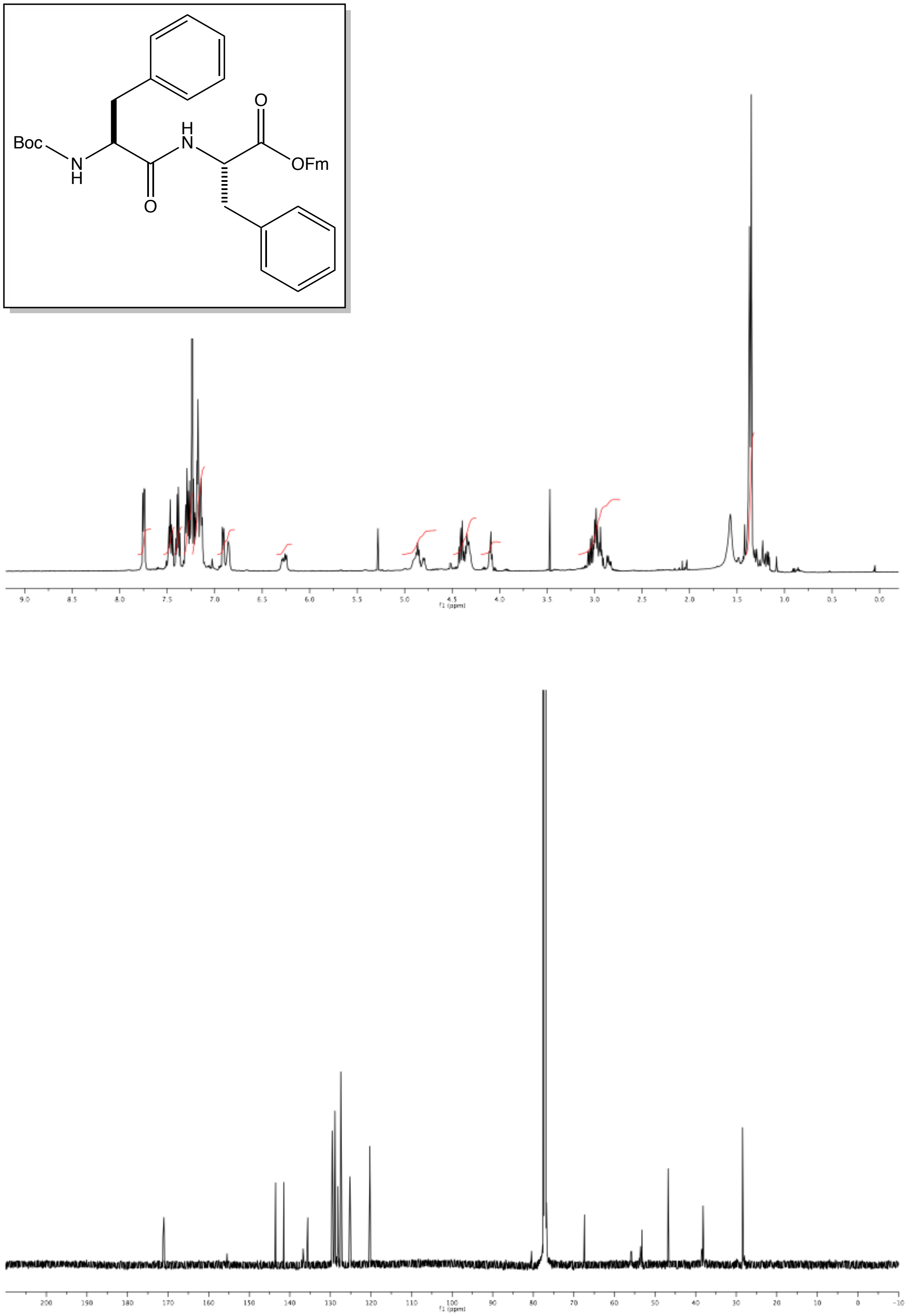

Supplementary Figure 25. ^{1}H NMR ($CDCl_3$, 500.13 MHz) (*top*) and ^{13}C NMR ($CDCl_3$, 125.77 MHz) (*bottom*) spectra of Boc-[*L*-Phe]$_2$-OFm.

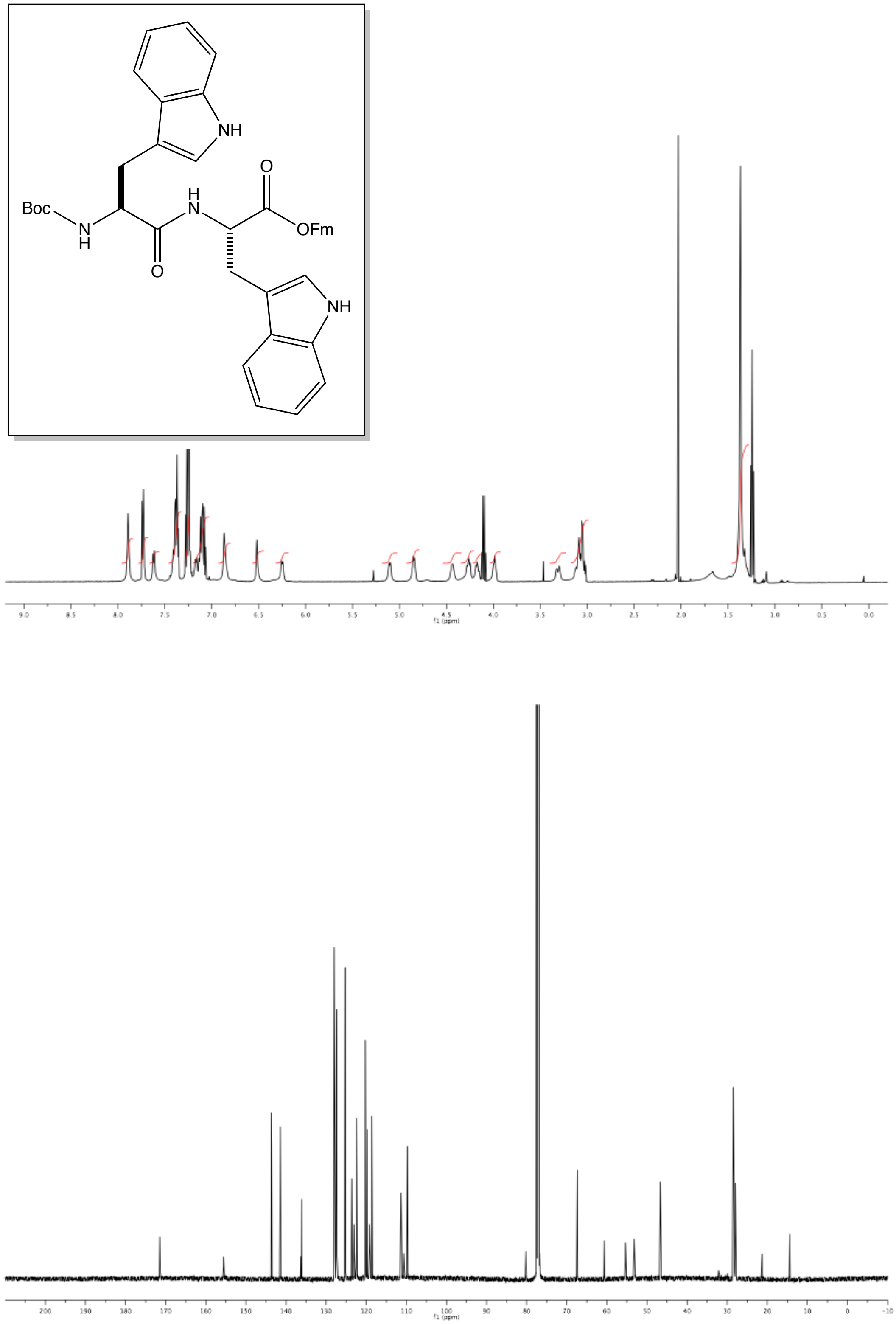

Supplementary Figure 26. ^{1}H NMR ($CDCl_3$, 500.13 MHz) (*top*) and ^{13}C NMR ($CDCl_3$, 125.77 MHz) (*bottom*) spectra of Boc-[*L*-Trp]$_2$-OFm.

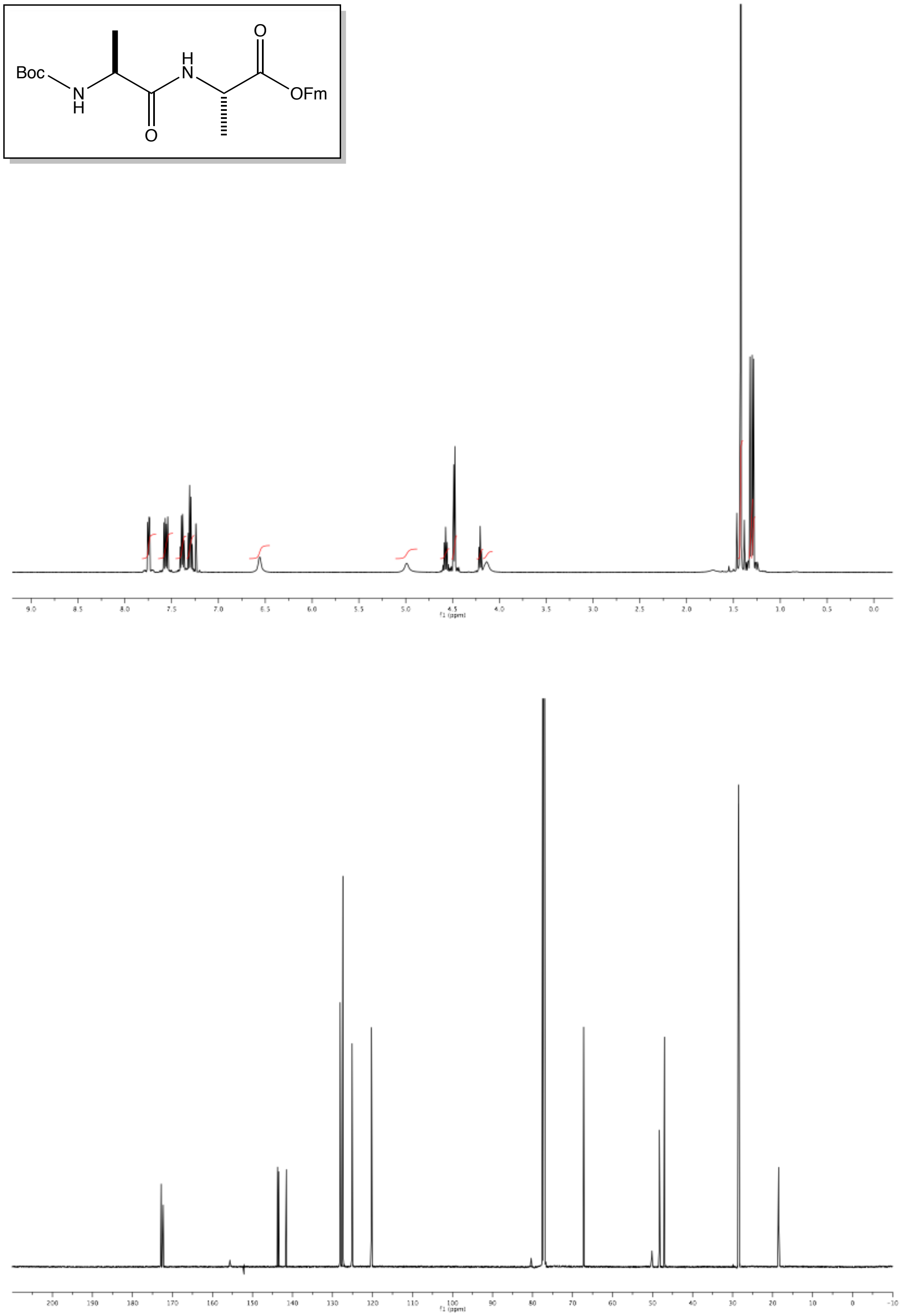

Supplementary Figure 27. ^{1}H NMR ($CDCl_3$, 500.13 MHz) (*top*) and ^{13}C NMR ($CDCl_3$, 125.77 MHz) (*bottom*) spectra of Boc-[*L*-Ala]$_2$-OFm.

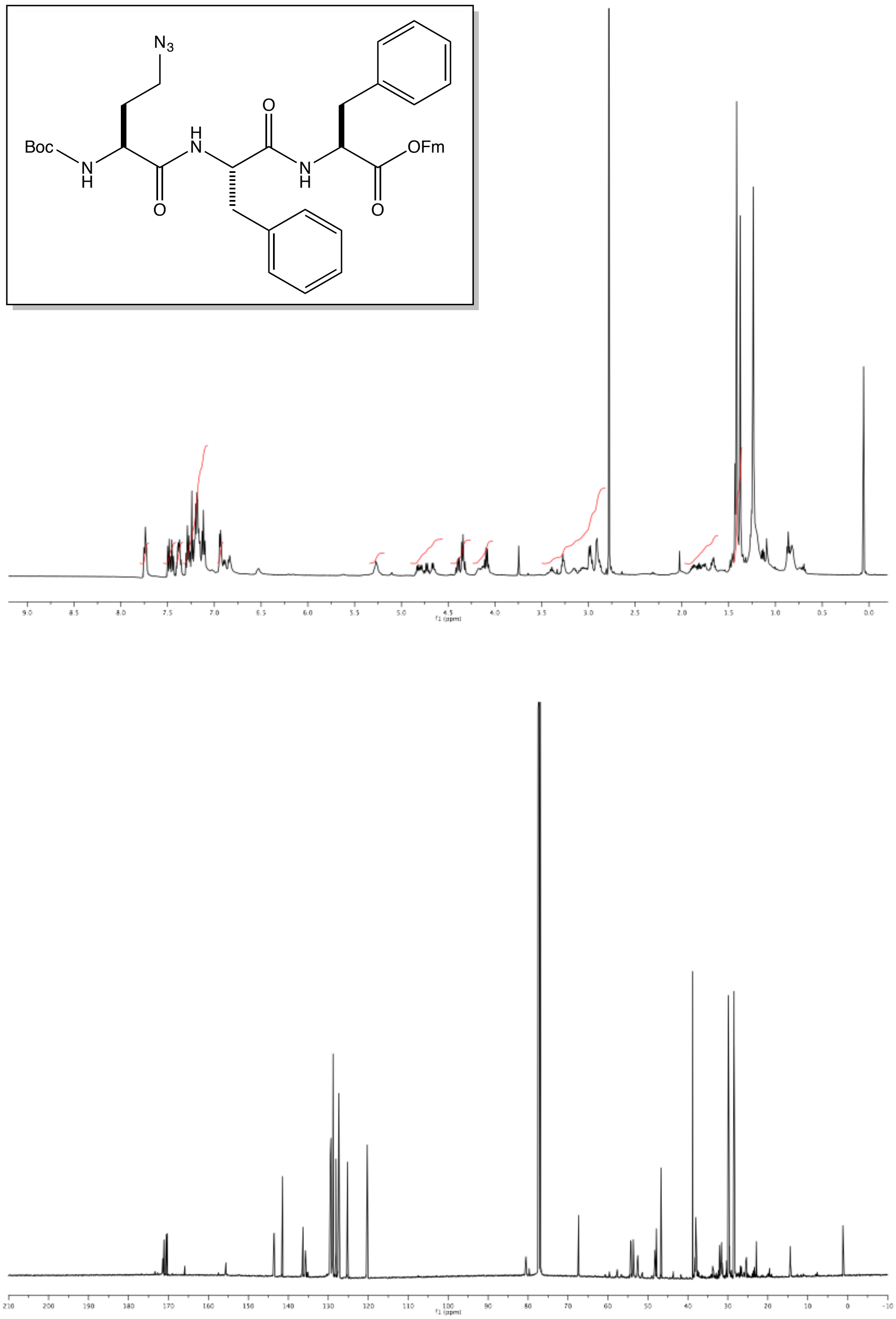

Supplementary Figure 28. ^{1}H NMR ($CDCl_3$, 500.13 MHz) (*top*) and ^{13}C NMR ($CDCl_3$, 125.77 MHz) (*bottom*) spectra of Boc-*L*-^{H}Ala(N_3)-[*L*-Phe]$_2$-OFm.

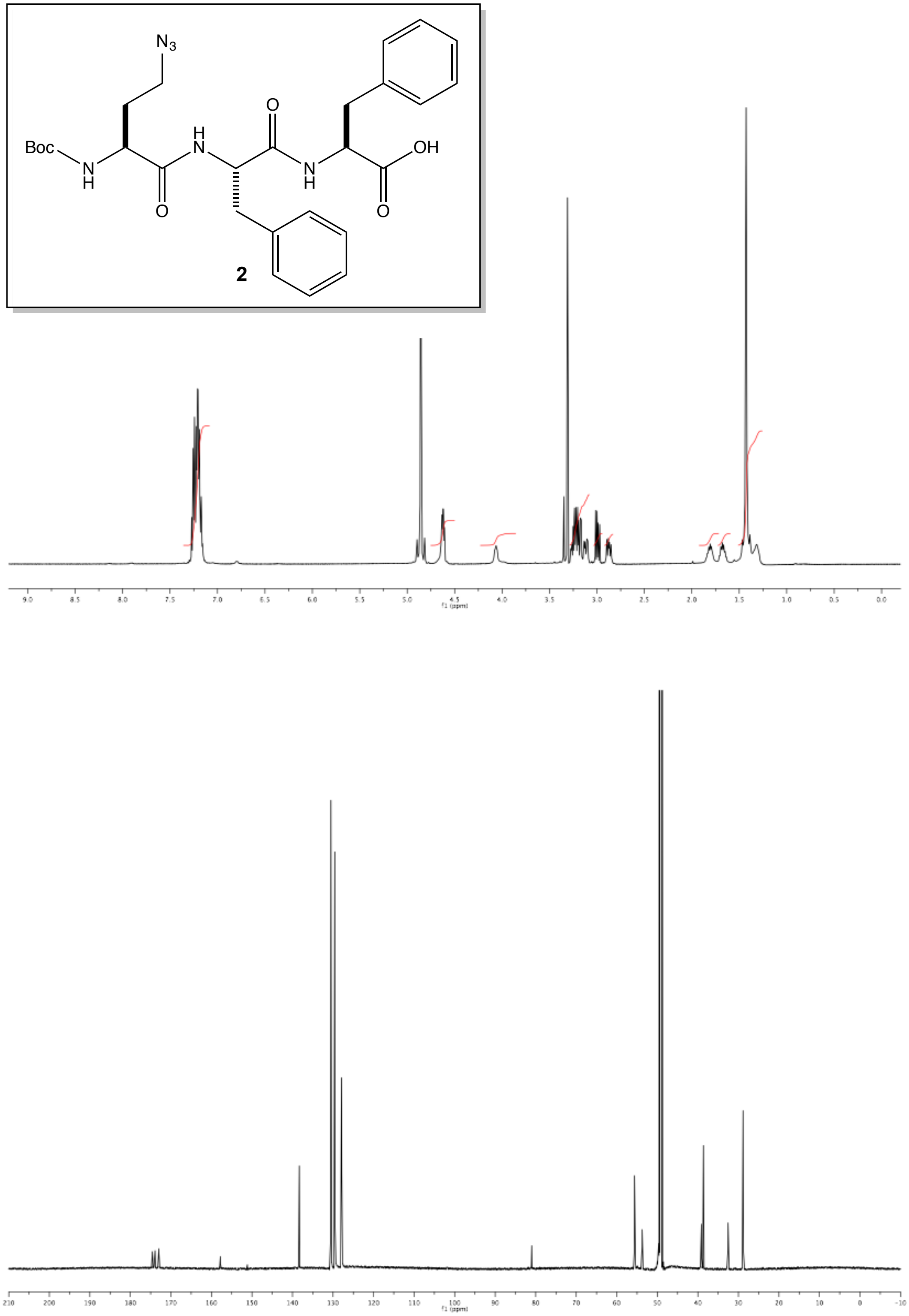

Supplementary Figure 29. ^{1}H NMR (CD_3OD, 500.13 MHz) (*top*) and ^{13}C NMR (CD_3OD, 125.77 MHz) (*bottom*) spectra of Boc-*L*-^{H}Ala(N_3)-[*L*-Phe]$_2$-OH (**2**).

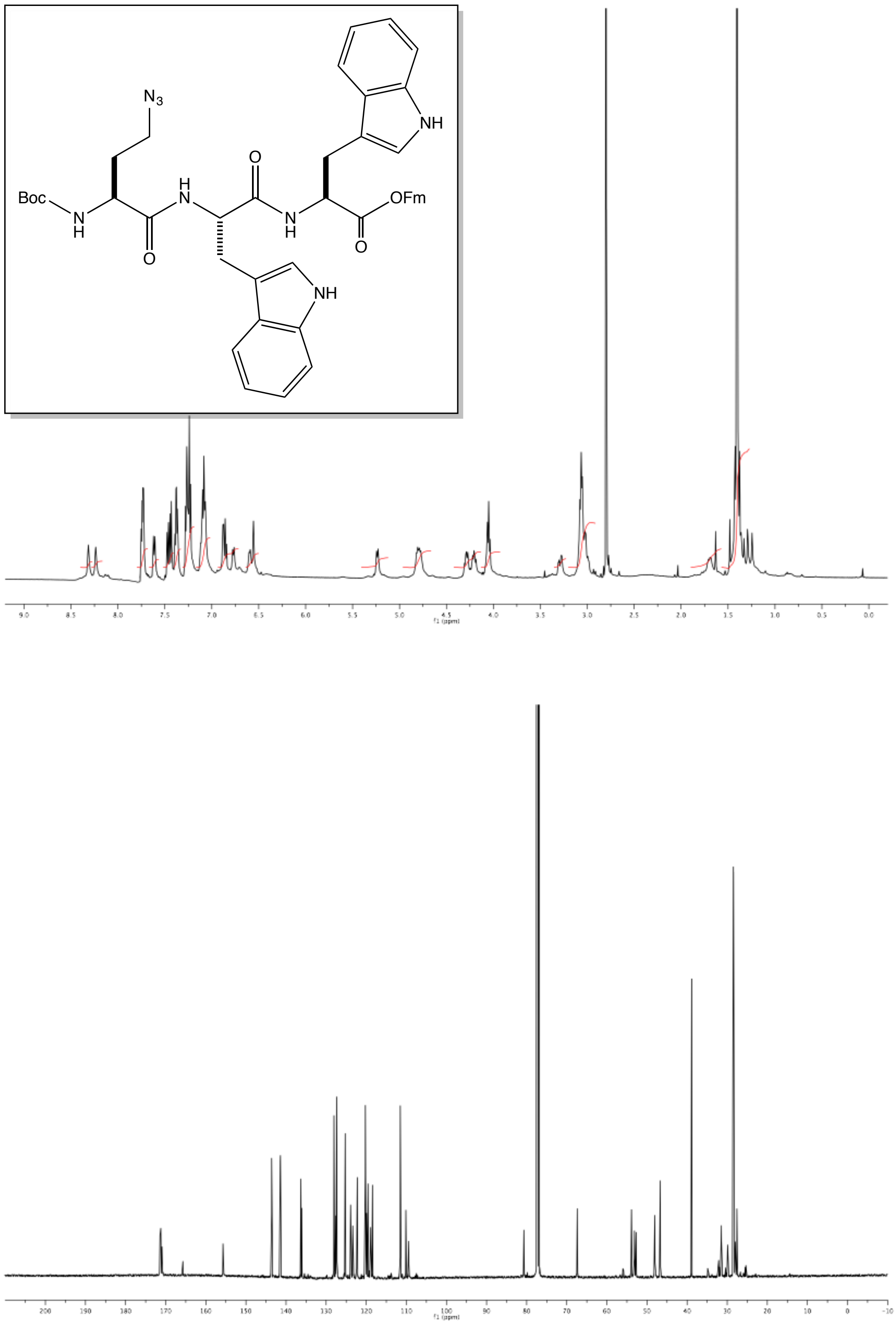

Supplementary Figure 30. ^{1}H NMR ($CDCl_3$, 500.13 MHz) (*top*) and ^{13}C NMR ($CDCl_3$, 125.77 MHz) (*bottom*) spectra of Boc-*L*-^{H}Ala(N_3)-[*L*-Trp]$_2$-OFm.

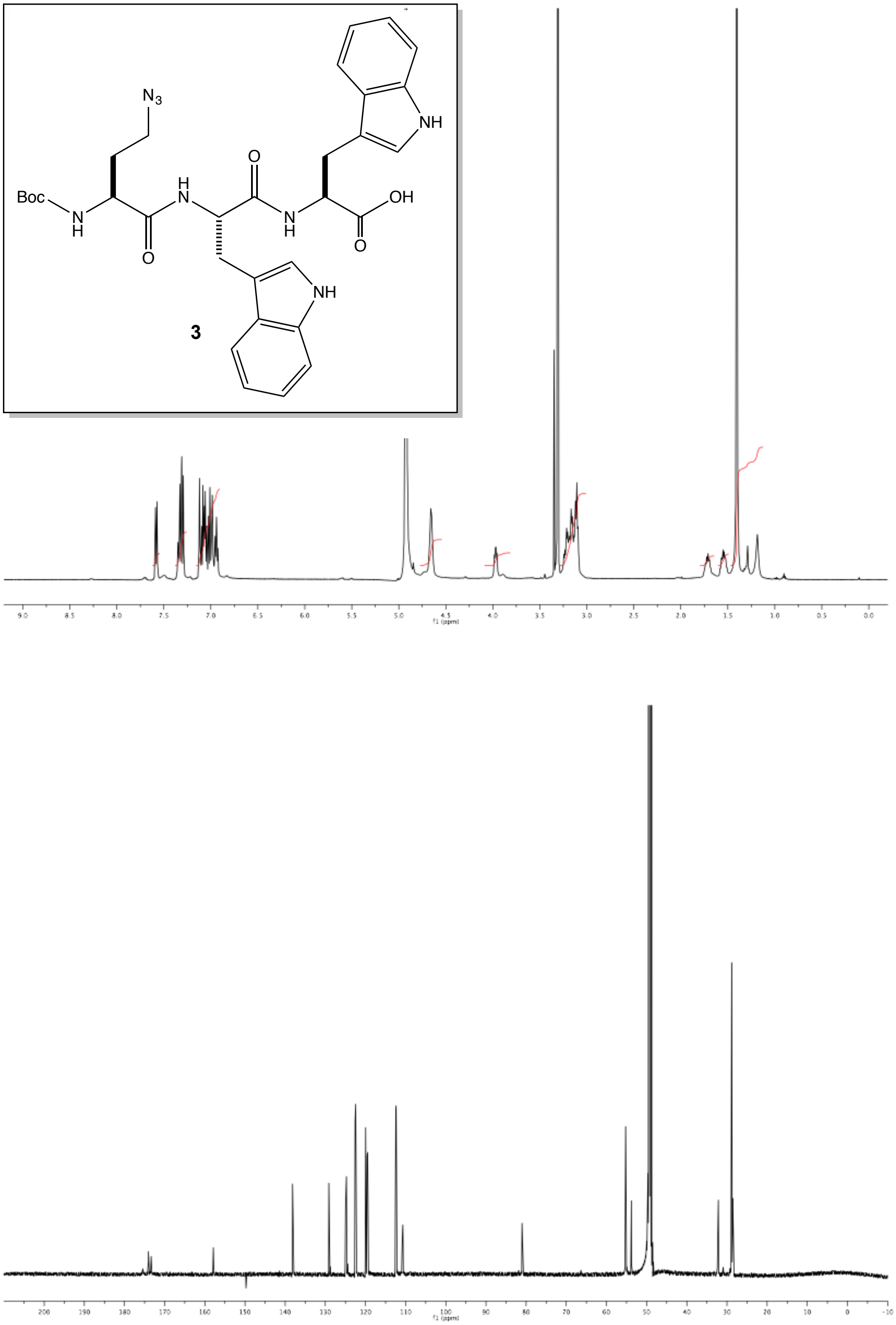

Supplementary Figure 31. ^{1}H NMR (CD_3OD, 500.13 MHz) (*top*) and ^{13}C NMR (CD_3OD, 125.77 MHz) (*bottom*) spectra of Boc-*L*-^{H}Ala(N_3)-[*L*-Trp]$_2$-OH (**3**).

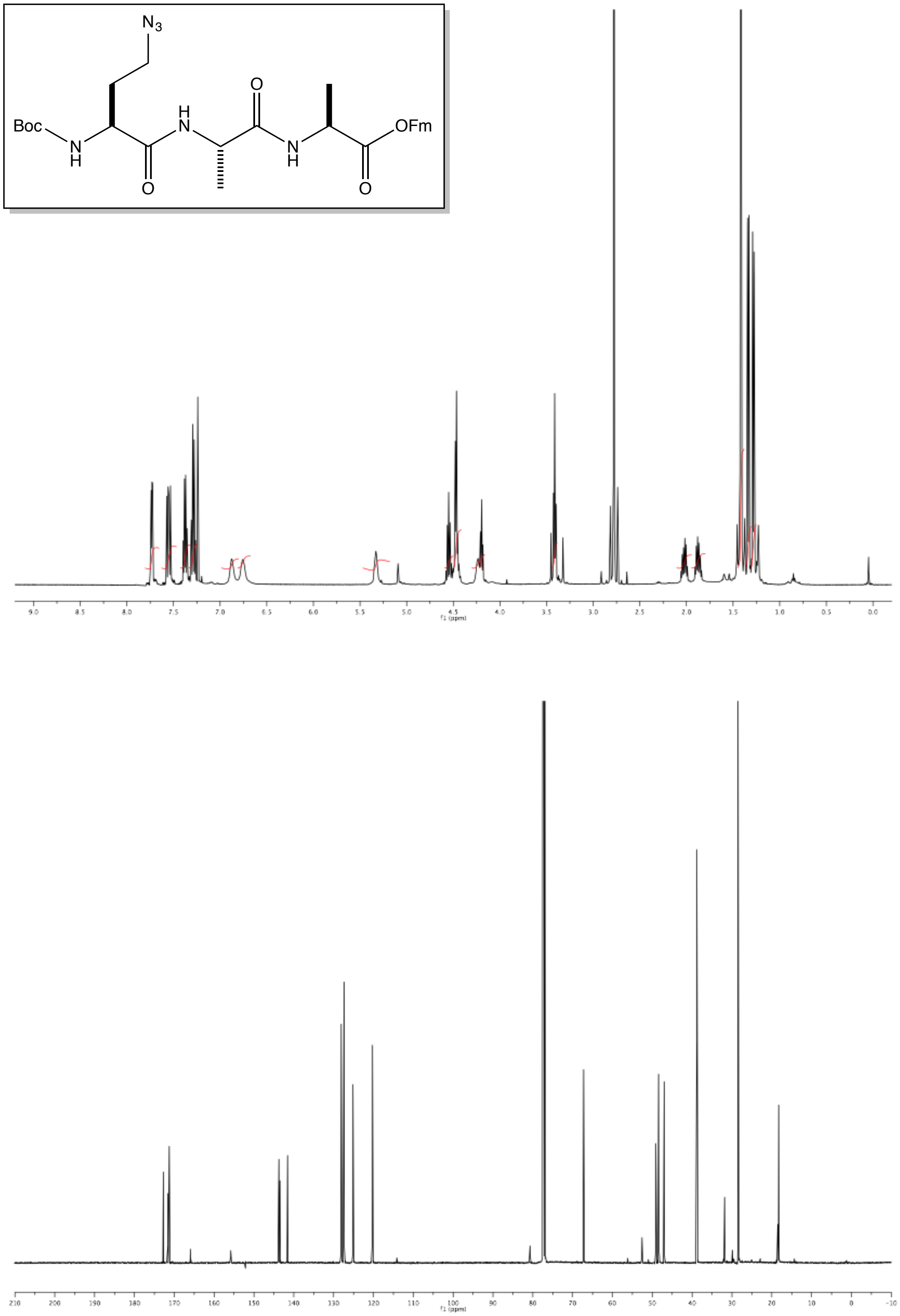

Supplementary Figure 32. ^{1}H NMR ($CDCl_3$, 500.13 MHz) (*top*) and ^{13}C NMR ($CDCl_3$, 125.77 MHz) (*bottom*) spectra of Boc-*L*-^{H}Ala(N_3)-[*L*-Ala]$_2$-OFm.

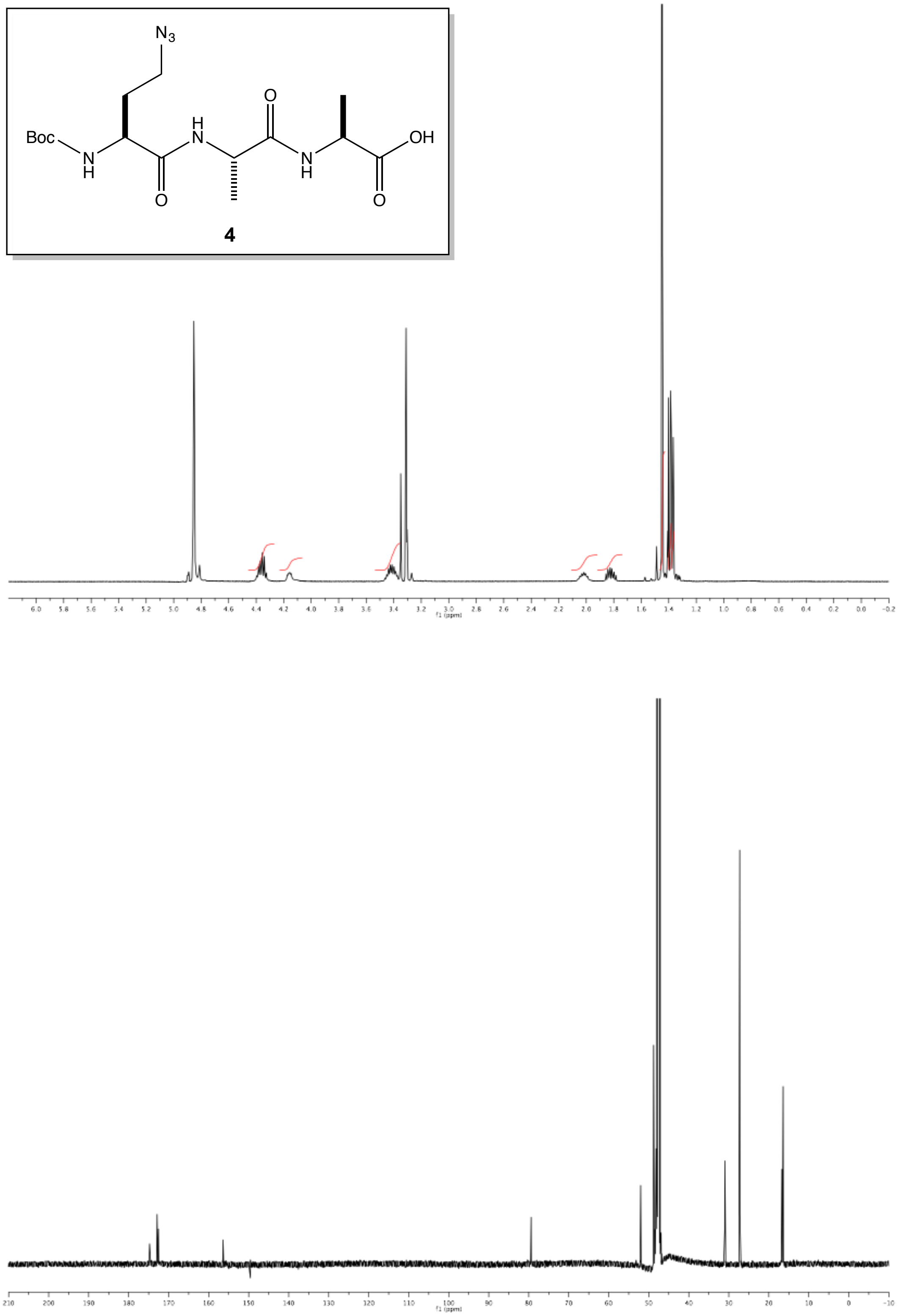

Supplementary Figure 33. ^{1}H NMR (CD_3OD, 500.13 MHz) (*top*) and ^{13}C NMR (CD_3OD, 125.77 MHz) (*bottom*) spectra of Boc-*L*-^{H}Ala(N_3)-[*L*-Ala]$_2$-OH (**4**).

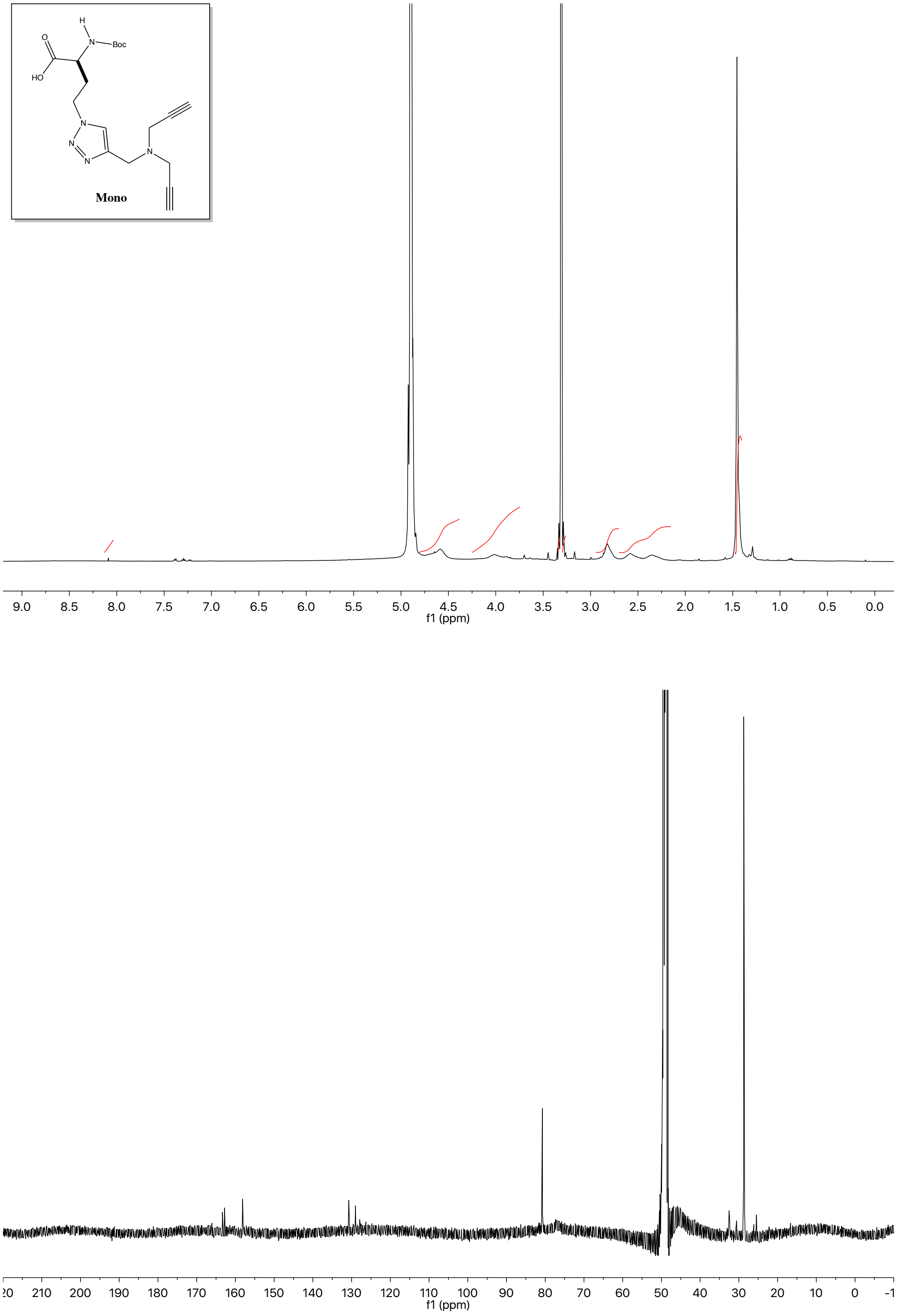

Supplementary Figure 34. ^{1}H NMR (CD_3OD, 500.13 MHz) (*top*) and ^{13}C NMR (CD_3OD, 125.77 MHz) (*bottom*) spectra of mono(triazole) Boc-*L*-^{H}Ala-OH (**Mono**).

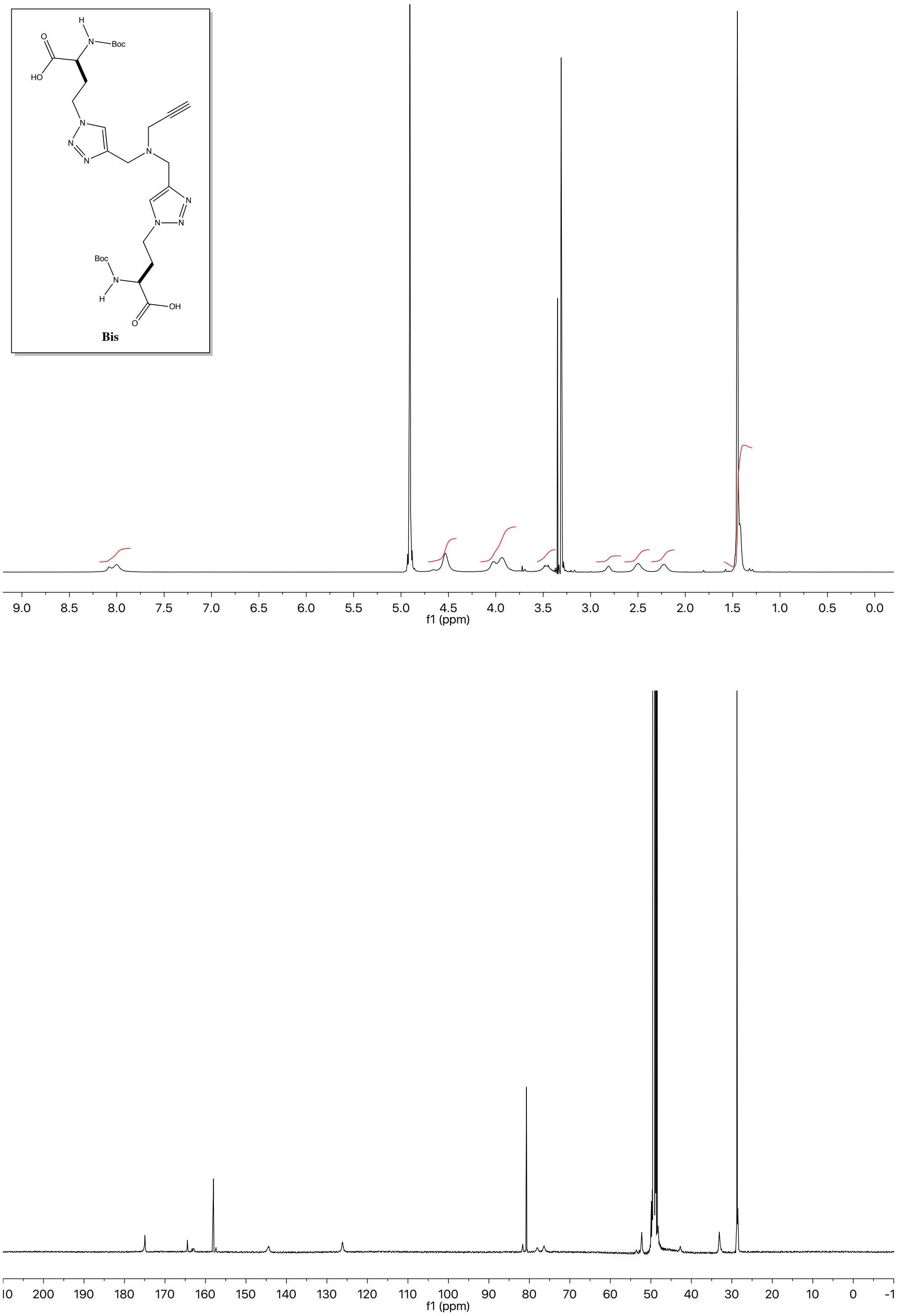

Supplementary Figure 35. ^{1}H NMR (CD_3OD, 500.13 MHz) (*top*) and ^{13}C NMR (CD_3OD, 125.77 MHz) (*bottom*) spectra of bis(triazole) Boc-*L*-^{H}Ala-OH (**Bis**).

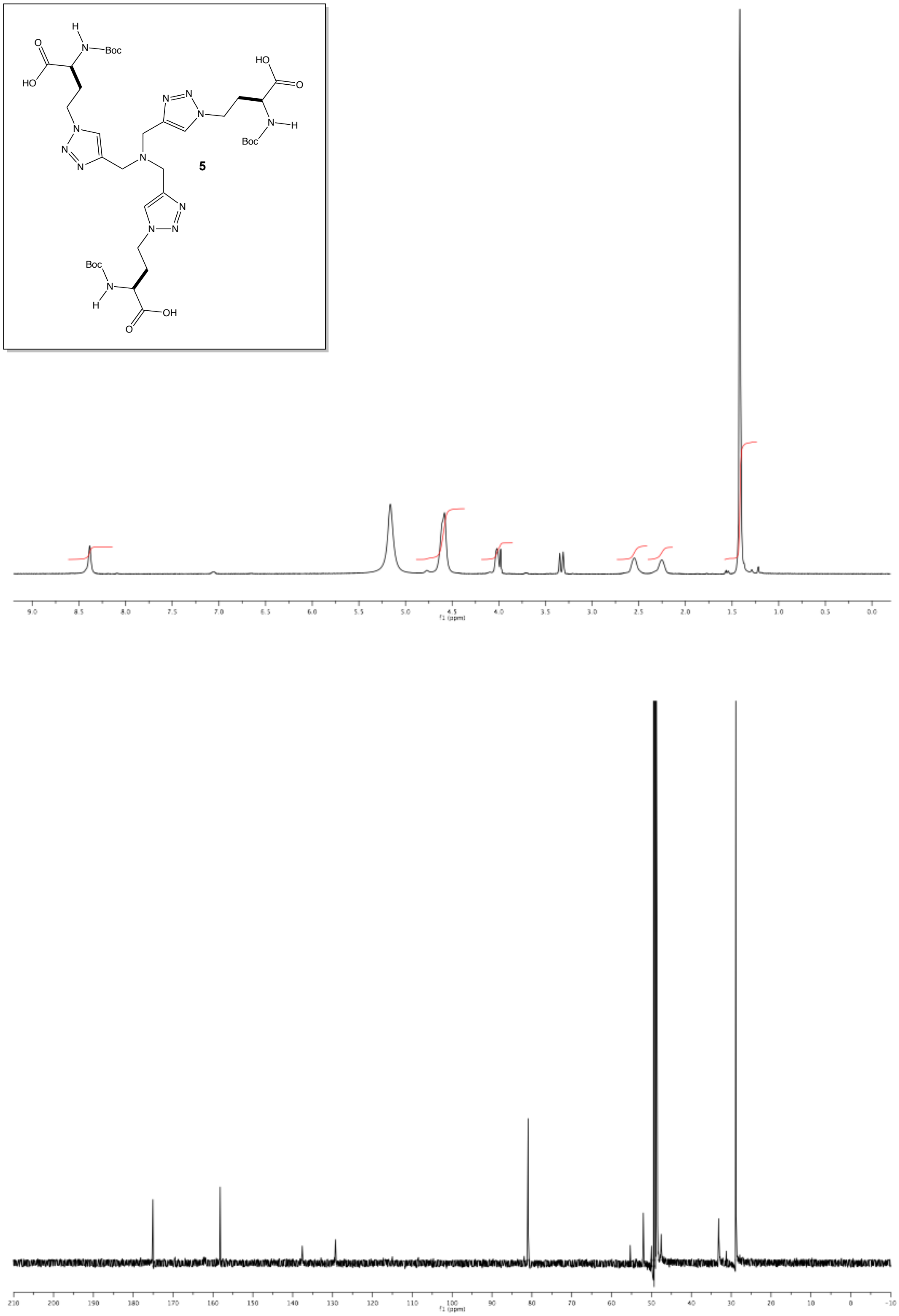

Supplementary Figure 36. ^{1}H NMR (CD_3OD, 500.13 MHz) (*top*) and ^{13}C NMR (CD_3OD, 125.77 MHz) (*bottom*) spectra of tris(triazole) Boc-*L*-^{H}Ala-OH (**5**).

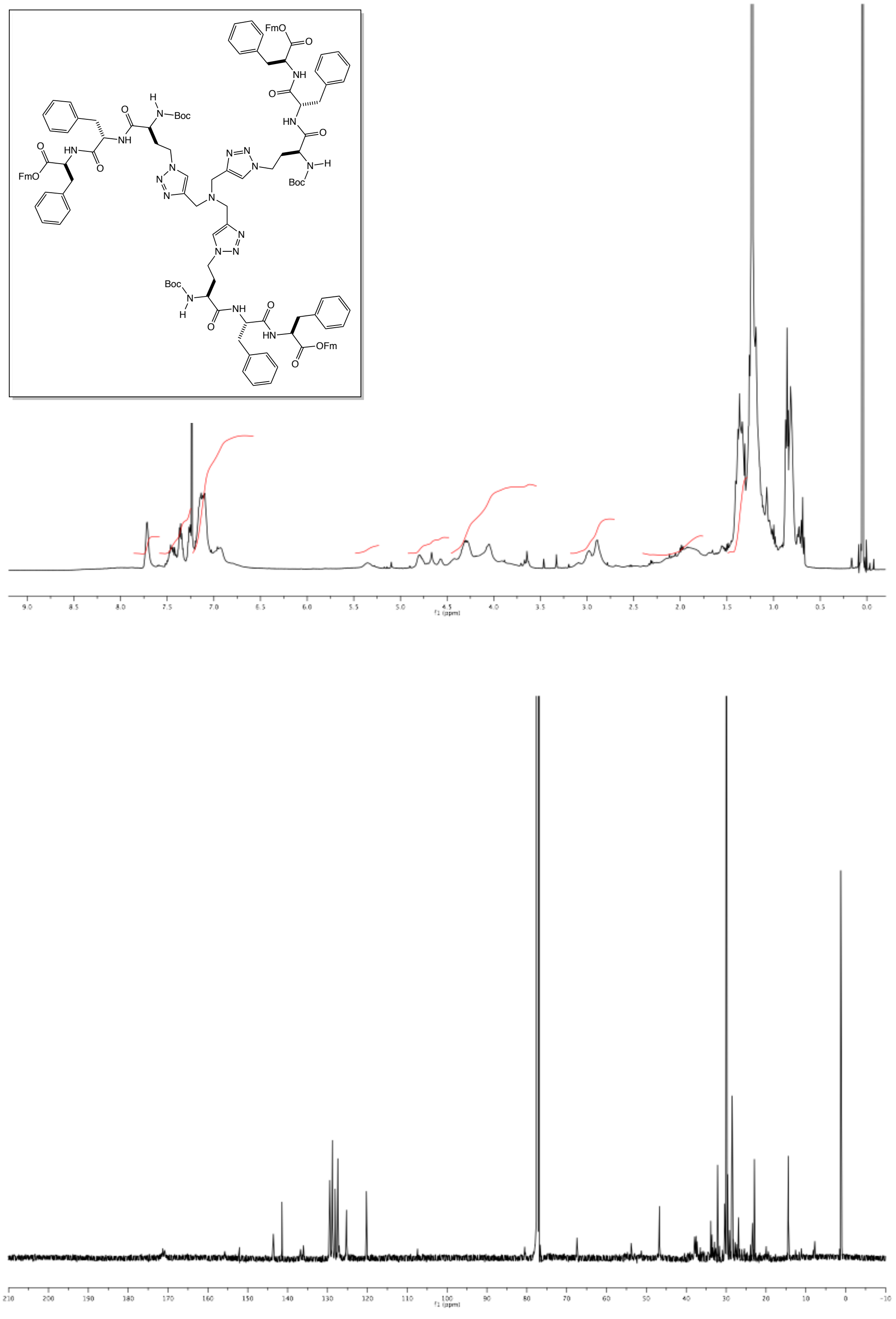

Supplementary Figure 37. ^{1}H NMR ($CDCl_3$, 500.13 MHz) (*top*) and ^{13}C NMR ($CDCl_3$, 125.77 MHz) (*bottom*) spectra of tris(triazole) Boc-*L*-^{H}Ala-[*L*-Phe]$_2$-OFm.

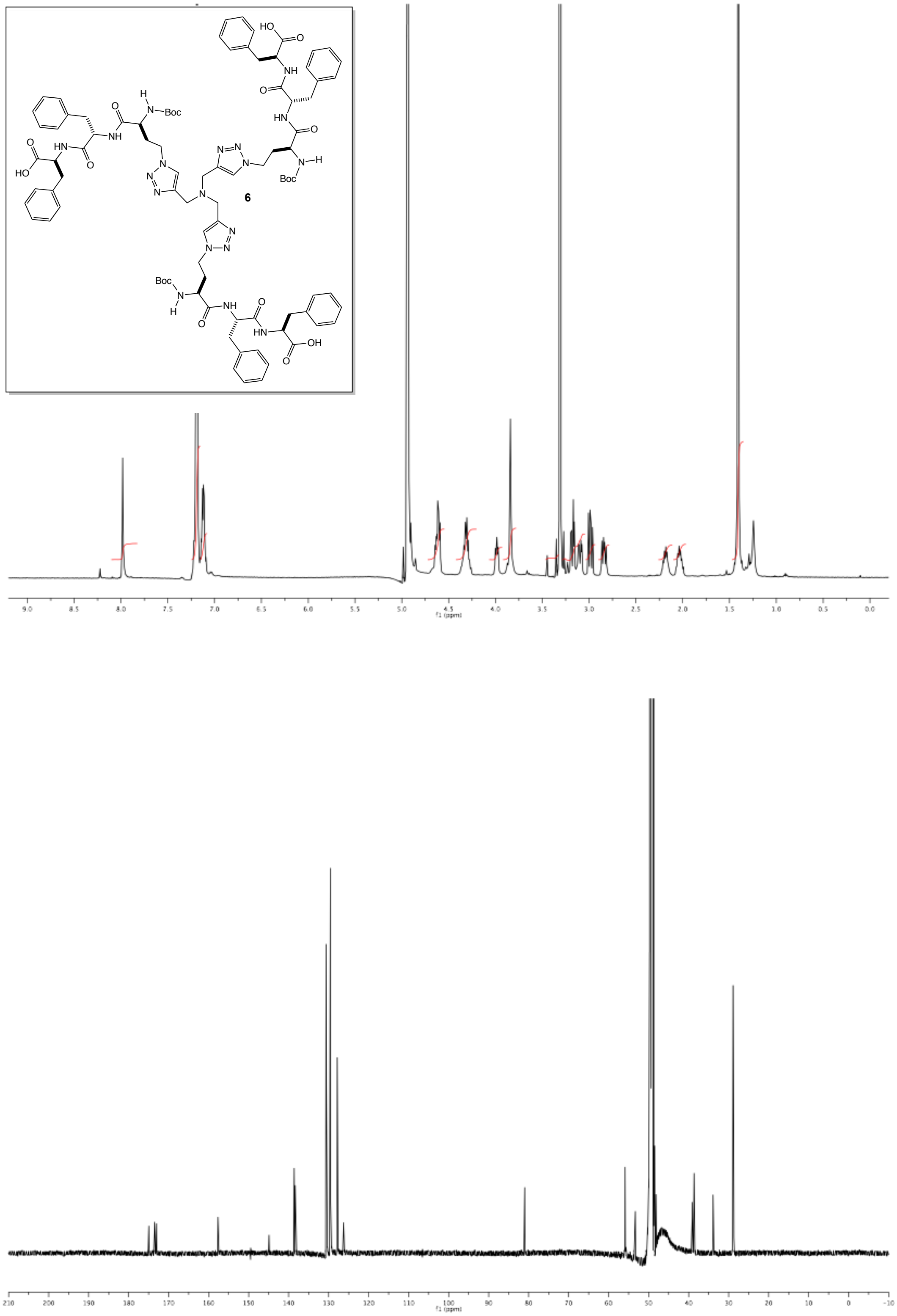

Supplementary Figure 38. ^{1}H NMR (CD_3OD, 500.13 MHz) (*top*) and ^{13}C NMR (CD_3OD, 125.77 MHz) (*bottom*) spectra of tris(triazole) Boc-*L*-^{H}Ala-[*L*-Phe]$_2$-OH (**6**).

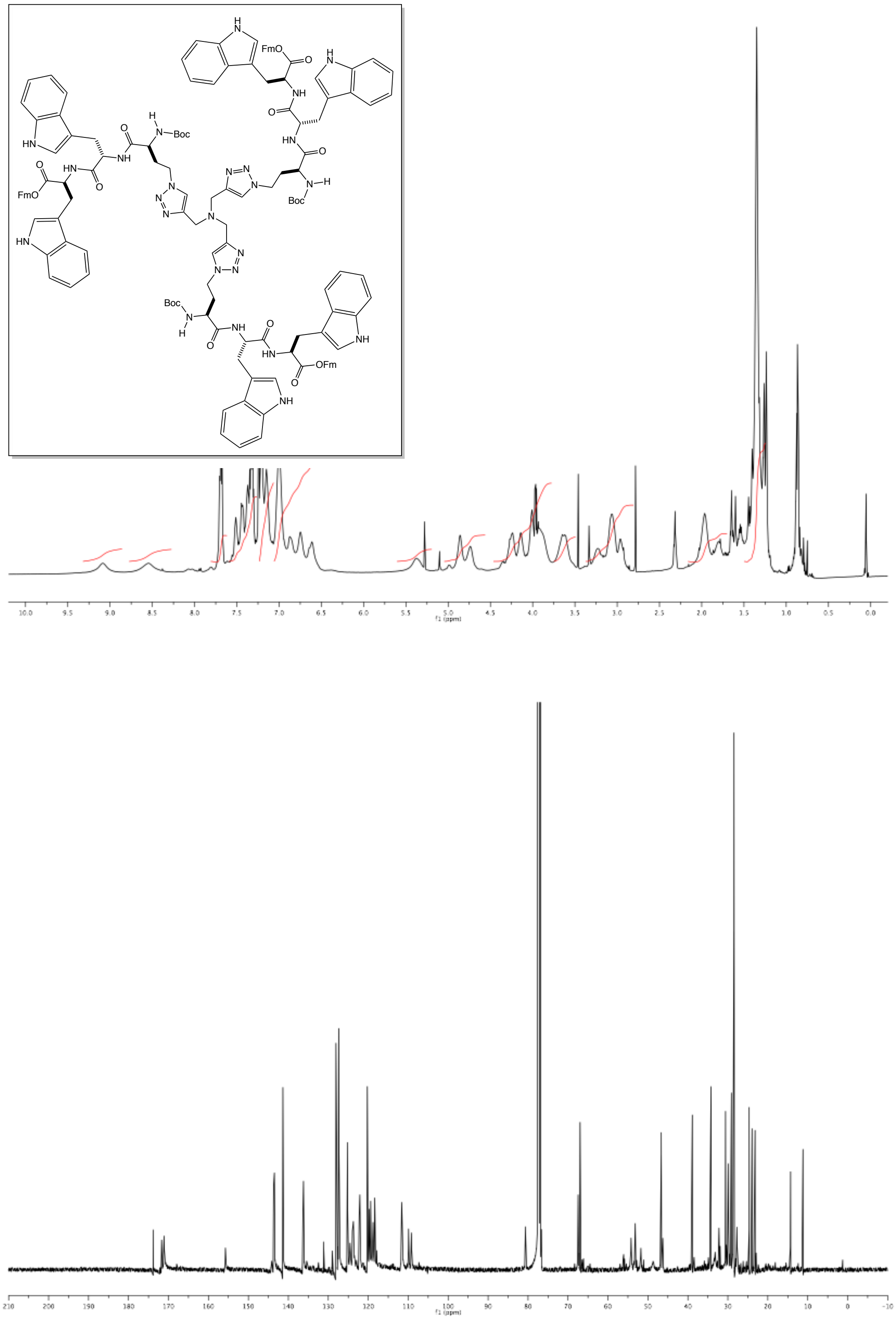

Supplementary Figure 39. ^{1}H NMR ($CDCl_3$, 500.13 MHz) (*top*) and ^{13}C NMR ($CDCl_3$, 125.77 MHz) (*bottom*) spectra of tris(triazole) Boc-*L*-^{H}Ala-[*L*-Trp]$_2$-OFm.

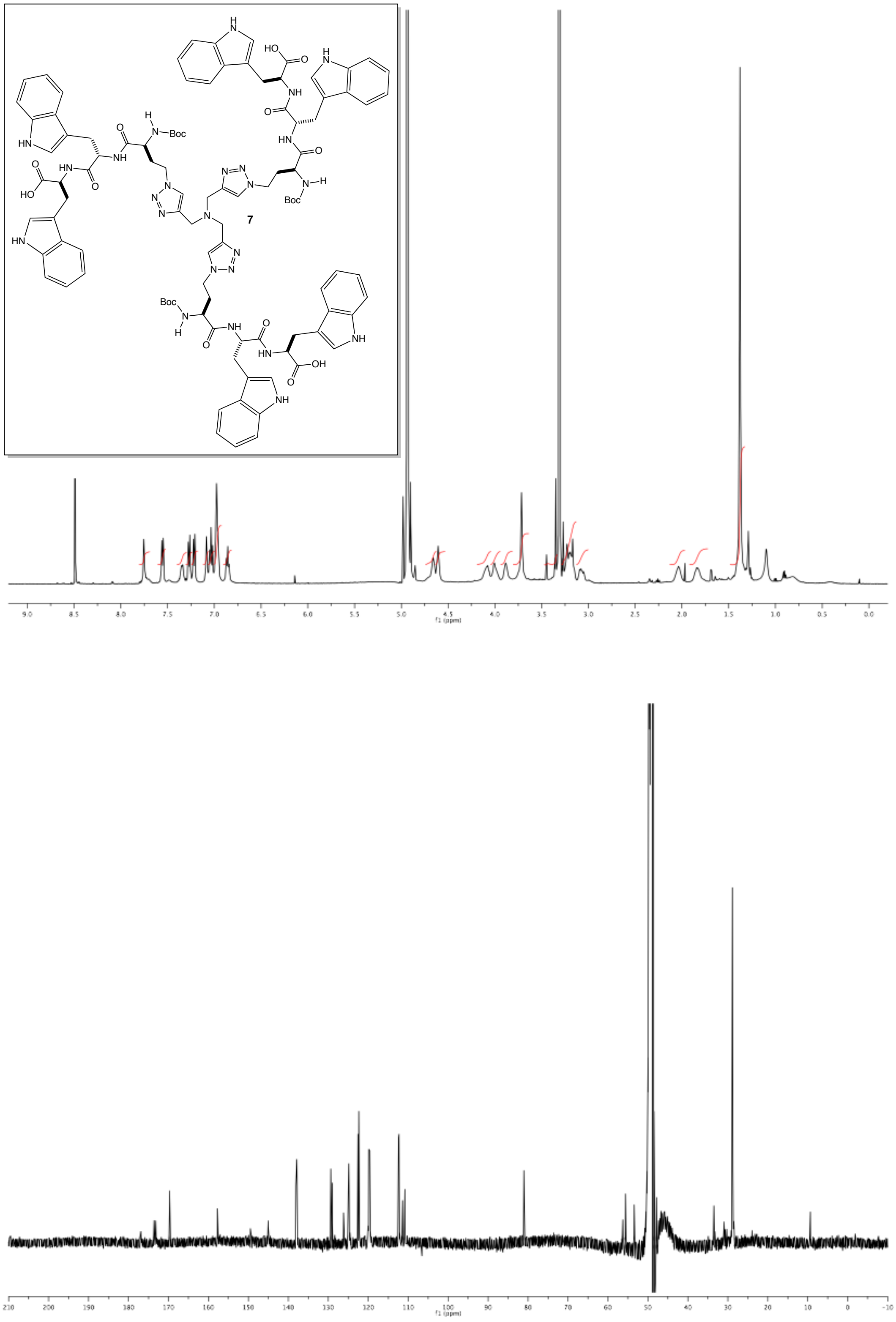

Supplementary Figure 40. ^{1}H NMR (CD_3OD, 500.13 MHz) (*top*) and ^{13}C NMR (CD_3OD, 125.77 MHz) (*bottom*) spectra of tris(triazole) Boc-*L*-^{H}Ala-[*L*-Trp]$_2$-OH (**7**).

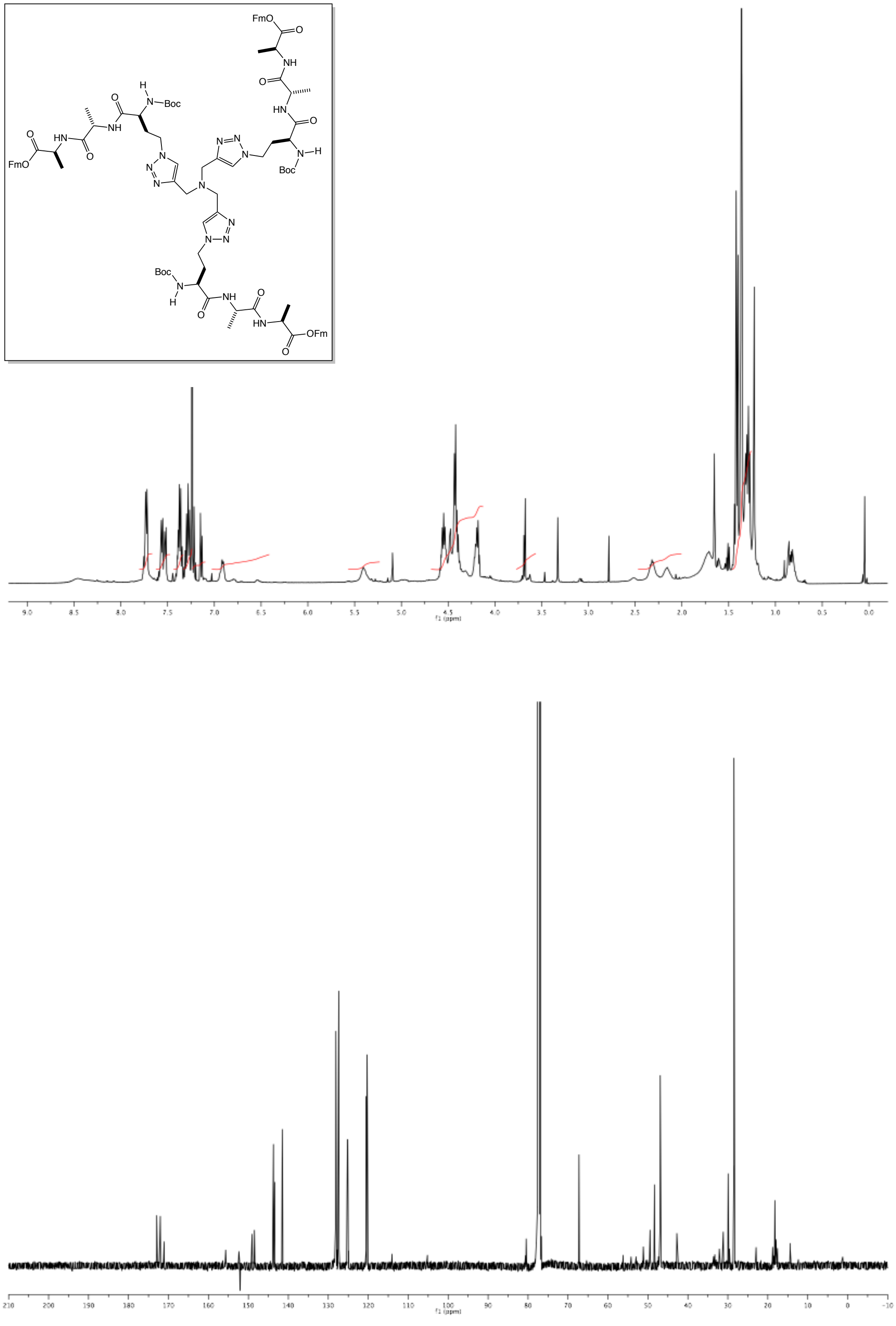

Supplementary Figure 41. ^{1}H NMR ($CDCl_3$, 500.13 MHz) (*top*) and ^{13}C NMR ($CDCl_3$, 125.77 MHz) (*bottom*) spectra of tris(triazole) Boc-*L*-^{H}Ala-[*L*-Ala]$_2$-OFm.

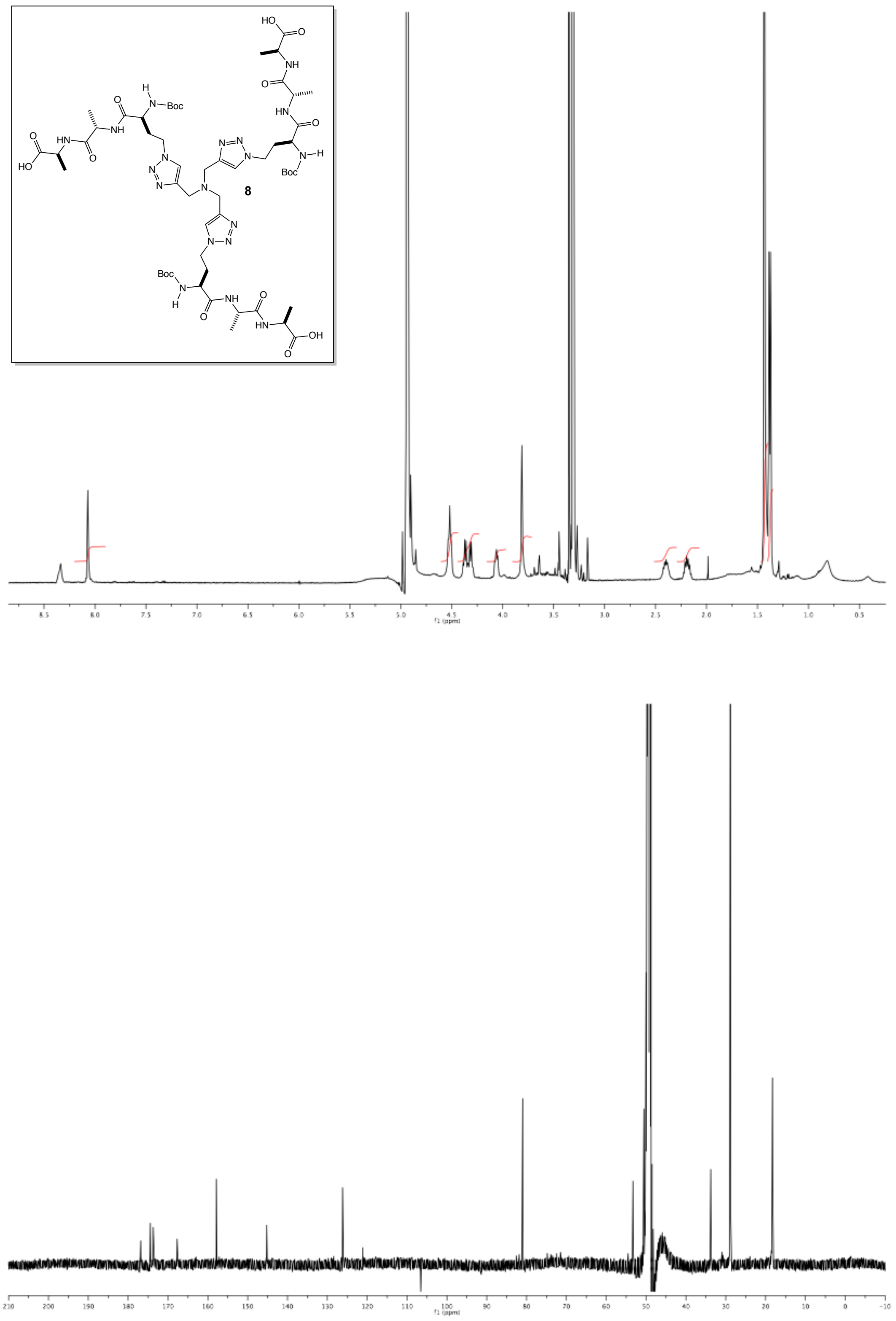

Supplementary Figure 42. ^{1}H NMR (CD_3OD, 500.13 MHz) (*top*) and ^{13}C NMR (CD_3OD, 125.77 MHz) (*bottom*) spectra of tris(triazole) Boc-*L*-^{H}Ala-[*L*-Ala]$_2$-OH (**8**).

Supplementary References

[1] Brea, R. J., Amorin, M., Castedo, L. & Granja, J. R. Methyl-blocked dimeric α,γ-peptide nanotube segments: formation of a peptide heterodimer through backbone-backbone interactions. *Angew. Chem. Int. Ed.* **44**, 5710-5713 (2005).

[2] Brea, R. J., Castedo, L. & Granja, J. R. Large-diameter self-assembled dimers of α,γ-cyclic peptides, with the nanotubular solid-state structure of cyclo-[(*L*-Leu-*D*-^{Me}N-γ-Acp)$_4$-]·4$CHCl_2COOH$. *Chem. Commun.*, 3267-3269 (2007).
